# Supplementary material for: Evidence for dynastic succession among early Celtic elites in Central Europe
Source: Nat Hum Behav. 2024 Jun 3;8(8):1467–80. doi: 10.1038/s41562-024-01888-7 (PMC11343710; doi:10.1038/s41562-024-01888-7)
Supplement: Supplementary file 1 — This file contains the following five sections: Supplementary Note 1: site descriptions; Supplementary Note 2: kinship, inbreeding, individual ancestry, isotopes and height; Supplementary Note 3: latent pedigree modelling; Supplementary Note 4: the formation of the Hallstatt gene pool; Supplementary Note 5: population genetic changes after the Hallstatt period. The file also includes Supplementary Figs. 1.1–5.8 and additional references. [file 41562_2024_1888_MOESM1_ESM.pdf]

nature human behaviour

Supplementary information https://doi.org/10.1038/s41562-024-01888-7

Evidence for dynastic succession among early Celtic elites in Central Europe

In the format provided by the authors and unedited

Supplementary Information Guide

Supplementary Note 1: Site descriptions **2**
The Asperg-Grafenbühl burial mound 2
The Römerhügel burial mound 5
The Early Celtic centre of power at the Heuneburg 8
Landscaping at the Alte Burg 12
The Eberdingen-Hochdorf burial mound 16
The Magdalenenberg burial mound near Villingen 21
The female burial of Ditzingen-Schöckingen 24
Supplementary Note 2: Kinship, Inbreeding and individual ancestry **28**
Kinship 28
mtDNA haplogroups 32
Inbreeding 33
Individual ancestry 35
Isotopes 37
Height 39
Comparing archaeological, isotopic and aDNA data 40
Supplementary Note 3: Latent pedigree modelling **42**
Introduction 42
Model implementation and intuition through posteriors 43
Burial dates 43
Age at death 44
Age of Motherhood 45
Age of Fatherhood 45
Sibling Model 46
Avuncular model 47
Cousin model 49
Additional models, inconsistent with genetic data 50
Model comparison through marginal likelihoods 51
Autosomal relatedness 54
Mitochondrial relatedness 54
Unrelated case 54
Cryptic background relatedness 55
Updating the likelihoods given mitochondrial and autosomal evidence 56
Code availability 57
Supplementary Note 4: The formation of the Hallstatt Gene pool **58**
General population genetic affinities 58
Supervised ADMIXTURE modelling 61
Pairwise qpWave testing 62
qpAdm modelling using distal sources 63
Supplementary Note 5: Population genetic changes after the Hallstatt period **67**
The end of the Hallstatt gene pool in southern Germany 67
Modelling present-day Germany 74
The end of the Hallstatt Gene pool in the Czech Republic 75
Supplementary References **77**

Supplementary Note 1: Site descriptions

The Asperg-Grafenbühl burial mound

The Asperg-Grafenbühl burial mound is located on a flat ridge 650 m south-east of the Celtic centre of power on the Hohenasperg. The tumulus has a diameter of approximately 40 m and a still preserved height of about 2.2 m (with the reconstructed height being 4 m). Due to viticulture during the 16th century and the later establishment of an orchard, the mound had already been severely disturbed. Today, the area is located in the middle of the town of Asperg and has been almost completely built over.

After the State Office for Cultural Heritage (Landesamt für Denkmalpflege) had been notified following the discovery of a skeleton and an accompanying arrowhead, Hartwig Zürn investigated the tumulus in 1964/65 and uncovered the central grave chamber, measuring 4.5 by 4.5 m. It had been dug about 0.8 m into the existing soil, but had already been extensively looted in antiquity. In the south-western corner of the chamber, the disarticulated skeleton of a mature male was found.

The deceased's personal equipment included a gold-plated belt hook and two gold-covered bronze fibulae. In addition, the remains of a sumptuous textile adorned with gold lamé were documented as scattered within the chamber. Further burial goods included the remains of a four-wheeled wagon, two lion feet of a tripod (probably bronze) imported from Greece (Supp. Fig. 1.1), parts of two bronze cauldrons and a drinking horn, as well as fittings and inlays of amber, bone and ivory from a seating device and other pieces of Mediterranean furniture. The Phoenician-Syrian ivory disc belonging to a mirror or fan handle and a rattle with ivory handle are also noteworthy.

Despite the looting, it appears that the central grave of the Asperg-Grafenbühl burial mound had once been the most opulent grave in the vicinity of the Hohenasperg (Supp. Fig. 1.2).

In the area of the mound, 33 secondary burials, including several double burials, were uncovered, dating as late as the Early La Tène period. All of them represent relatively simply furnished inhumations, some of which were heavily disturbed.

Bibliography:

Zürn, H., Hallstattforschungen in Nordwürttemberg. Veröffentlichungen des staatlichen Amtes für Denkmalpflege Stuttgart Reihe A 16 (Stuttgart 1970) 7-51.

Zürn, H., Hallstattzeitliche Grabfunde in Württemberg und Hohenzollern. Forschungen und Berichte zur Vor- und Frühgeschichte in Baden-Württemberg 25 (Stuttgart 1987), 91-94.

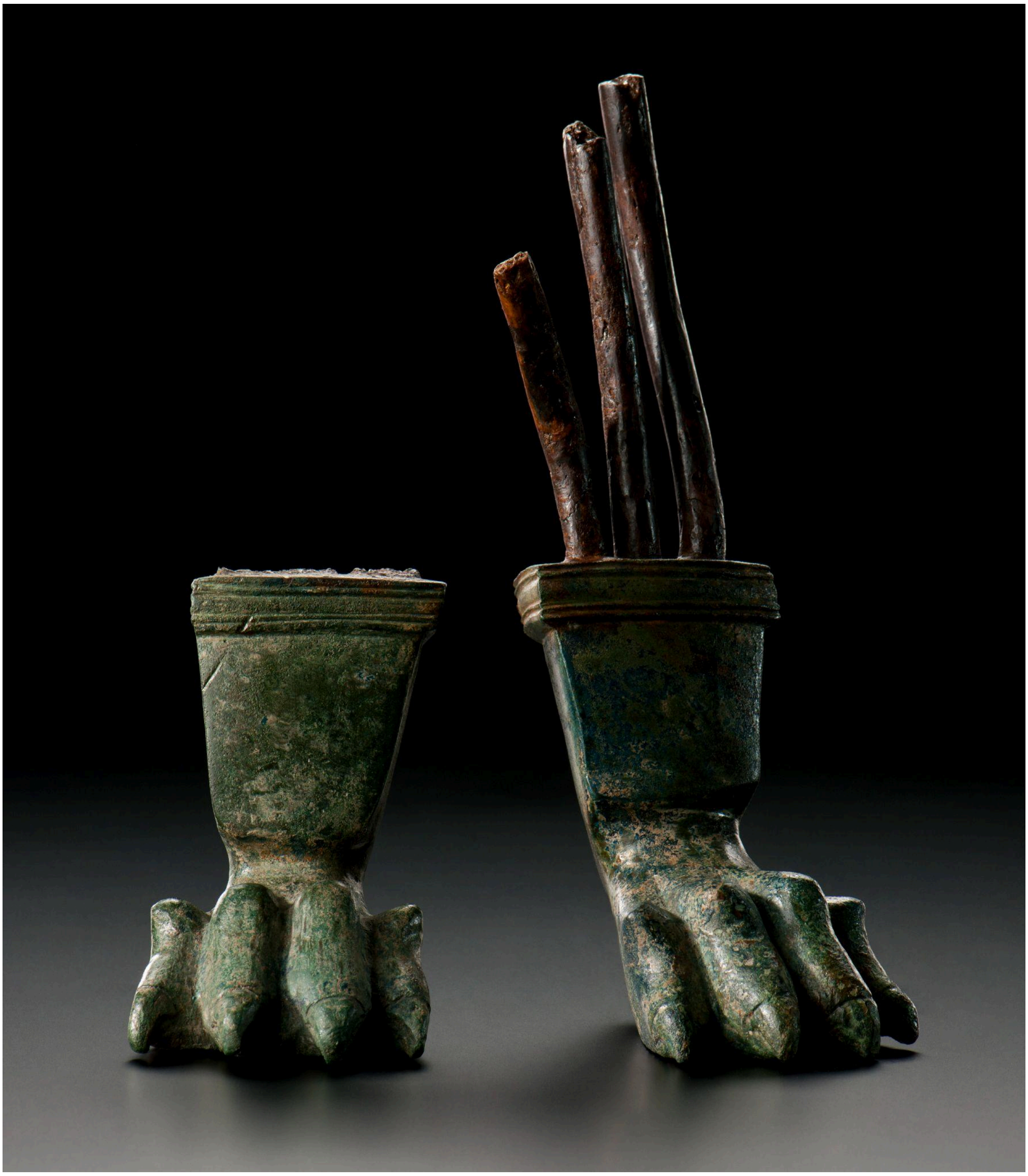

Supplementary Figure 1.1. Two lion's paws cast in bronze, probably originally from a tripod (Landesmuseum Württemberg, Hendrik Zwietasch).

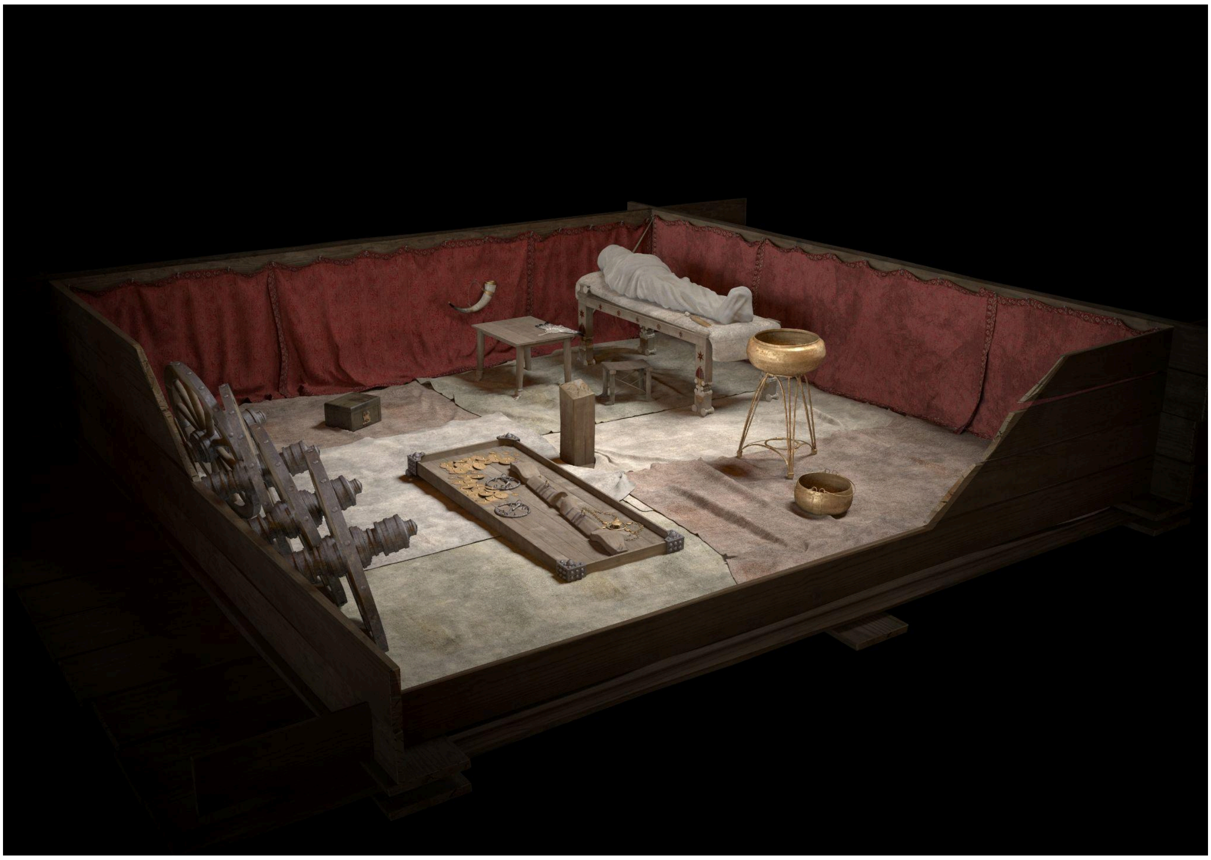

Supplementary Figure 1.2. 3D reconstruction of the central burial chamber from Asperg-Grafenbühl (Landesmuseum Württemberg, FaberCourtial; Thomas Hoppe [scientific reconstruction]).

The Römerhügel burial mound

In 1877, during the installation of a water tower and tank in Ludwigsburg, a burial was discovered in the so-called “Römerhügel”, also known as "Belle Remise". The name “Römerhügel” (Roman Hill) probably derives from the assumption that the elevation of the burial mound had originally been the site of a Roman watchtower. Prior to its destruction, the tumulus had been an imposing structure with a height of 6 m and a diameter of 60 m. Another smaller mound in the vicinity probably also belongs to this necropolis, but has not been investigated so far.

A square burial chamber of 3.5 by 3.5 m was discovered in 1877 at a depth of 5.5 m covered by a stone package. At the western wall of the chamber, the skeleton of a mature man was found (grave 1). A gold neck ring was excavated close to the skull and a ceremonial dagger with amber inlays on the handle (Supp. Fig. 1.3) as well as a whetstone in the pelvic area. The grave also contained the remains of a four-wheeled wagon including elements of horse harness, a strip of gold foil (probably of a drinking horn) and four bronze vessels comprising a basin, a cauldron, a ribbed bucket and a bowl. The equipment indicates a high social rank of the deceased.

A second wooden burial chamber (grave 2), probably the central grave, was discovered 3 m north of grave 1. This burial had been dug 1.2 m into the ground. However, investigations were limited to its peripheral area and revealed a decorated gold metal strip, the handle of a dagger and several amber fragments, which could possibly be interpreted as fittings from a seating device or another piece of furniture (Supp. Fig. 1.4).

Extension works to the water tank in 1926 and 1927 led to the discovery of at least 14 secondary burials dug into the Römerhügel – all of them inhumations, but either with much more modest burial goods or even without any. Bronze and iron fibulae, bronze neck, arm and ankle rings, pins and an iron spearhead are worth mentioning. The latest secondary burial with burial goods, containing a pottery bottle, already belongs to the Early La Tène period.

The Römerhügel is located about 3.8 km south-east of the Hallstatt period centre of power at the Hohenasperg.

Bibliography:

Zürn, H., Hallstattzeitliche Grabfunde in Württemberg und Hohenzollern. Forschungen und Berichte zur Vor- und Frühgeschichte in Baden-Württemberg 25 (Stuttgart 1987), 98-101.

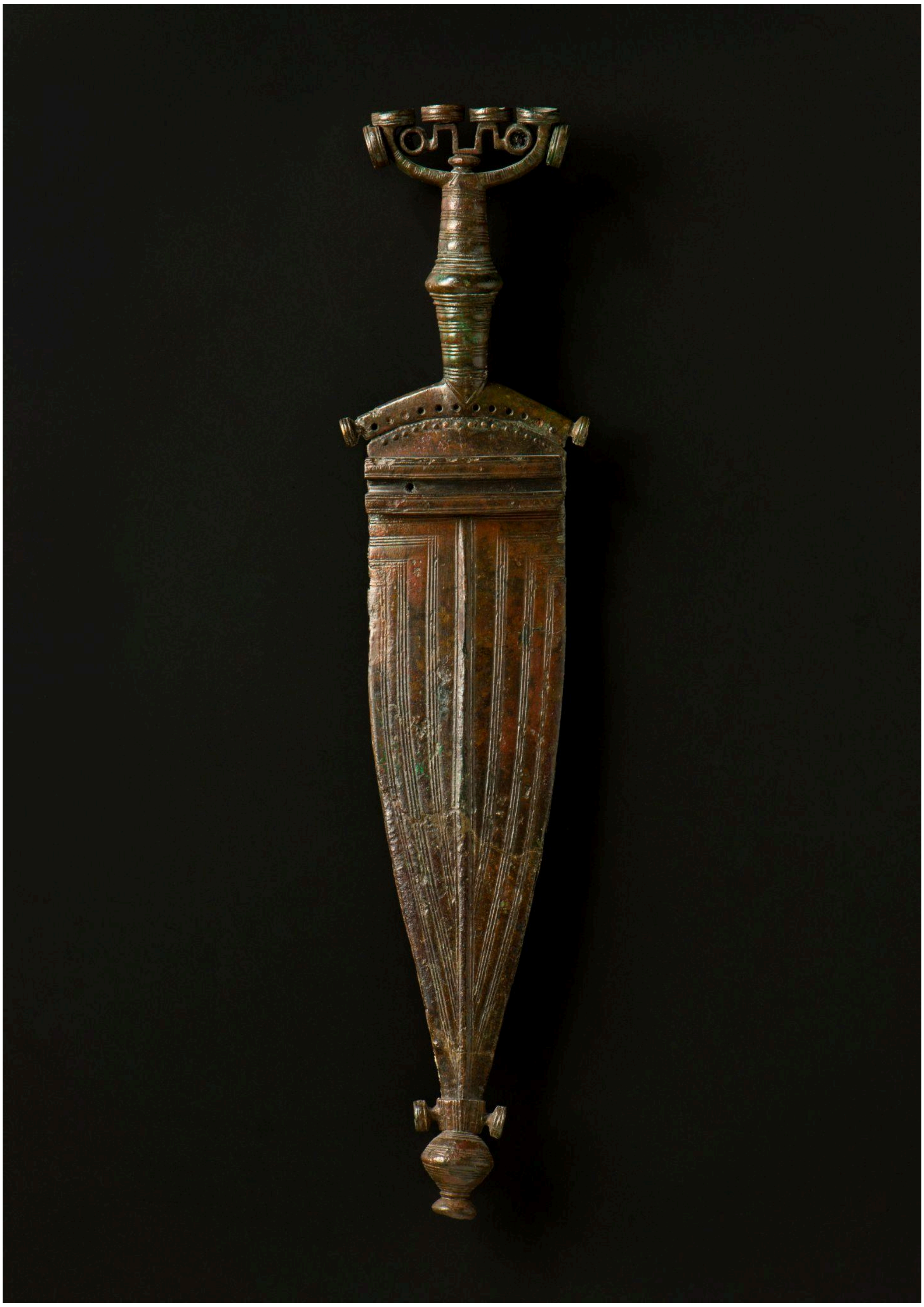

Supplementary Figure 1.3. Intricately decorated dagger with amber inlays on its handle (Landesmuseum Württemberg, Hendrik Zwietasch).

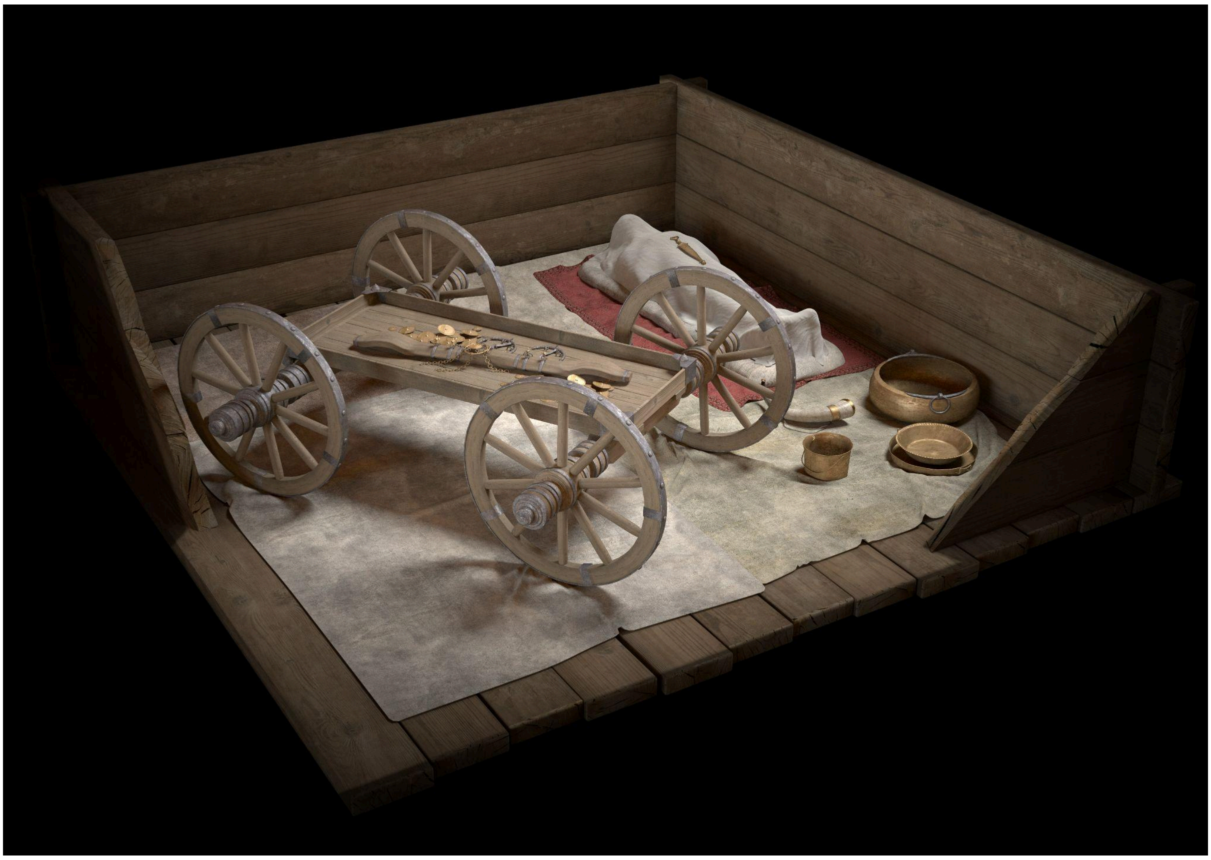

Supplementary Figure 1.4. 3D reconstruction of the burial chamber (tomb 1) from Ludwigsburg-Römerhügel (Landesmuseum Württemberg, FaberCourtial; Thomas Hoppe [scientific reconstruction]).

The Early Celtic centre of power at the Heuneburg

The Heuneburg is widely visible above the river Danube between the villages of Herbertingen-Hundersingen (district of Sigmaringen) and Ertingen-Binzwangen (district of Biberach), situated at the transition from the southern edge of the Swabian Alb to the south-eastern bordering Alpine foothills in a favourable geographic location (Supp. Fig. 1.5 & 1.6). The hilltop plateau, about 3 ha in size and 600 m above sea level, towers over the Danube valley by about 60 m.

The Heuneburg plateau has already been settled during the Middle Bronze Age and Urnfield Period (1600-1100 BCE). Its peak period, however, dates into the Hallstatt period (620-450 BCE), for which alone 14 construction stages have been attested. Thanks to systematic excavations and research by the University of Tübingen and the State Office for Cultural Heritage (Landesamt für Denkmalpflege) since 1950, the Heuneburg is considered one of the best-researched centres of power of the Early Celtic period. Around 620 BCE, enclosed farmsteads were built on the hilltop plateau, which were protected by a surrounding timber box rampart in log construction. In addition, a fortification ditch ran along the foot of the slope.

Around 600 BCE, the Heuneburg plateau was completely restructured. The hilltop was enclosed by a fortification inspired by the Mediterranean or the Middle East with a stone foundation and a superstructure of air-dried mud bricks and a battlement. The western side of the wall comprised a series of bastion-like projecting wall towers of the same construction. The interior structure of the plateau consisted of densely built rows of houses along an angular network of paths. This type of building indicates an early form of urban life. Adjoining the western side of the plateau, the loosely built lower town, about 1.5 ha in size, was also protected by a moat, rampart, palisade and a monumental gate constructed of a stone foundation and mud-brick walls. However, the hilltop plateau and lower town merely formed the core of a much larger settlement, which extended over a total area of more than 100 ha during the first half of the 6th century BCE. This outer settlement was in turn fortified by rampart-ditch systems and partly subdivided into quarters with farmsteads set up next to each other. In contrast to the plateau, the outer settlement also included large representative buildings with a floor area of up to 320 m². It can be assumed that during the first half of the 6th century BCE a total of around 4,000-5,000 people lived on the hilltop plateau, in the lower town and in the outer settlement.

Around 530 BCE, a fire led to the abandonment of the outer settlement and the construction of four burial mounds about 250 m northwest of the lower town. On the hilltop plateau, the mud-brick wall was demolished and the construction returned to a local method in the form of a wall made of wood, stones and earth. Enclosed courtyards were once again built in the interior. Moreover, large representational buildings with a floor area of more than 400 m² were now built on the plateau. The area of the lower town remained in use after the devastating fire, and settlement activities even intensified. The stone gate was replaced by simpler wooden structures in the later phases. Most of the imports, which are mainly fragments of Greek pottery and amphorae from Massalia and southern Italy, date from the period after the destruction of the mud-brick wall.

The end of the Heuneburg and the Iron Age settlement was sealed around 450 BCE by another catastrophic fire. Of particular interest here are the almost 500 human bones of at least 7-8 individuals found in a fortification trench below the plateau on a terrace to the east above the Danube valley, some of which show traces of violence. In addition, four arrowheads were documented amongst the human bones. These findings indicate warlike activities at the end of the Early Celtic Heuneburg period.

More recent investigations show that the surrounding areas with further hilltop fortifications, rural settlements, burial sites, traffic routes and the ritual site at the Alte Burg also belonged to the complex settlement system of the Heuneburg.

Against this background, the long-neglected mention of the polis Pyrene on the upper course of the Danube by the Greek historian Herodotus of Halikarnassos (484-425 BCE) appears in a new light. Around the middle of the 5th century BCE, he wrote in his famous work Histories (II, 33): "*For the Istros* [the Greek name of the Danube] *flows from the land of the Celts and the city of Pyrene through the very middle of Europe*" (English translation by A. D. Godley 1920: Cambridge, Harvard University Press; with amendments). Since inscriptions are missing, it remains impossible for the time being to prove that the Heuneburg was indeed this Early Celtic town. However, in view of the dimensions, structure and importance of the Heuneburg, which are now evident, as well as the known spectrum of finds, there can be little doubt that both the Etruscan cities of central Italy and the Greek colonies of southern France had knowledge of this Celtic centre of power on the upper Danube.

Bibliography:

Krausse, D., Fernández-Götz, M., Hansen, L. and Kretschmer, I. *The Heuneburg and the Early Iron Age Princely Seats. First Towns North of the Alps*. Budapest: Archaeolingua Publishing House (2016).

Krausse D., Hansen, L. and Tarpini, R.. Earliest Town North of the Alps. New Excavations and Research in the Heuneburg Region. In: Zamboni, L., Fernández-Götz, M. and Metzner-Nebelsick, C. (eds.), Crossing the Alps. Early Urbanism between Northern Italy and Central Europe (900-400 BC) (Sidestonepress 2020) 299-317.

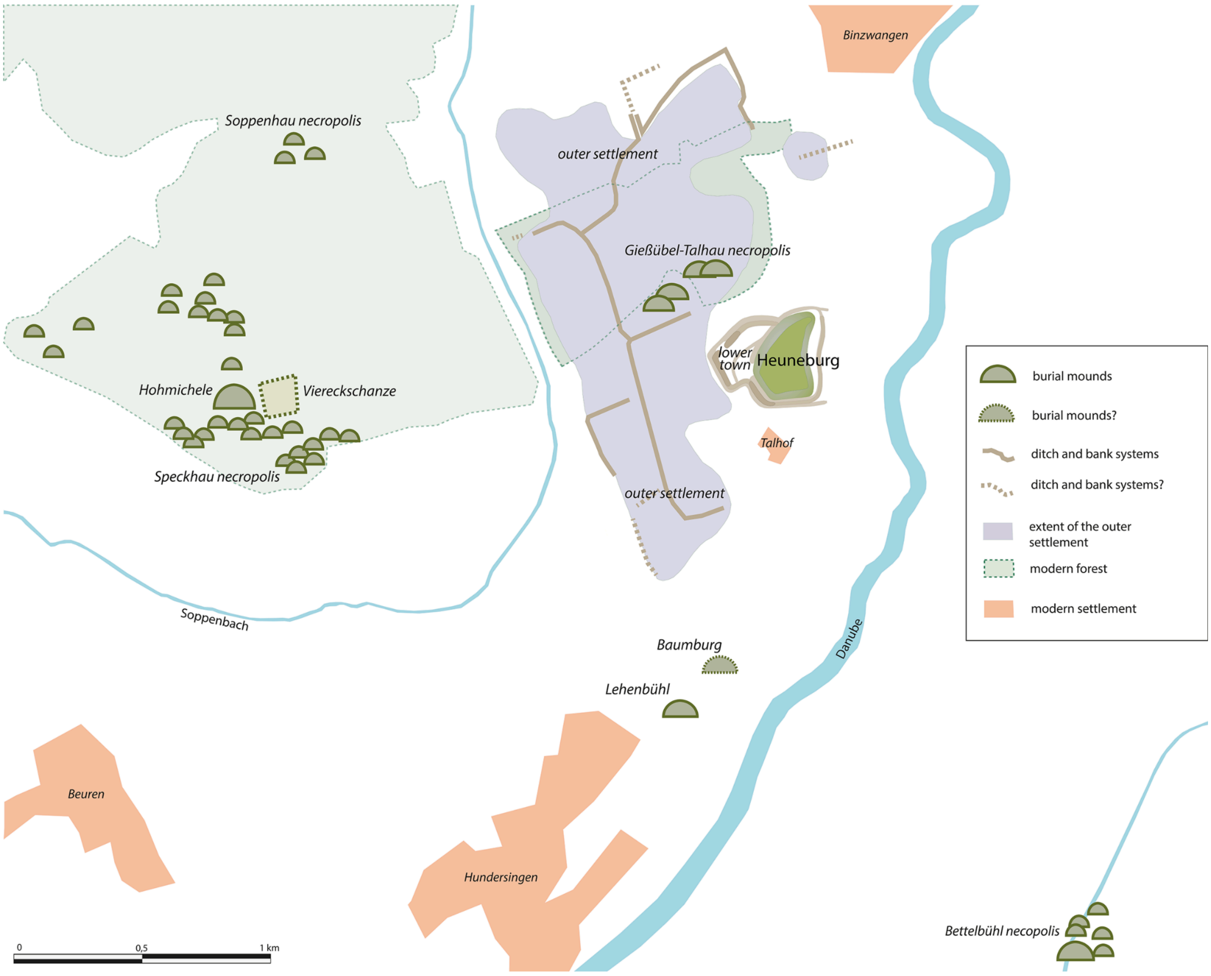

Supplementary Figure 1.5. Map of the Heuneburg with the hilltop plateau, the lower town, the outer settlement and surrounding cemeteries (Landesamt für Denkmalpflege im Regierungspräsidium Stuttgart/I. Kretschmer, L. Hansen).

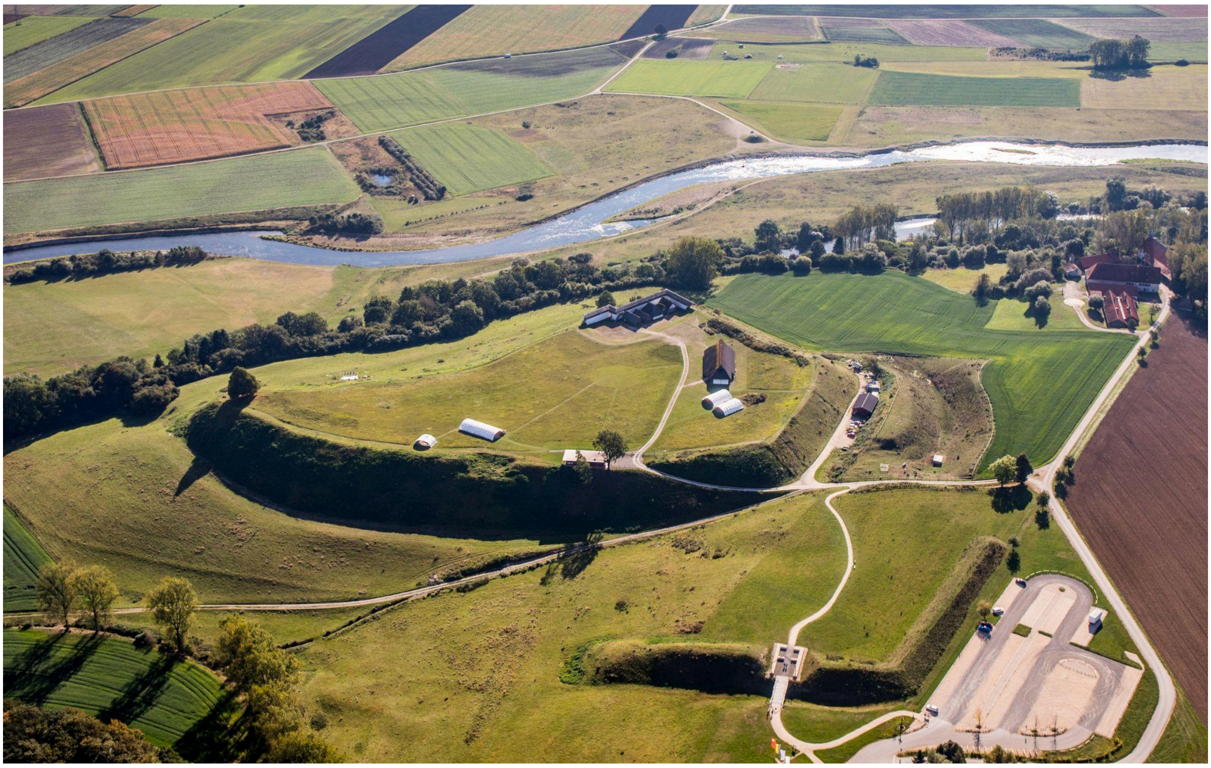

Supplementary Figure 1.6. Aerial view of the Heuneburg on the Danube with today's open-air museum (Landesamt für Denkmalpflege im Regierungspräsidium Stuttgart/A. Drescher).

Landscaping at the Alte Burg

About 9 km north-northwest of the Heuneburg, the Alte Burg is located on the southern edge of the Swabian Alb at an altitude of about 700 m above sea level near Langenenslingen (district of Biberach). The interior area, which is situated about 100 m above the bottom of the valley, extends over 2 ha.

The first documented three-day excavation took place in 1894 in the area of a stone mound on the plateau, about 1.8 m high. Beneath it, a shaft cut into the rock was uncovered, in which six human skeletons are said to have rested on top of each other. Unfortunately, the whereabouts of these finds is unclear. In 2006-2008, the Alte Burg underwent further modern research and the shaft, which was about 5 m deep, was excavated again (F1-3, 5-6). The excavation revealed 49 fragments of human bones, which could be dated to the 4th-3rd century BCE using the radiocarbon method.

Research at the Alte Burg continued between 2014 and 2020. The excavations have demonstrated that the entire mountain spur was extensively reshaped during the Hallstatt period (Supp. Fig. 1.7). To create a plane surface, the plateau, which is about 340 m long and between 55 and 65 m wide, was levelled in places. Furthermore, the edges of the slope were widened at great expense, resulting in today's regular tongue-shaped outline. To achieve this, numerous massive stone struts, built as drystone walls, were constructed at right angles to the edge of the plateau and the spaces between them were filled with stones and clay. In the north-east, the Alte Burg was originally protected by a gigantic, 13 m thick and at least 10 m high double-shell wall (D), which was preceded by a deep ditch (C) and two further ramparts (A-B). On the inner side of this massive wall, a 5.8 m thick, over 30 m long drystone wall was built at a right angle (J). In addition, a terrace (E-F) was built about halfway up the steep slopes on each of the two long sides. An ancient path leading up the steep slope from the north-east was probably already in use by the Hallstatt period, as pottery finds indicate (H).

Towards the valley, the entire imposing ridge is enclosed by a fortification about 1 km long (G). Excavations have shown that it was a simple rampart with a large inner ditch more than 4.5 m deep. It became also evident that the slope between the terrace and the ditch had been artificially steepened considerably.

The finds known so far from the Alte Burg date mainly to the Hallstatt period. In addition, there are a few Early La Tène metal finds from the area around the shaft, as well as the human bones dating to the 4th-3rd century BCE.

There is much to suggest that the Alte Burg was a place of gathering and ritual. This is indicated by the shaft (Supp. Fig. 1.8) with its deposited human skeletons, the construction of the monumental walls, ditches and ramparts, as well as the complex levelling work, which obviously served only one purpose: to create a representative structure with a plateau that was as level and evenly tongue-shaped as possible. It is noticeable that the extensive excavations have so far revealed no evidence of building structures on the plateau. In addition, there is no provision for water supply on this spur of the Alb.

The shape and dimensions of the plateau – elongated with a rounded narrow side – have no equivalent amongst the known Hallstatt period hillforts in southern Germany. With regard to its shape, however, the Alte Burg is vaguely reminiscent of the layout of ancient tracks for horse or chariot races. A ledge dividing the hilltop lengthwise (I) could be interpreted as a "spina" which subdivided the trackway along its longitudinal axis. Excavations carried out at the south-western end of the plateau (S37) have revealed that the ledge is not present there and, thus, there is effectively an opening.

Numerous finds, such as nine wagon graves, the equestrian equipment in tumulus 4 of the Bettelbühl necropolis or a recently discovered bronze equestrian figure from Unlingen (district of Biberach) near Mount Bussen, impressively demonstrate the importance of elite riding and carriage driving in the Heuneburg region during the Hallstatt period. The fact that the early Celts must have been familiar with horse and chariot races is also attested to by pictorial representations, e.g. from the so-called "situla art".

Bibliography:

Krausse D., Hansen, L. and Tarpini, R. Earliest Town North of the Alps. New Excavations and Research in the Heuneburg Region. In: Zamboni, L., Fernández-Götz, M. and Metzner-Nebelsick, C. (eds.), Crossing the Alps. Early Urbanism between Northern Italy and Central Europe (900-400 BC) (Sidestonepress 2020) 299-317.

Hansen, L., Krausse, D. and Tarpini, R. Die Alte Burg in frühkeltischer Zeit - ein prähistorisches Hippodrom? Archäologie in Deutschland 2021/3, 30-33.

Hansen L, Krausse, D. Reiten und Fahren an der Heuneburg. In: B. Nessel/L. Nebelsick (Hrsg.), Quod erat demonstrandum. Vorgeschichtliche Studien Christopher F. E. Pare gewidmet – Studies in Prehistory dedicated to Christopher F. E. Pare. Universitätsforschungen zur prähistorischen Archäologie 380 (Bonn 2022) 177-188.

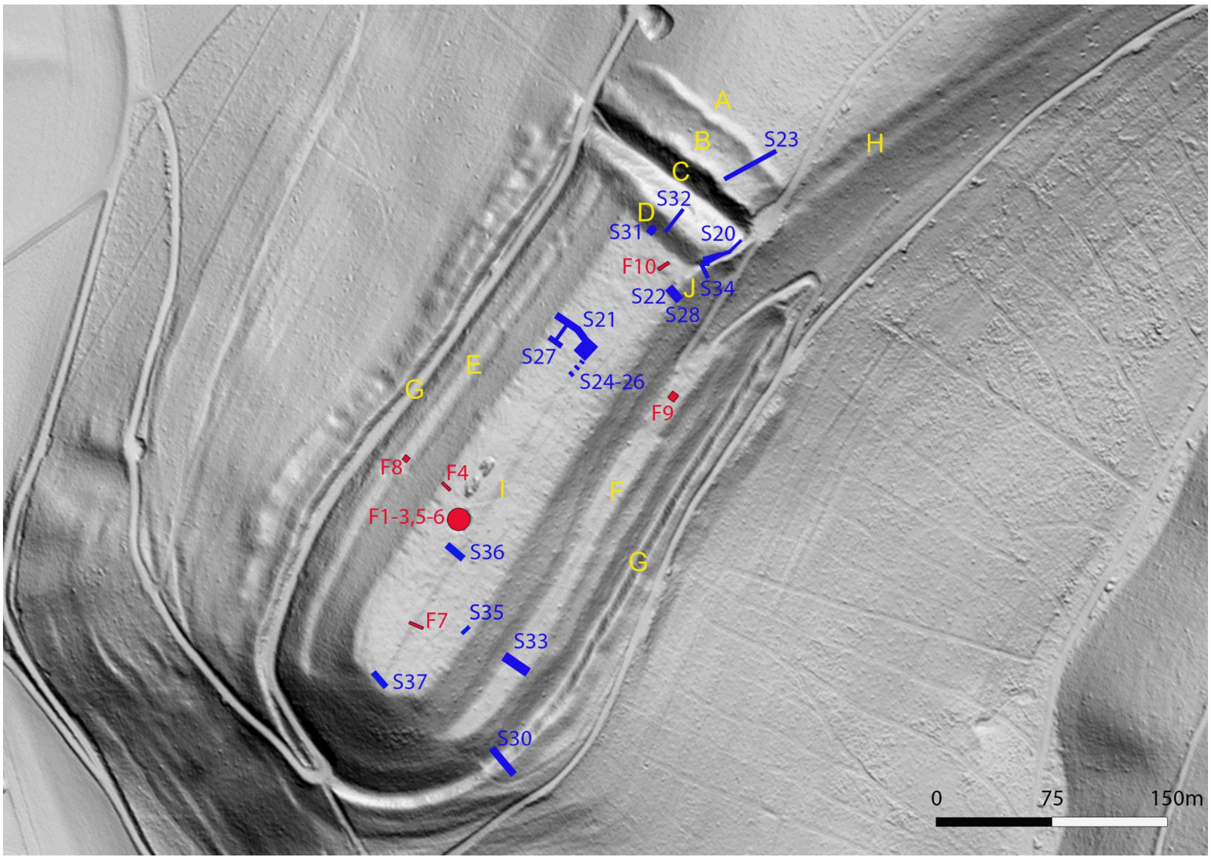

Supplementary Figure 1.7. Digital terrain model of the Alte Burg showing the excavation sections from 2006-2007 (red) and 2014-2020 (blue) and the most important elements of the structure (Landesamt für Denkmalpflege im Regierungspräsidium Stuttgart/R. Hartmayer, L. Hansen, map basis LGL).

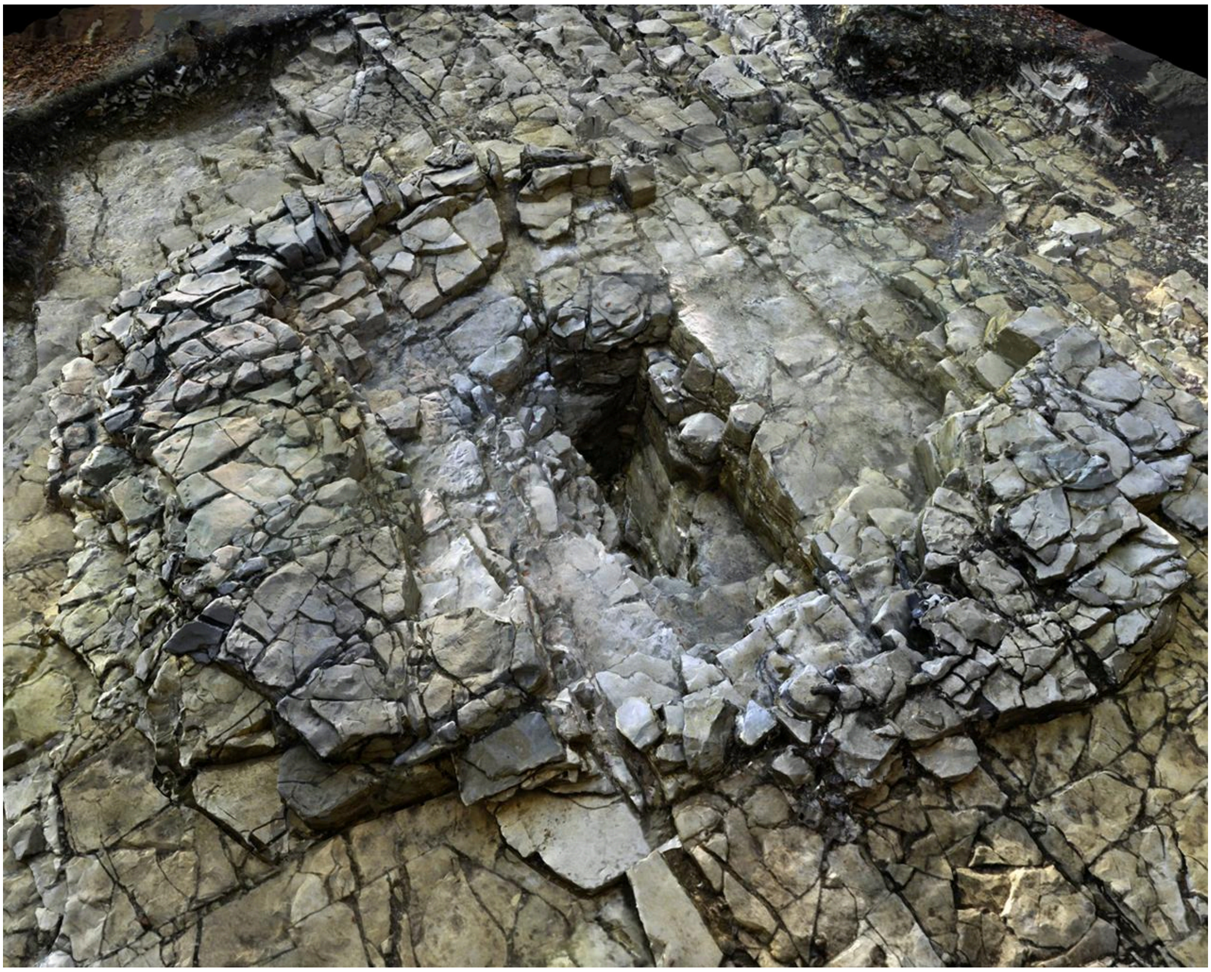

Supplementary Figure 1.8. View into the Alte Burg shaft enclosed by a platform of natural rock (Landesamt für Denkmalpflege im Regierungspräsidium Stuttgart/ArcTron 3D GmbH).

The Eberdingen-Hochdorf burial mound

The Late Hallstatt burial mound of Hochdorf is located north-west of Stuttgart in a fertile loess landscape. The monumental tumulus was systematically excavated in 1977-1979; the excavations proved to be a defining moment in archaeology. The mound had a diameter of 60 m. Its original height can no longer be precisely determined, as it had already been flattened by agriculture and erosion, but can be estimated at about 6 m. At the centre of the mound, a burial shaft 11 by 11 m in size, and 2.5 m deep was found. The actual burial chamber measured 4.7 by 4.7 m and had been protected by its builders with an elaborate vault-like construction of timber and stone. The chamber (Supp. Fig. 1.11) contained the luxurious burial goods for the deceased, a tall, strongly built man who had died at the age of about 50 (HOC001). His body was positioned extended and supine on an elegantly shaped, richly decorated bronze sofa (length 2.75 m); an object that is without parallels to this day. The deceased wore, amongst other things, pointed shoes decorated with gold sheet, a bronze belt also covered with gold sheet, a gold neck ring and a dagger, the bronze scabbard of which had been covered with gold sheet especially for the burial (Supp. Fig. 1.9). Above the bronze sofa, nine large drinking horns decorated with gold sheet hung on the chamber walls. A dining service for nine persons, placed on a four-wheeled wagon plated with iron, accompanied the horns. Four tools suitable for killing large animals such as cattle or deer had also been placed atop the wagon, and can be interpreted as implements intended for ritual animal sacrifices. The additional presence of a bowl made of gold sheet, which was likely for drinking or libations, also points to a religious function for these tools. The golden bowl was found at the bottom of an oversized bronze cauldron, which had a capacity of about 500 litres (Supp. Fig. 10). At the time of burial, it had been filled two thirds of the way with honey mead. Three cast bronze lion figures adorn the rim of the cauldron, which has been the product of a Greek workshop, transported to south-western Germany either across the Alps or via the Rhone and its tributaries. A quiver filled with arrows must be interpreted as a hunting weapon. The extensive remains of high quality textiles were also preserved throughout the grave.

Based on the burial goods, the central grave can be dated with some precision. The deceased was likely buried around 530/520 BCE. Considering he had died at about 50 years of age, he would have been born around 580/570 BCE. His height has been calculated at 1.80-1.84 m. This makes him the tallest Iron Age individual in southern Germany to date. The skeleton is very robust and shows strong muscle attachments, indicating a very strong and athletic physique.

His burial goods, and the extraordinarily elaborate architecture of the grave, indicate that the deceased belonged to the highest levels of elite society – probably, indeed, a royal ruler, whose role encompassed both political and religious powers.

In addition to the central burial shaft, three more graves were found in the burial mound during the excavations. Originally, the huge burial mound may have contained many more secondary burials, which were, however, lost due erosion over the past 2,500 years.

One secondary burial (HOC003), discovered at the south-western edge of the mound, had been constructed at about the same time as the central grave. It contained the skeleton of an adult male about 40-50 years of age. The only burial goods found with the deceased

were two bronze serpent fibulae, an undecorated bronze belt-plaque, and a simple iron knife. The man was undoubtedly a contemporary of the “prince” buried in the central tomb, and had been born a little later than the latter, around 570 BCE. He is genetically related to the adult woman from grave 95 of the Magdalenenberg (MBG009) in a third degree relationship. Next to the male skeleton, a ceramic vessel containing cremated bone material was found. These cremated remains likely belonged to rather a young and male individual, although their small amount precludes a more reliable determination.

Burial HOC002, which was found immediately west of the central burial shaft and had probably also been created at about the same time as the central grave, belonged to a late-mature male who has died at about 50-60 years of age, and thus had been born around 590/580 BCE. His grave was poorly furnished. The only grave goods found were two bronze serpent fibulae and an iron razor.

Stratigraphically and typologically younger is the secondary burial HOC004, which was inserted into the stone package at the northern edge of the burial mound at a later stage. The burial belongs to a young man who probably died between 520 and 500 BCE at about 20-30 years of age. Amongst the burial goods found were two large iron spearheads, a closed circular section bronze neck ring and two cast bronze drum brooches.

As with the Asperg-Grafenbühl and Ludwigsburg-Römerhügel tumuli, Hochdorf belongs to the cultural environment of the early Celtic centre of power of Hohenasperg. Not far from the burial mound, the remains of an early Celtic settlement were uncovered in the 1990s, which is, however, younger and belongs to the 5th-4th century BCE.

Bibliography:

Krausse, D. Hochdorf III. Das Trink- und Speiseservice aus dem späthallstattzeitlichen Fürstengrab von Eberdingen-Hochdorf (Kr. Ludwigsburg). Forschungen und Berichte zur Vor- und Frühgeschichte in Baden-WürttembergForschungen und Berichte zur Vor- und Frühgeschichte in Baden-Württemberg 64 (Stuttgart 1996).

Hansen, L., Hochdorf VIII. Die Goldfunde und Trachtbeigaben des späthallstattzeitlichen Fürstengrabes von Eberdingen-Hochdorf (Kr. Ludwigsburg). Forschungen und Berichte zur Vor- und Frühgeschichte in Baden-Württemberg 118 (Stuttgart 2010).

Biel, J., Keefer, E. (eds.), Hochdorf X. Das bronzene Sitzmöbel aus dem Fürstengrab von Eberdingen-Hochdorf (Kr. Ludwigsburg). Forschungen und Berichte zur Archäologie in Baden-Württemberg 20 (Wiesbaden 2021).

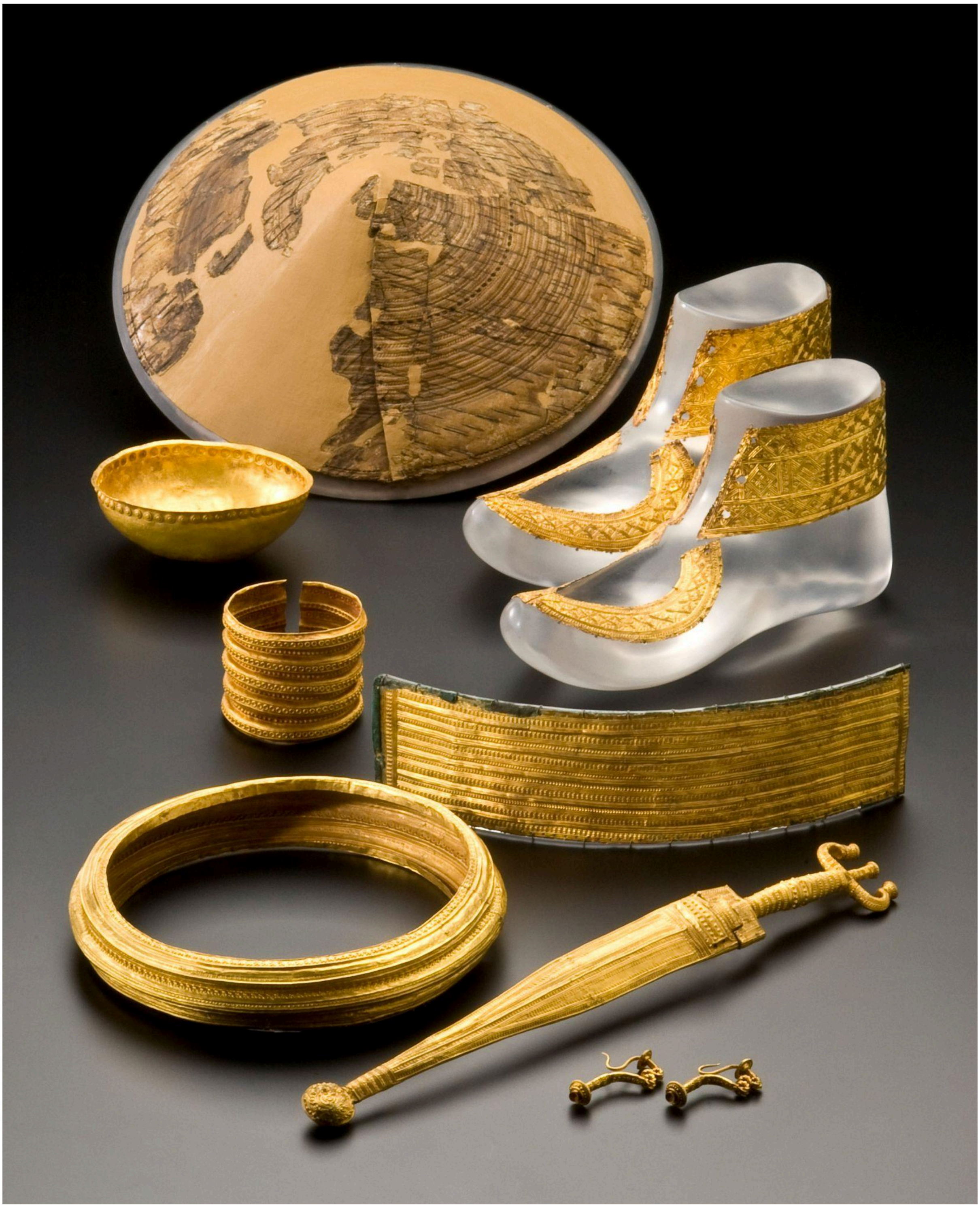

Supplementary Figure 1.9. Rich gold finds and a hat made from birch bark from the central tomb at Eberdingen-Hochdorf (Landesmuseum Württemberg, P. Frankenstein / H. Zwietasch).

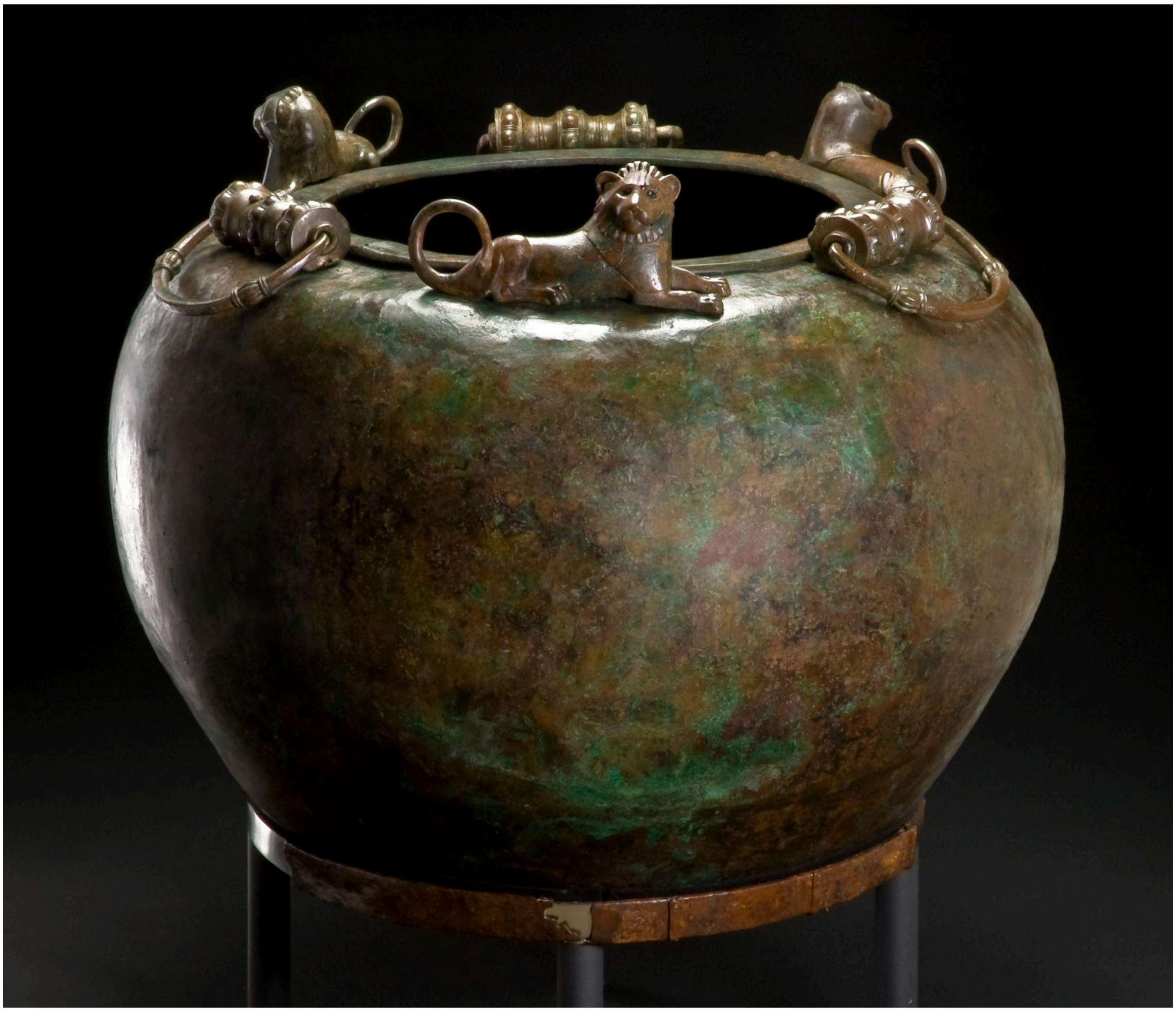

Supplementary Figure 1.10. Bronze cauldron with lion decorations with a capacity of about 500 litres (Landesmuseum Württemberg, P. Frankenstein / H. Zwietasch).

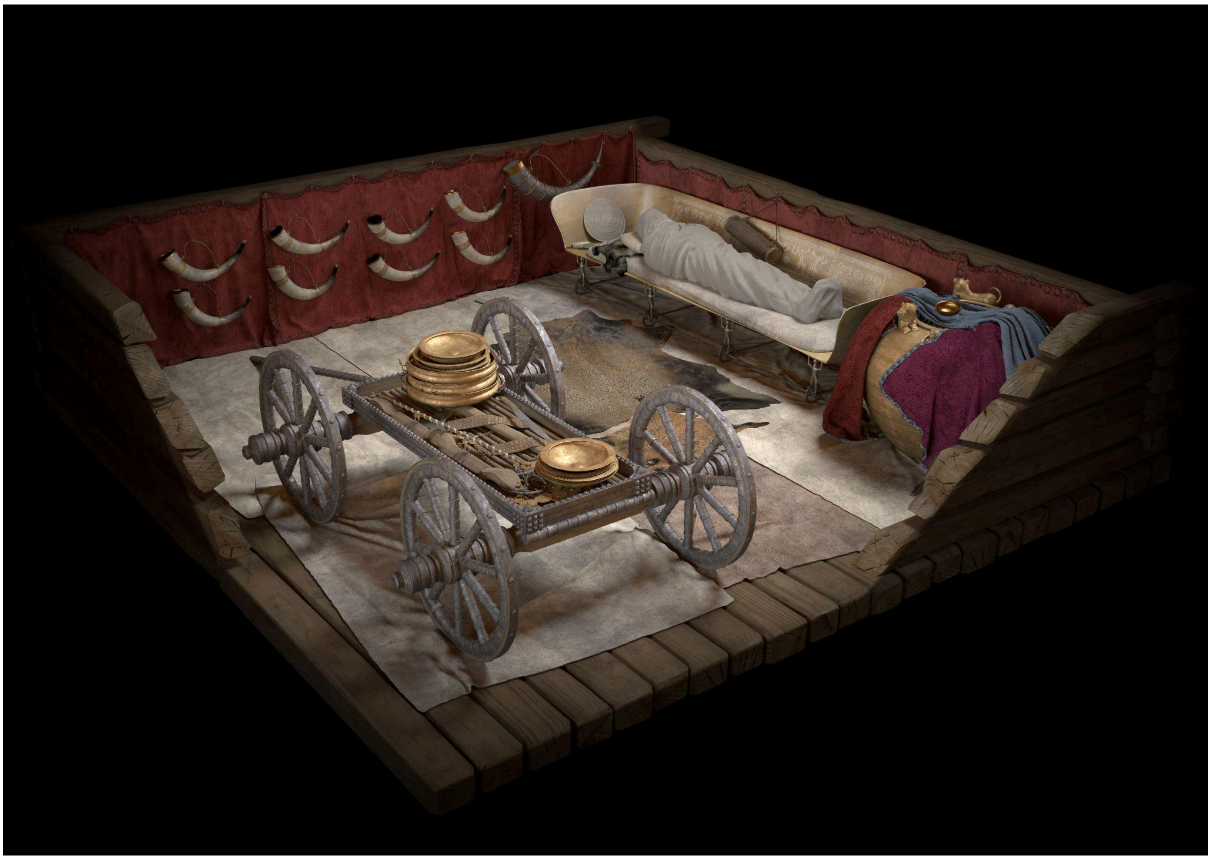

Supplementary Figure 1.11. 3D reconstruction of the burial chamber from Eberdingen-Hochdorf (Landesmuseum Württemberg, FaberCourtial; Thomas Hoppe [scientific reconstruction]).

The Magdalenenberg burial mound near Villingen

With a diameter of 102 m and an original height of about 8 m, the “Magdalenenbergle” is – despite its diminutive name – one of the largest burial mounds in Central Europe (Supp. Fig. 1.12). Ernst Wagner and Karl Schumacher as well as the town of Villingen carried out first excavations in 1887 and 1890 and discovered a looted burial chamber in the centre of the mound. In 1970-1973, Konrad Spindler completely excavated the mound and the central burial chamber of about 8 x 6 m with its preserved timbers (Supp. Fig. 1.13). Dendrochronological dating of the oak timbers indicated a felling date of 616 BCE, at the beginning of Ha D1. Although a massive stone package protected the central chamber, it was probably looted soon after the deceased had been interred. Only remains of a wagon and horse harness, fragments of wooden furniture, a gold-plated bracelet, small bronze objects and fragments, as well as textile and leather remains survived of the originally very rich burial goods assemblage. The skeletal remains found were determined to be those of a 30-40 year old man. Wooden structures and the placement of posts within the mound fill appear to be part either of the central burial or of the concept behind the placement of secondary burials, but their exact function remains unclear to this day.

Inside the enormous mound, a further 126 graves have been discovered in a tangential-circular arrangement around the central grave. Predominantly inhumations of single adults, partly in wooden coffins, they also include eight cremations, one triple burial and eleven double burials. They thus represent the largest cemetery of secondary burials of the Western Hallstatt area. It can be assumed that their number had initially been even more extensive, but erosion and previous excavations eradicated some of the graves.

The rich assemblages of items of dress and grave goods in the secondary burials suggest that the deceased belonged to the upper and middle social strata; they date to Ha D1 or to the beginning of Ha D2. Most of the objects find good parallels in the northern Alpine Western Hallstatt area, but some also attest to long-distance relationships with the south-eastern Alps and the Mediterranean.

The findings suggest that over 2-3 generations a community buried its deceased in a “standardised” arrangement with reference to the central grave; four chronological horizons have been identified. Such “kinship burials”, particularly on this scale, are unusual for the Western Hallstatt area. In 2009-2011, the secondary burials were the basis for social archaeological studies on the Late Hallstatt period, focusing on the question of individual mobility and the integration of foreign people.

The Magdalenenberg was backfilled after the excavation, the finds and the burial chamber are presented in the Franziskanermuseum Villingen.

Bibliography:

Spindler, K. Der Magdalenenberg bei Villingen im Schwarzwald: Bilanz nach dreißig Jahren. in Parerga praehistorica. Jubiläumsschrift zur prähistorischen Archäologie – 15 Jahre UPA (ed. Hänsel, B.) vol. 100 135–160 (2004).

Mötsch, A. Forschungen am Magdalenenberg bei Villingen. In: Neue Forschungen zum Magdalenenberg. Archäologische Informationen aus Baden-Württemberg (ed. Krausse, D.) vol. 77 14-27 (2017).

Koch, J. K. Vom Schwarzwald zum Mittelmeer und zurück. Fremde Objekte, Individuen und kulturelle Kontakte in der hallstattzeitlichen Gemeinschaft vom Magdalenenbergle. In: Neue Forschungen zum Magdalenenberg. Archäologische Informationen aus Baden-Württemberg (ed. Krausse, D.) vol. 77 38–52 (2017).

Huth, Ch. Der hallstattzeitliche Grabhügel auf dem Magdalenenberg. In: Archäologische Erlebnisorte zwischen Odenwald und Bodensee. Hrsg. zum 50jährigen Bestehen des Förderkreises Archäologie in Baden e.V. von G. Seitz (2018) 94-96.

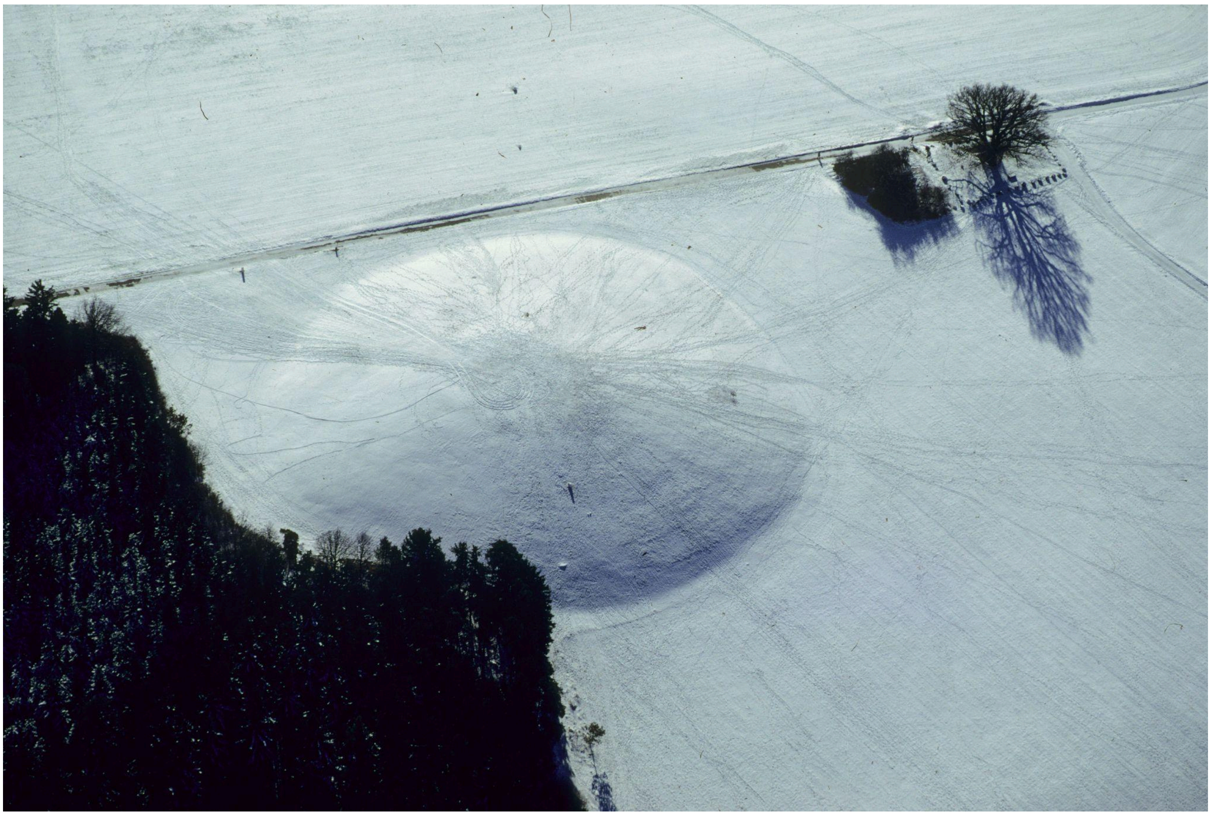

Supplementary Figure 1.12. Aerial view of the Magdalenenbergle burial mound near Villingen (Landesamt für Denkmalpflege im Regierungspräsidium Stuttgart/Foto: Otto Braasch, L7916-012-01_5380-01).

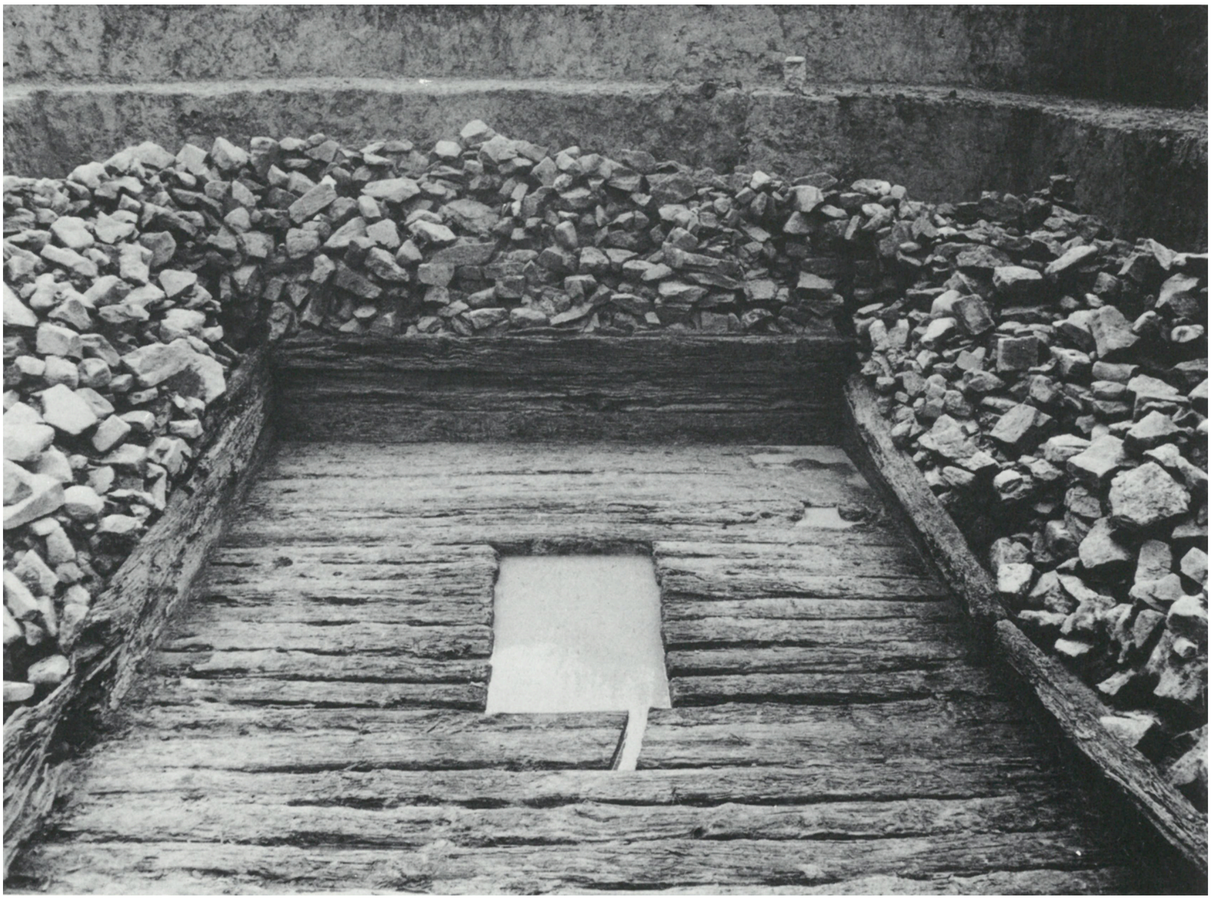

Supplementary Figure 1.13. The uncovered burial chamber of the Magdalenenbergle during the excavation in 1970 (Spindler 1980, Taf.26b, Foto: Gretel Gallay)[1].

The female burial of Ditzingen-Schöckingen

In 1951, during construction works in the area of a barn in the built-up centre of Ditzingen-Schöckingen, the remains of a richly furnished grave were unearthed (Supp. Fig. 1.16). Situated approximately 0.4 m below the foundations of a wall, the poorly preserved skeletal remains of a female, aged around 20-40 years and oriented in a south-north direction, were found within a burial pit surrounded by larger stone fragments. Some of the displaced items of clothing were discovered in the spoil of the foundation trench. Originally, nine golden rings, six bronze pins with heads made of decorated gold sheet, and four pins with coral heads formed an eye-catching headdress in the cranial area (Supp. Fig. 1.14 & 1.16). A necklace of nine coral beads and a closed plain bronze neck ring probably adorned the neck area. Of particular note is the centrally placed large coral bead of the necklace; it is not made of one piece but was assembled from several segments connected with thin bronze pins. The deceased wore three golden bracelets with ribbed decoration on each forearm, and a bronze ankle ring on the right foot. Further items of dress comprise three spiral arm bands with snake head ends (Supp. Fig. 1.15), the closest comparative objects of which are to be found in northern Italy and Slovenia.

As only the burial pit was excavated, no definitive statement can be made about the surrounding features. Thus, it remains uncertain whether a burial mound had once covered the grave or if it was a shallow burial.

In 1999, during subsequent investigations of the area necessitated by further construction work, another Hallstatt female grave with much simpler burial goods was discovered at a distance of about 20 metres.

The Ditzingen-Schöckingen ceremonial tomb is culturally linked to the centre of power at Hohenasperg, approximately 10 km away.

Bibliography:

Paret, O. Das reiche späthallstattzeitliche Grab von Schöckingen (Kr. Leonberg). Fundberichte Schwaben N. F. 12, 1938-1951, 37-40.

Hoppe, Th. Allein unter Männern. Die „Dame“ von Schöckingen. In: J. Biel (ed.), Steinzeitdorf und Keltengold. Archäologische Entdeckungen zwischen Alb und Neckar. Archäologische Informationen aus Baden-Württemberg 78 (Esslingen 2018) 90-94.

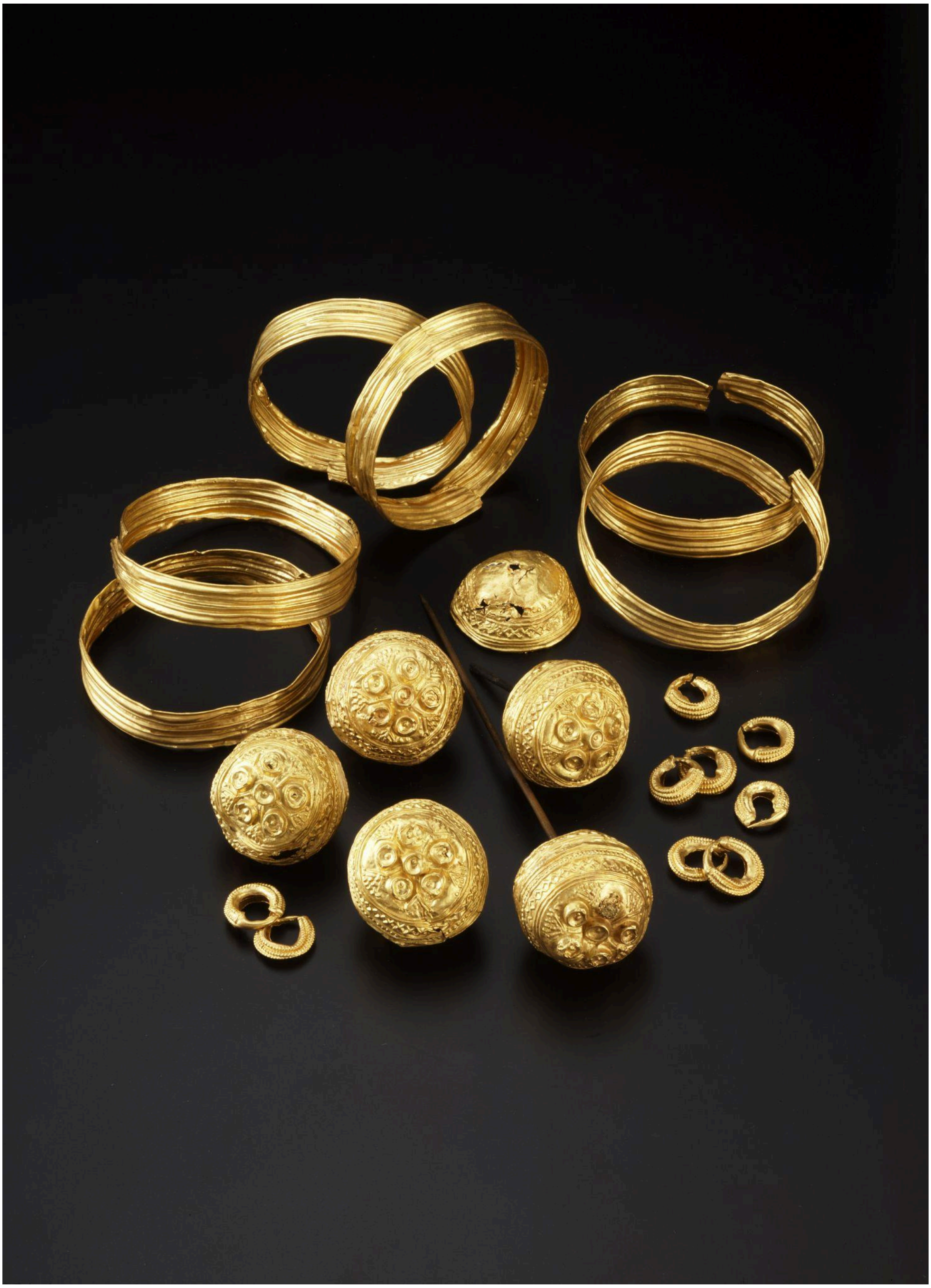

Supplementary Figure 1.14. Gold jewellery of the Lady of Ditzingen-Schöckingen (Landesmuseum Württemberg, P. Frankenstein / H. Zwietasch).

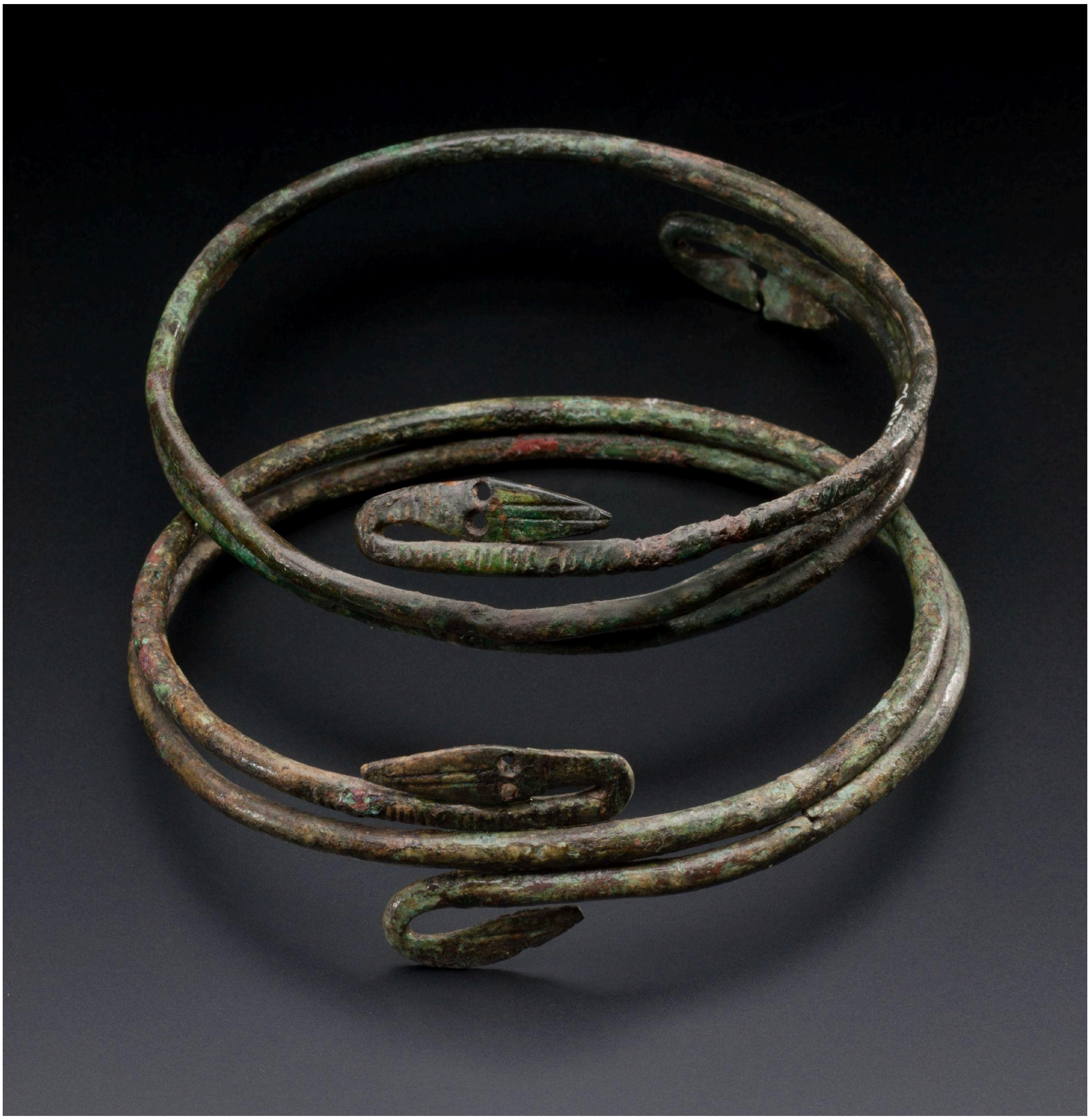

Supplementary Figure 1.15. Bronze spiral arm rings with snake head ends and coral inlays (Landesmuseum Württemberg, H. Zwietasch).

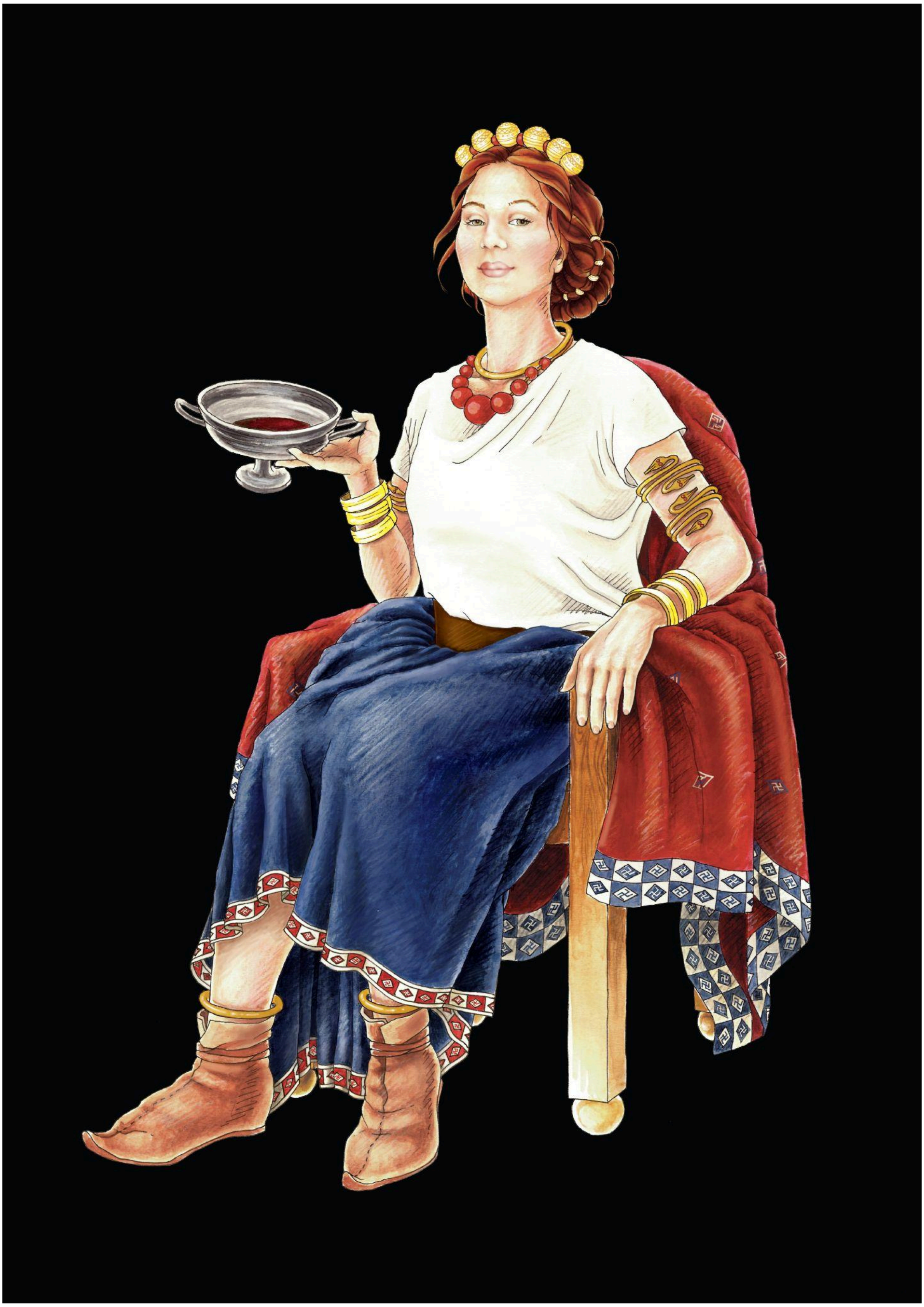

Supplementary Figure 1.16. Likeness of the Lady of Ditzingen-Schöckingen (Landesmuseum Württemberg, L. Lubbersen, Studio Lindalu e.K).

Supplementary Note 2: Kinship, Inbreeding and individual ancestry

Kinship

We calculated pairwise mismatch rates (PMMR) between all 31 early Celtic individuals to estimate the degrees of biological relatedness[2] (Supp. Fig. 2.1, Supp. Table 2.1). We subsequently visualised the pairwise difference matrix as heatmap plot, applying Ward's minimum variance method (ward.D2) for hierarchical clustering of the distances as well as calculated the coefficient of relatedness *r* between each pair of individuals using the formula $r = (PMMR_{average}-PMMR_{observed})/PMMR_{identical}$ (Supp. Fig. 2.2b).

We observe four clusters within the dataset (Supp. Fig. 2.1). Cluster 1 contains the central graves of Asperg-Grafenbühl (APG001) and Hochdorf-Eberdingen (HOC001), reflecting a second-degree relationship on the maternal side ($r = 0.41$, both individuals share the mtDNA haplogroup J1b1a1). While the coefficient of relationship *r* is higher than what might be expected for a second-degree relative (~0.25), KIN (Supp. Table 2.4), IcMLkin[3] (Supp. Fig. 2.2c, Supp. Table 2.2) and BREADR (Supp. Fig. 2.3a) indicate a second-degree rather than a first-degree relationship. To maximise our resolution, we filled missing data in the single-stranded libraries of APG001 and HOC001 with additional genotypes present in the double-stranded but not in the single-stranded libraries. Subsequently, the pair exhibits a PMMR of 0.2095 ± 0.005 (based on 6970 overlapping SNPs). Using the binomial test implemented in BREADR, we find that a first-degree relationship (expected PMMR = 0.1906) is strongly rejected ($p = 7.08e-05$). Similarly, the normalised posterior probability for a first-degree relationship is considerably smaller (0.0121) than the posterior probability for a second-degree relationship (0.9879) (Supp. Fig. 2.3a).

Cluster 2 contains the two outlier individuals MBG004 and MBG016 from the Magdalenenberg, which both exhibit excess southern European ancestry and are second-degree related (PMMR 0.2118 ± 0.003, $r = 0.31$) (Supp. Fig. 2.2c). Again, the *r* value is inflated in comparison to the expected value, however, a first-degree relationship can be ruled out based on the posterior probabilities for each of the degrees of relatedness (BREADR binomial test $p = 5.09e-13$) (Supp. Fig. 2.3b). Additionally, using ancIBD[4], we identify a more distant kin relationship between this pair and the princely burial MBG017. MBG004 and MBG016 share in total 42.5 cM and 96.4 cM long fragments identical-by-descent (IBD) with MBG017, respectively (Supp. Table 2.7). The positions of those identity-by-descent fragments within the genomes are in all three individuals consistent with being inherited from a recent common ancestor (Supp. Fig. 2.4; Supp. Table 2.6). Both MBG016 and MBG017 furthermore share identity-by-descent fragments with HOC004, a secondary burial at Eberdingen-Hochdorf (in total 35.1 cM and 33.6 cM, respectively), indicative of 7th-to-8th-degree relatives (Supp. Table 2.6 & 2.7).

Cluster 3 contains two individuals from the Magdalenenberg (MBG003 and MBG009) as well as one individual from Hochdorf (HOC003). MBG009 is a third-degree relative of MBG003 on the maternal side (both share the same mtDNA haplogroup H1c9) ($r = 0.12$) as well as a third-degree relative of HOC003 ($r = 0.14$) (Supp. Fig. 2.3c).

Finally, cluster 4 contains the four Magdalenenberg individuals MBG001, MBG002, MBG011 and MBG012. The coefficients of relationship between those individuals range between 0.09 and 0.11, which suggests kin relations on the scale of third-to-fourth degree (r = 0.125 and r = 0.0625, respectively), yet, the low coverage of those individuals prevent us from obtaining a higher resolution.

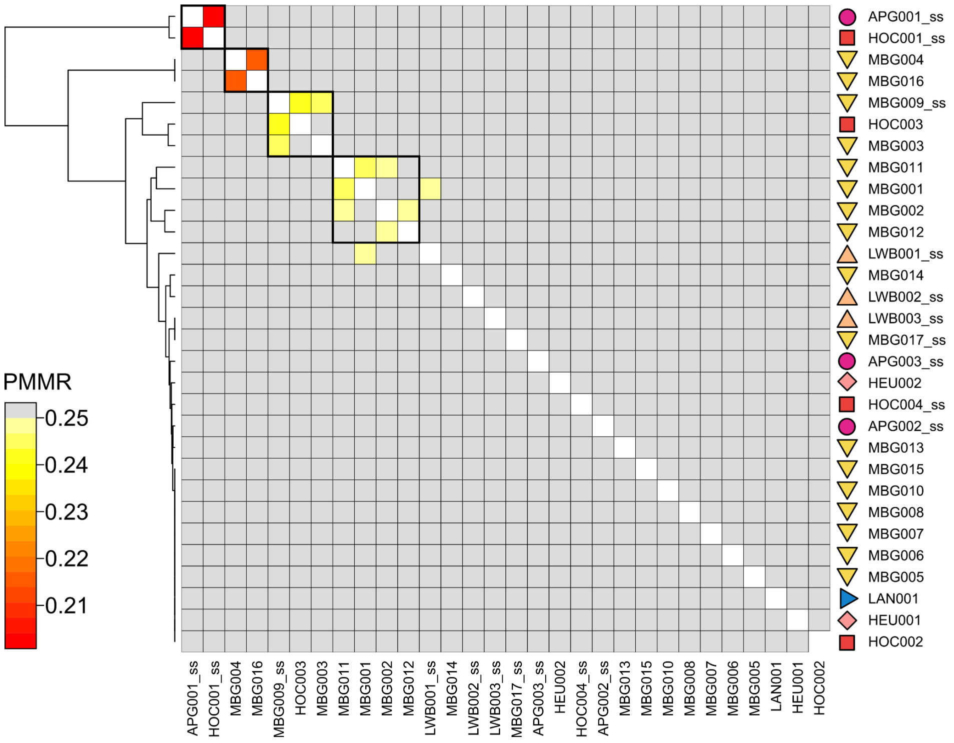

Supplementary Figure 2.1. Clustering of early Celtic individuals based on biological relatedness. Shown are pairwise mismatch rates between pairs of 30 samples. Hierarchical clustering of the pairwise distances using Ward's minimum variance method (ward.D2) is depicted as a dendrogram.The symbols and colours correspond to Figure 1.

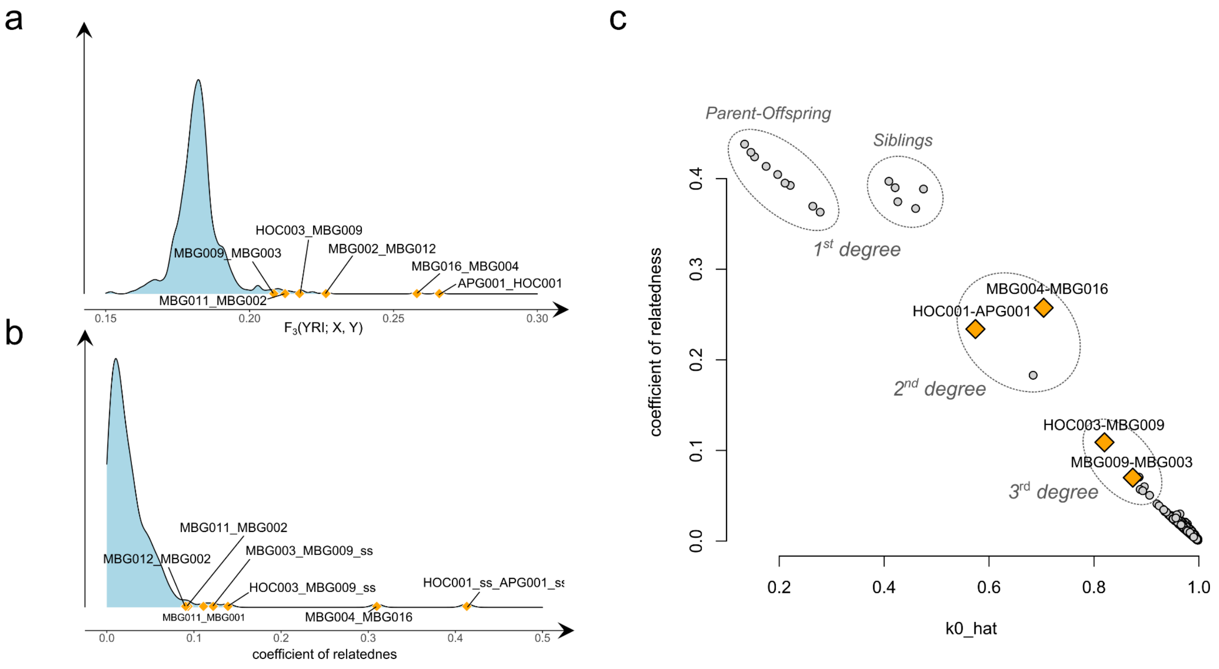

Supplementary Figure 2.2. Fine-scale familial relationships between early Celtic individuals. a) Histogram of outgroup f_3 statistics between pairs of individuals X and Y of the form f_3(YRI; X, Y), where present-day Yoruba from Nigeria are used as outgroup. **b)** Histogram of coefficients of relatedness *r* between pairs of individuals estimated from pairwise nucleotide mismatch rates. **c)** Estimates of genetic relatedness up to the third degree using LcMLkin (reference data from previously published[5] individuals are depicted as grey dots).

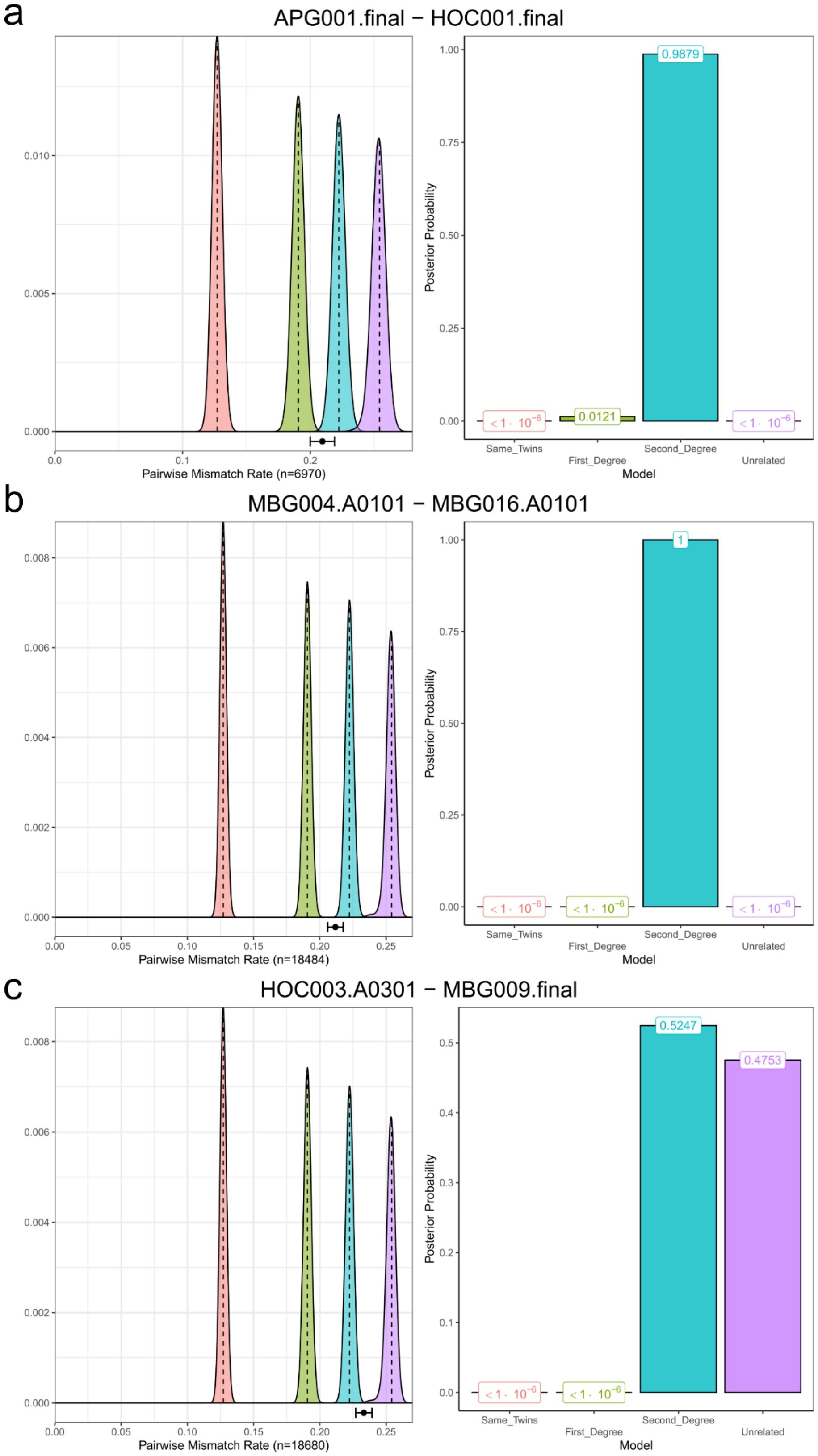

Supplementary Figure 2.3. Fine-scale familial relationships between early Celtic individuals. a) Binomial distribution of PMMR values for each degree of relatedness, given the number of overlapping SNPs, with the observed average PMMR (and 95% confidence interval) between APG001 and HOC001 displayed below. In the right panel are the normalised posterior probabilities from the binomial test implemented in BREADR for each of the degrees of relatedness, indicating the certainty with which the degree of relatedness with the highest posterior probability was chosen. **b**) Same for the mean PMMR between MBG004 and MBG016. **c)** Same for the mean PMMR between HOC003 and MBG009.

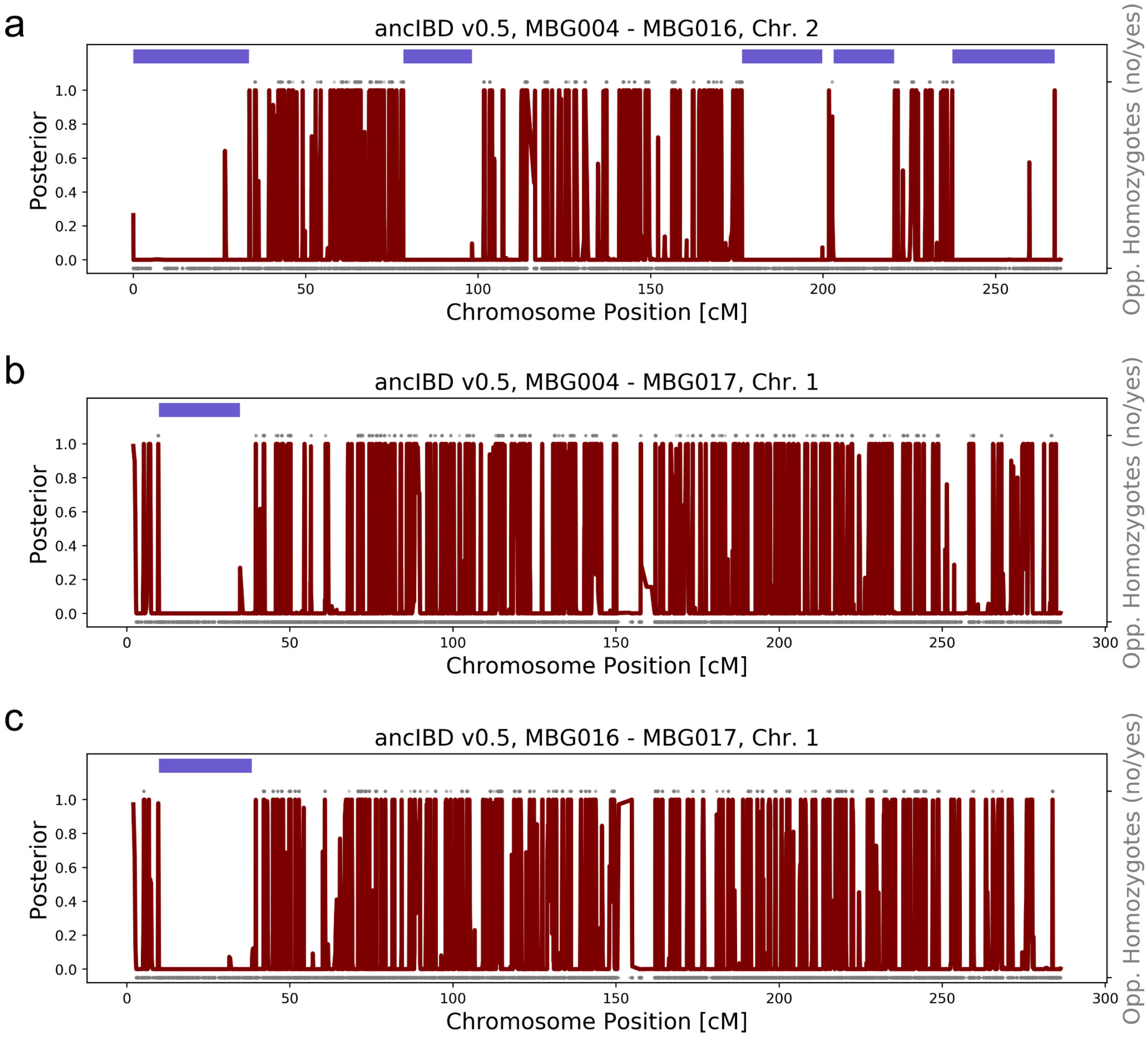

Supplementary Figure 2.4.Detection of biological relatedness using ancIBD. a) Posterior of non-IBD state on Chromosome 1 between MBG004 and MBG016. We also plot opposing homozygotes (gray dots up), whose absence is a necessary signal of IBD. Only SNPs where both markers have an imputed genotype probability greater than 0.99 are plotted. b) same as a) but for MBG004 and MBG017. c) same as a) but for MBG016 and MBG017. IBD sharing results per chromosome per pair of individuals can be found in Supp. Table 2.6.

mtDNA haplogroups

The identified mtDNA haplogroups are typical for the European variation (Supp. Table 1.1). Two cases of identical mtDNA haplogroups were observed. The third-degree related Magdalenenberg individuals MBG003 and MBG009 exhibit both haplogroup H1c9, indicating a shared recent maternal ancestor. The same is true for the Asperg-Grafenbühl APG001 and Hochdorf-Eberdingen HOC001 individuals, who both share the haplogroup J1b1a1 (exhibiting two shared private mutations at the positions 8752 in the ATP6 gene and 11151 in the ND4 gene). Interestingly, for two individuals (HOC004 and SCN001) which were previously assumed to be possible relatives of APG001 and HOC001 based on comparisons of the hypervariable region (HVR) of the mtDNA[6], we reconstructed highly divergent haplogroups (U4c2a and H3g, respectively). In contrast, for APG003, our mtDNA haplogroup classification (X2b) is consistent with previous analyses of the control region, HVR1 and HVR2[7]. To visualise the evolutionary relationships between the mtDNA genomes, we aligned the consensus sequences using MAFFT[8] and constructed a Maximum likelihood tree in MEGA (v7)[9] (Supp. Fig. 2.5).

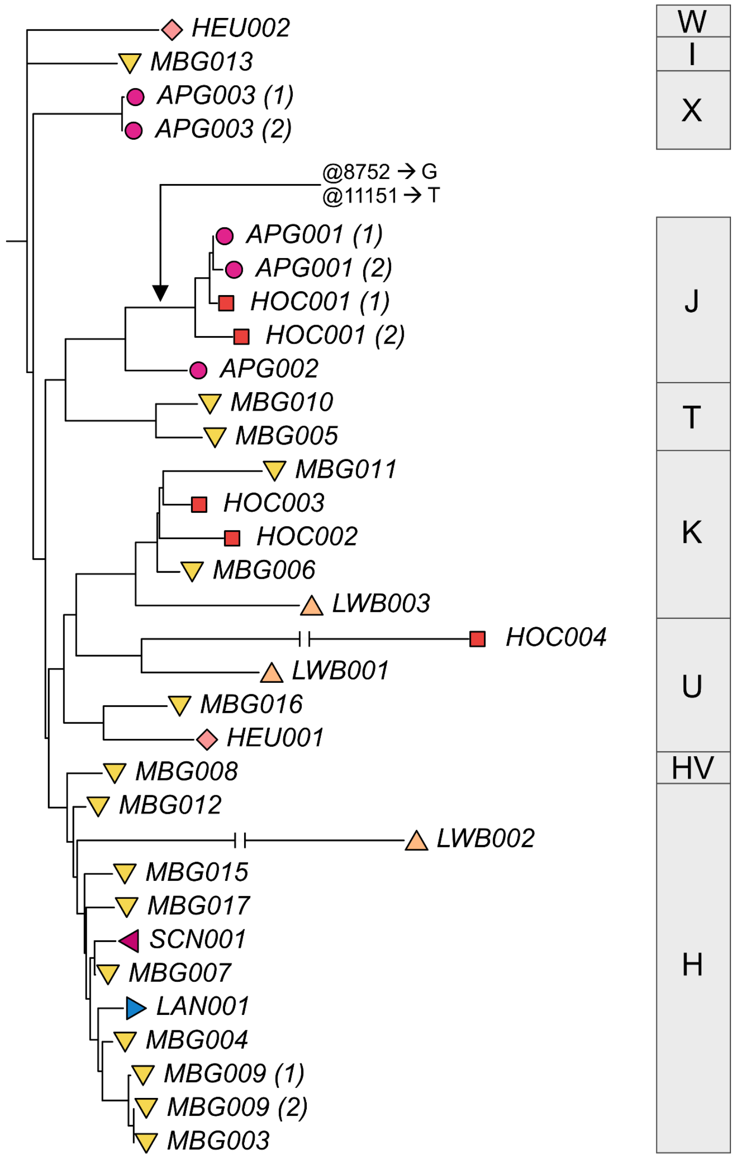

Supplementary Figure 2.5. Phylogenetic tree of mtDNA sequences. Shown is a maximum likelihood tree of 33 mtDNA consensus sequences. For the major clades, the corresponding mtDNA haplogroups are depicted. The symbols and colours correspond to Figure 1.

Inbreeding

Runs of Homozygosity (RoH) were jointly inferred with contamination rates using the hapCon_ROH[10] implementation in hapROH[11] (Supp. Table 2.5, Supp. Fig. 2.6). We plot the results for all individuals for which RoH was detected, yet, we highlight that only individuals with more than 300,000 SNPs provide reliable results. While several individuals show short RoH and in general low sums of RoH (e.g. MBG006, MBG007, MBG016, APG001, LAN001), only two individuals show sums of RoH indicative of recent consanguinity (Supp. Fig. 2.6). Those two individuals are APG003 and MBG004, who exhibit between 160 cM and 240 cM RoH. These values are closest to the ones expected in children of first cousin parents (Supp. Fig. 2.7). The low number of RoH ranging between 4 cM and 8 cM also rules out the possibility of a small population size as the cause of the elevated sum of RoH[11].

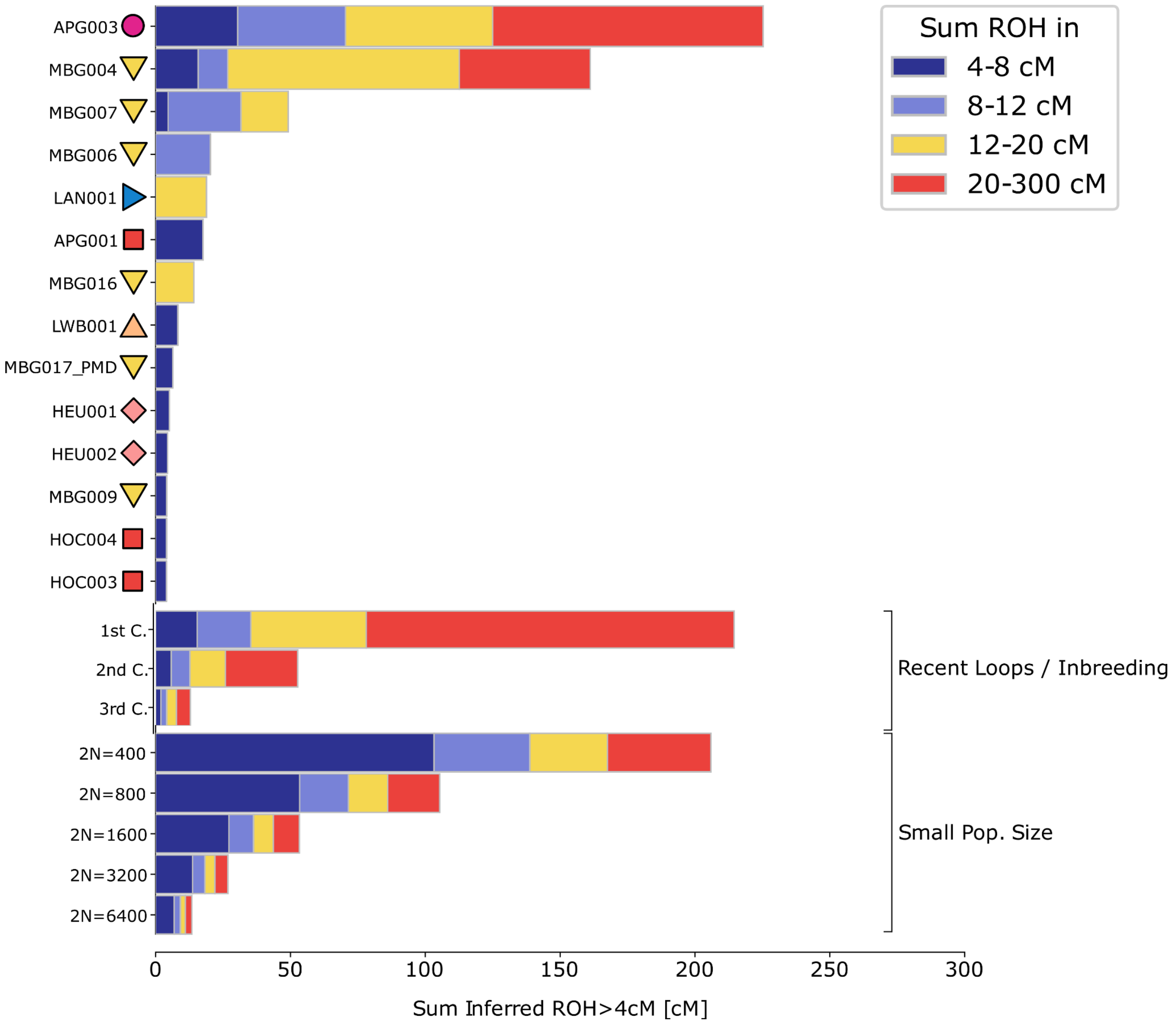

Supplementary Figure 2.6. Phylogenetic tree of mtDNA sequences. Inferred ROH per ancient individual. Expected values for given parental relationships and demographic scenarios are given.The symbols and colours correspond to Figure 1.

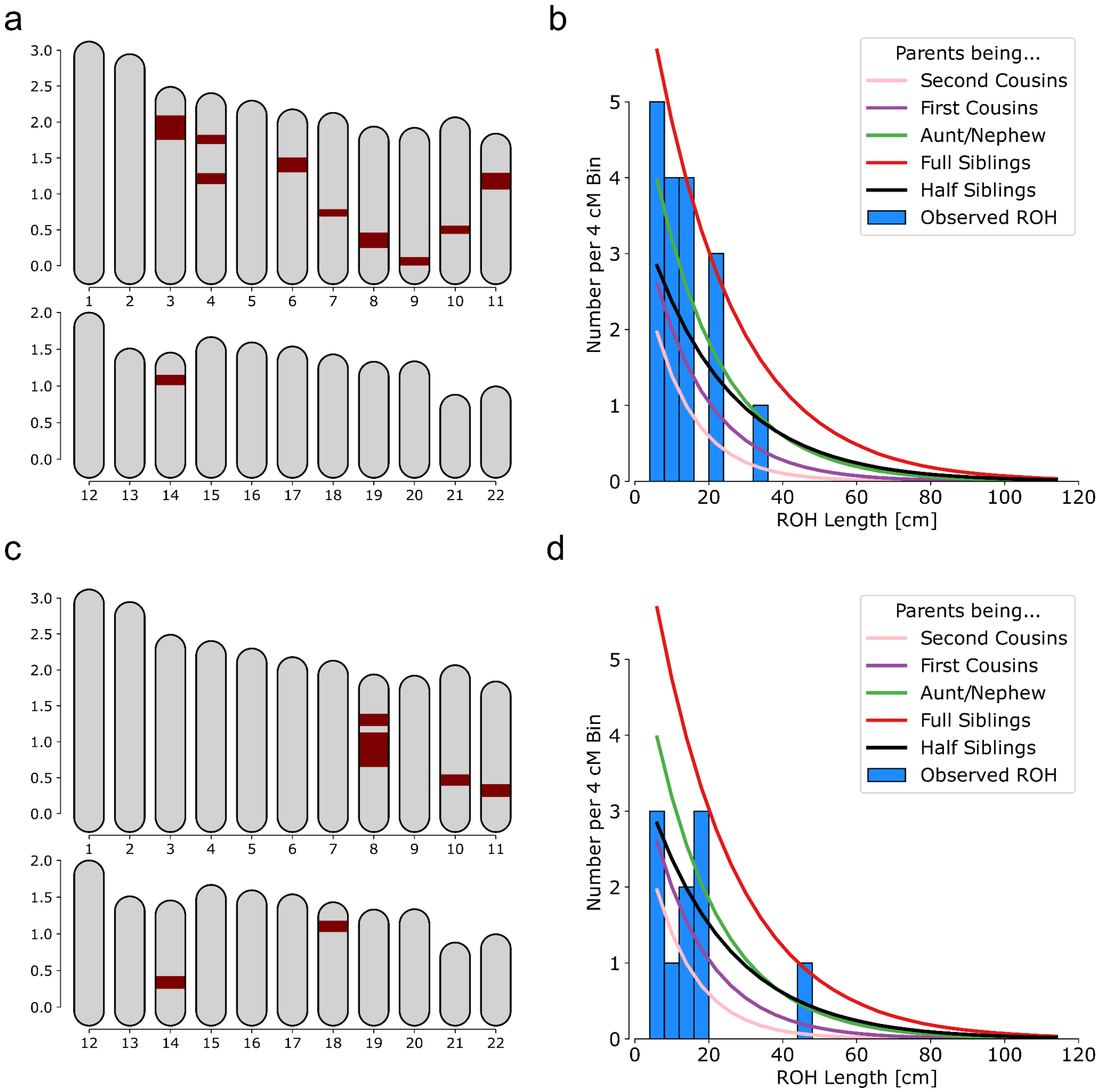

Supplementary Figure 2.7. Evidence for recent inbreeding. a) Distribution of RoH across the genome of APG003, shown as a karyogram. **b**) Distribution of RoH lengths for APG003 compared with several recent inbreeding scenarios.**c)** same for MBG004. **d)** same for MBG004.

Individual ancestry

As shown in Supplementary Note 4, we observe strong genetic affinity between Middle Bronze Age individuals from the Bavarian Lech valley and the early Celtic samples from Baden-Württemberg. Indeed, using qpWave[12,13], most early Celtic individuals can be modelled as receiving 100% of their ancestry from the MBA Lech population (Supp. Table 2.8). However, for three individuals this model fails ($p < 0.01$), namely MBG004, MBG016, and LAN001. While MBG004 and MBG016 show excess affinity to southern European populations, mostly from Iberia and the Italian peninsula (Supp. Table 2.12 & 2.13), LAN001 is genetically closer related to Late Bronze and Iron Age individuals from the Netherlands and Central Germany (Supp. Table 2.11). To obtain more nuanced ancestry proportions for those individuals, we used the supervised ADMIXTURE[14] approach described in Supplementary Note 4 to decompose the ancestry profiles of each individual into 12 components (Supp. Table 3.7).

On average, our early Celtic individuals carry around 65.8% ± 0.04% southwestern European-related ancestry (here: CWE, represented by present-day French and Spanish), the component that peaked in Iberia and Italy during the Bronze and Iron Age (Supp. Table 3.7 & Fig. 4a). The highest proportions of CWE ancestry were found in the Magdalenenberg individuals MBG016, MBG004 and the princely burial MBG017, who carry 97.8%, 93.4% and 94.5% CWE ancestry respectively (Supp. Fig. 2.8). Outside the Magdalenenberg, the highest fraction of CWE ancestry was found in the Hochdorf secondary burial HOC004 (88.6%). Interestingly, standard methods for the detection of biological relatedness as well as IBD-sharing indicate that those four individuals were all related to each other. Consequently, all four individuals also show the highest proportions of EEF-related ancestry among our samples, exhibiting on average 33.7% more CWE and 7.2% more EEF than the other Celitc individuals (Supp. Fig. 2.8). This is expected since CWE ancestry is enriched with EEF ancestry due to the higher proportion of Early European farmer ancestry in southern and western Europe compared to Europe north of the Alps (where EEF-depleted, Steppe-ancestry enriched CNE and NOR ancestry components prevail).
Besides those individuals, the secondary burial MBG010 (for which oxygen and strontium isotopes suggest an Iberian or northern Italian origin) also shows elevated CWE ancestry (80%) in comparison to the average (65.8%).

On the other hand, we also find excess northern European ancestry (here CNE (represented by present-day Denmark, northern Germany and the Netherlands) and NOR (represented by present-day Norway and Sweden) in the La Tène period individual LAN001, who carries around 35.5% CNE + NOR ancestry (Supp. Fig. 2.8) as well as 36.3% WBI ancestry (represented by present-day Ireland, Scotland and Wales) (Supp. Table 3.7). In comparison, the other analysed Hallstatt period individuals exhibit only 7.2% CNE + NOR ancestry on average. We further find excess northern European ancestry in two Magdalenenberg individuals, MBG009 and MBG003, who carry 44.7% and 25.4% CNE + NOR ancestry, respectively (Supp. Fig. 2.8). These individuals are no outliers in terms of oxygen and strontium isotopes, yet they are second-degree relatives and also related to the secondary burial HOC003 in Hochdorf-Eberdingen (who shows no detectable CNE or NOR ancestry).

To closer investigate these differences in ancestry and link them to geographical patterns, we applied MOBEST[15] to the PCA coordinates of our individuals along PC1, PC2, and PC3.

The MOBEST analyses were run using a kernel size of 800. We set the predication grid to 50 by 50 km tiles. As reference we used 5,664 published individuals dating between 150 and 5,000 years BP, located between 29.9° and 70° Lat. and -24° and 70° Long. in EPSG:4326 projection. Related individuals and samples with less than 15k SNPs were excluded. We set the relative search time to 0, thus, the probability surface indicates the highest genetic-geographical match at the mean date of the respective individual. The results are shown in Figure 3.

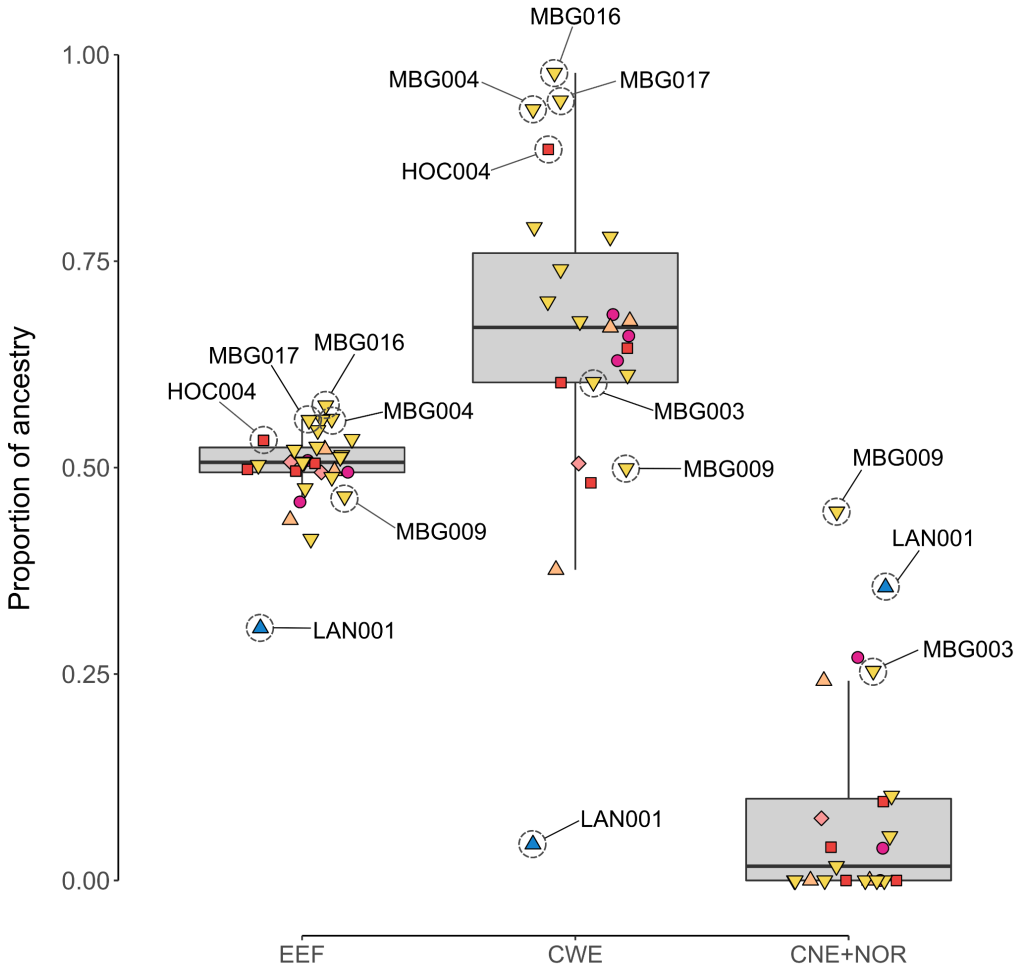

Supplementary Figure 2.8. Southwestern and northern European ancestry in early Celtic individuals. The boxplots show the distribution of EEF ancestry (from qpAdm), CWE (southwestern European) ancestry, and CNE + NOR (northern European) ancestry (from supervised ADMIXTURE) in the Iron Age individuals from southern Germany (*n* = 30 for EEF, *n* = 23 for CWE and CNE+NOR, resp.). The symbols and colours correspond to Figure 1. Bounds of the Box represent the 25th and 75th Percentile. The centre represents the median. Whiskers represent the smallest value greater than the 25th Percentile minus 1.5 times the interquartile range and largest value less than the 75th Percentile plus 1.5 times the interquartile range, respectively. Outliers present the minimum and maximum values in the data.

Isotopes

Previously published[16] (n = 80) and novel (n = 17) strontium and oxygen values from early Celtic individuals from southwestern Germany were analysed jointly (Supp. Table 1.2). Comparisons of $\delta^{18}O$ and $^{87}Sr/^{86}Sr$ values measured in 7 individuals analysed both in Oelze et al. 2012 and in this study showed that the obtained values are highly similar and consistent between both studies. Consequently, published and novel data were plotted on the reference ranges for different geographic areas adopted from Oelze and colleagues[16] (Supp. Fig. 2.9).

We note a high degree of heterogeneity within the Magdalenenberg site, with most individuals showing oxygen and strontium values typical for the Kapf hillfort (the settlement associated with the Magdalenenberg), the Black Forest, and the Hegau region (located in the northwest of Lake Constance)[16,17]. For the female MBG009 and the male MBG003 we observe that their third-degree maternal relationship is also reflected in their similar isotopic compositions. The strontium isotope ratios of both samples are less radiogenic than what is typical for the southeastern Black Forest and point to limestone or other calcareous substrates, which are widely spread in southwestern Germany and beyond, with closest outcrops in the Hegau region and Upper Swabia near Lake Constance or the Swabian Jura. We measured similar $^{87}Sr/^{86}Sr$ ratios in the two second-degree relatives MBG004 and MBG016, pointing to a non-local upbringing for these individuals as well.

However, we detect two outlier individuals, MBG010 and MBG005. While MBG005 shows $^{87}Sr/^{86}Sr$ and $\delta^{18}O$ values typical for the middle Neckar region, MBG010 exhibits a substantially elevated $\delta^{18}O$ level. In contrast to the child APG003 where the excess $\delta^{18}O$ in the sampled deciduous tooth most likely reflects breast milk consumption, a similar enrichment effect can be ruled out for the adult male MBG010. Instead, he might have spent substantial parts of his life in or even originated from coastal southern Europe, most likely the Iberian Peninsula or northern Italy where the crystalline bedrocks of the Alps with higher $^{87}Sr/^{86}Sr$ values join the younger glacial sediments of the plain[16,17]. However, while MBG010 shows excess CWE ancestry in comparison to most of our studied Hallstatt individuals (Supp. Fig. 2.8), his genetic make-up is indistinguishable from Bronze Age genomes from southern Germany, indicating that (most of) his ancestors were not of south Alpine origin. Consequently, this observation highlights the continent-wide, transient mobility networks established by the Hallstatt society of southern Germany.

The two individuals from the Heuneburg both fall slightly outside the range measured at this site. HEU002 shows strontium and oxygen values consistent with the Hegau region, while HEU001 features values that are similar to the middle Neckar region[16,17]. Notably, several animals (cattle and pigs) from the Heuneburg site were based on their strontium values classified as potential imports from the southern Black Forest, most likely the Kapf settlement[18], indicating some social and economic connection to the regions east of the Heuneburg[16].

The individuals from Ditzingen-Schöckingen, Asperg-Grafenbühl and Hochdorf-Eberdingen all cluster, with two exceptions, inside the range expected for the *Muschelkalk* (shelly limestone) outcrops of the area or the bottoms of the Neckar and Enz river valleys[16,17], and can thus be considered local to the middle Neckar region. The two exceptions are HOC003

and APG003, which both show $^{87}Sr/^{86}Sr$ ratios that are more radiogenic than what is typical for the biologically available strontium of the loess-derived soils in the near vicinity of the Hochdorf site. The nearest possible sources are the Keuper sediments in the Stromberg and Heuchelberg hills a few kilometres north of the site. However, their strontium and oxygen isotope values examined together are consistent with them being raised at the Kapf settlement close to the Magdalenenberg (Supp. Fig. 2.9).

Finally, as described in the main text and congruent with the genetic evidence, the La Tène individual LAN001 from *Alte Burg* also appears as an outlier. This sample was taken from a permanent premolar, thus, a similar enrichment effect as for APG003 can be ruled out. Accordingly, an origin in an area with warmer climatic conditions or closer to the coast, such as northern Italy, Iberia, France, or coastal northwestern Europe, especially around the non-calcareous glacial deposits in the Netherlands, Denmark and central Germany, the basal geological complexes of the Armorican Massive, or the glacial moraines of Scandinavia[19–22], is the most plausible interpretation.

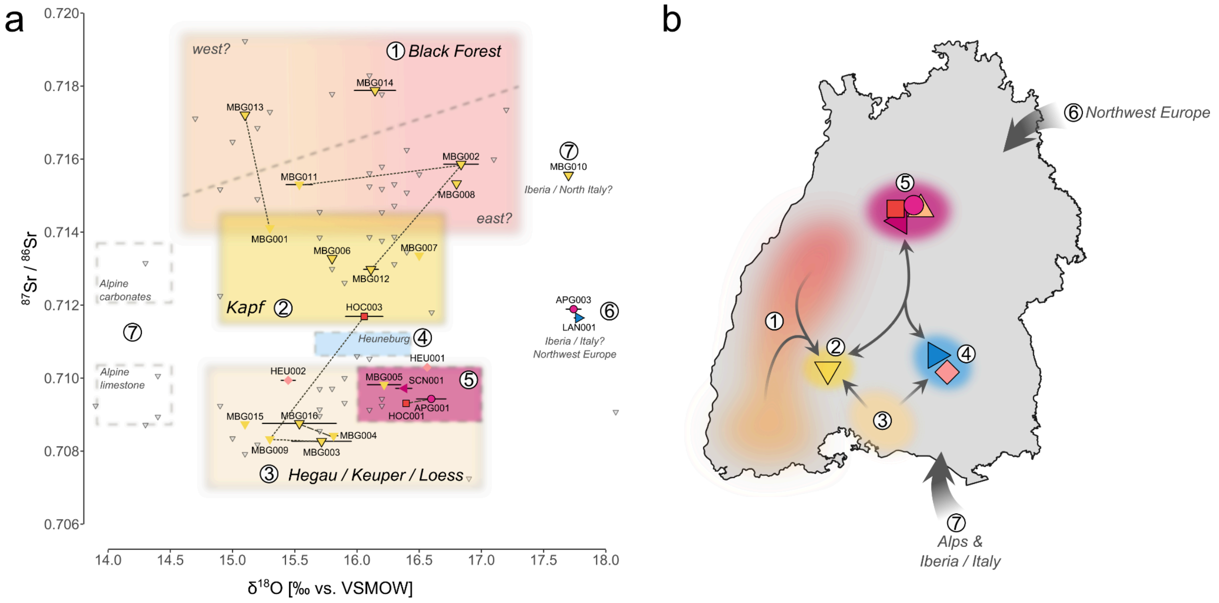

Supplementary Figure 2.9. Strontium and oxygen isotope data indicating individual mobility. a) Coloured symbols highlight 24 individuals from which genetic data was recovered. For eight individuals with genetic data, $^{87}Sr/^{86}Sr$ and $\delta^{18}O$ data from Oelze and colleagues[16] are shown, whereas for 16 samples new isotope data was generated (Supp. Table 2.1). Reference ranges for different geographic areas are adopted from Oelze and colleagues[16]. Dotted lines indicate biological relatedness. The symbols and colours correspond to Fig. 1. Symbols with outlines represent male, symbols without outlines female individuals. Grey triangles depict isotopic data for 59 previously published Magdalenenberg individuals[16]. **b)** Schematic representation of the geographic origin areas of analysed individuals and connections between sites. Colours and numbers represent the approximate ranges for different geographic areas shown in a). Arrows on the map indicate a general direction of individual mobility rather than discrete routes of migration.

Height

The body height of the Eberdingen-Hochdorf ‘prince’ (HOC001) has been calculated at 1.80-1.84 m, which makes him the tallest Iron Age individual from southern Germany to date. With a reconstructed body height of 1.79 m (after Breitinger[23]), the prince of Asperg-Grafenbühl (APG001) is the second tallest individual in the anthropological record of Iron Age southern Germany. As shown above, both individuals are in fact second degree relatives, most likely in the form of maternal uncle (HOC001) and nephew (APG001). We have collected reconstructed body heights (all calculated using the approach described by Breitinger[23]) of several early Celtic individuals from southern Germany, including 18 male and 16 female burials[24–27] (Supp. Table 2.13). We find that male individuals tend to be taller than females (two-sided Wilcoxon rank sum test; $W = 10$, $p = 3.786e\text{-}06$) (Supp. Fig. 2.10a), with an average male height of 171.2 cm (95% CI: 168.98 cm - 173.35 cm) and an average female height of 160.6 cm (95% CI: 159.47 cm - 161.78 cm). Within the male sample, we show that individuals from central graves are in general taller than individuals from secondary burials and non-elite graves (two-sided Wilcoxon rank sum test; $W = 67$, $p = 0.004067$) (Supp. Fig. 2.10b). Male individuals from elite burials exhibit an average body height of 175.3 cm (95% CI: 172.58 cm - 178.09 cm), while individuals from non-elite graves show an average height of 169.1 cm (95% CI: 166.89 cm - 171.27 cm).

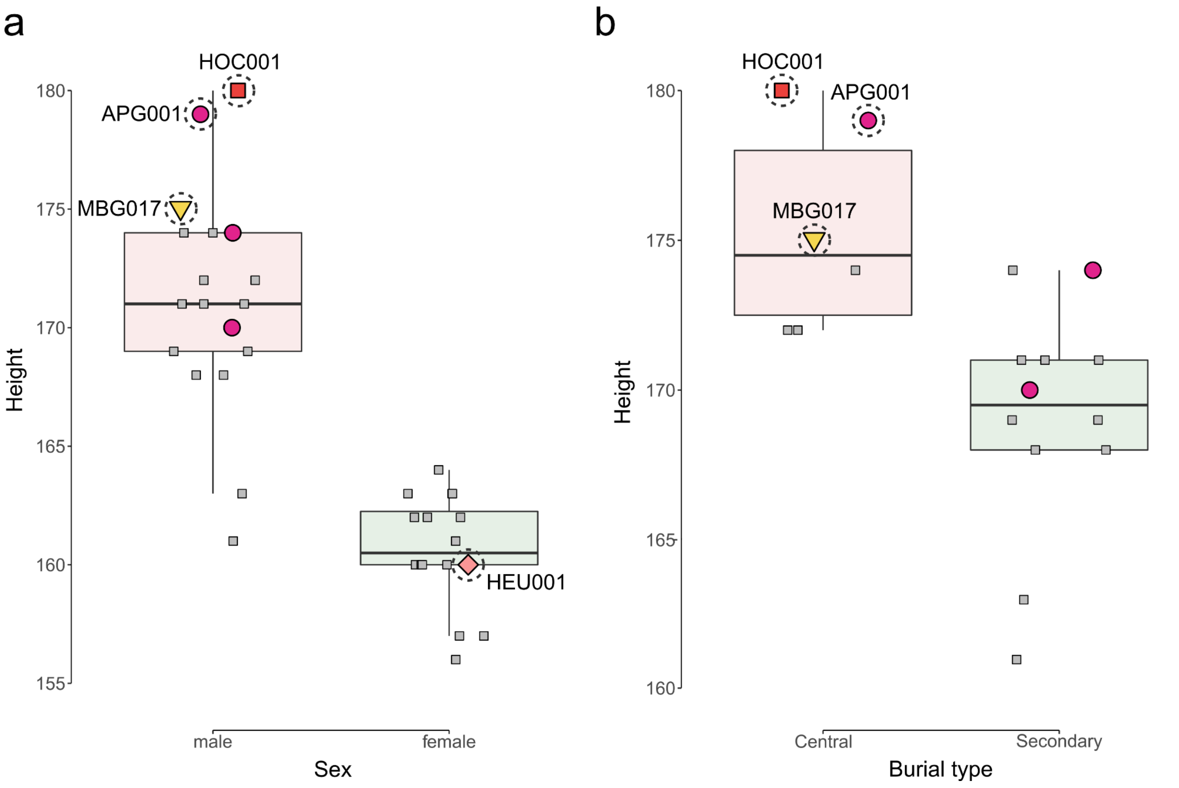

Supplementary Figure 2.10. Variation in body height across Iron Age southern Germany. a) The boxplots show the distribution of reconstructed body heights for 18 male and 16 female individuals. **b)** The boxplots show the distribution of reconstructed body heights for 18 male individuals grouped according to their burial context. The symbols and colours correspond to Figure 1. Bounds of the Box represent the 25th and 75th Percentile. The centre represents the median. Whiskers represent the smallest value greater than the 25th Percentile minus 1.5 times the interquartile range and largest value less than the 75th Percentile plus 1.5 times the interquartile range, respectively. Outliers present the minimum and maximum values in the data.

Comparing archaeological, isotopic and aDNA data

The Hallstatt period in southern Germany was not only characterised by fundamental change in society and technological development, yet, also by the intensification of imports of goods from the Alpine and Mediterranean regions, indicating extensive and continued mobility between the areas[17]. This is especially evident at the Magdalenenberg, where, besides local artefacts typical for the upper Danube and Neckar valleys, a large number of brooches and jewellery of southern provenance was identified. These artefacts have distinct connections to the southern Alpine, North Italian, and Slovenian/Croatian cultural spheres[17]. To integrate this archeological data with our the quantitative data obtained from aDNA and Isotopic analysis, we grouped 16 individuals from the Magdalenenberg (for which both isotopic and aDNA data was available) into a group buried with artefacts of southern provenance (or affiliation) and another group buried without such artefacts. We then compared Early European Farmer (EEF) ancestry (as proxy for southern European ancestry) and $\delta^{18}O$ values (as proxy for transalpine long-range mobility between those two groups. The princely grave, MBG017, was removed from the analysis since it was disturbed and looted, rendering its classification impossible. In general, we find that individuals buried with "southern" (here non-local) artefacts exhibit on average higher proportions of EEF ancestry, indicating a larger proportion of ancestors from southern Europe. Yet, the mean differences in EEF ancestry is only 0.019% (95% CI: -0.062% - 0.025%) and not significant between the groups (two-sided Wilcoxon rank sum test; $W = 23$, $p = 0.4923$). For the $\delta^{18}O$ values, we even find that individuals buried without southern grave goods exhibit on average higher $\delta^{18}O$ values than individuals buried with southern grave goods. Since both Italy and Iberia as well as large parts of France are characterised by higher $\delta^{18}O$ values than southern Germany, this pattern contradicts the notion that Magdalenenberg individuals buried southern grave goods were more often consistent (in terms of their isotopic values) with being immigrants from southern parts of Europe. Yet again, the difference observed between both groups is small (0.525; 95% CI: -0.219 - 1.269) and not statistically significant (two-sided Wilcoxon rank sum test; $W = 44.5$, $p = 0.1283$).

The inconsistency between archaeological, isotopic, and genetic data is also visible on the individual level. For example, the only individual that shows clear isotopic evidence for a non-local, southern European origin is male MBG010. Yet, this individual is not buried with southern grave goods nor is he an outlier in terms of EEF ancestry. On the other hand, female MBG004 (a relative of the central grave MBG017) is buried with southern grave goods and is an outlier in terms of excess EEF ancestry, yet, her $\delta^{18}O$ values are within the range typical for southern Germany. Her second-degree relative MBG016, who also exhibits excess EEF ancestry, is again buried without southern grave goods and is also not an outlier in terms of his $\delta^{18}O$ value. Thus, it is evident that, for the Magdalenberg population, southern grave goods do not constitute a reliable marker for non-local origin of the buried individuals (although individuals of southern European origin were present at the site as shown by aDNA and isotope analysis[16,17]). This indicates that the southern European newcomers were integrated into the Hallstatt society of southern Germany, and that the representation of these southern European contacts and cultural transfer within the burial custom was of importance for the local population[16,17].

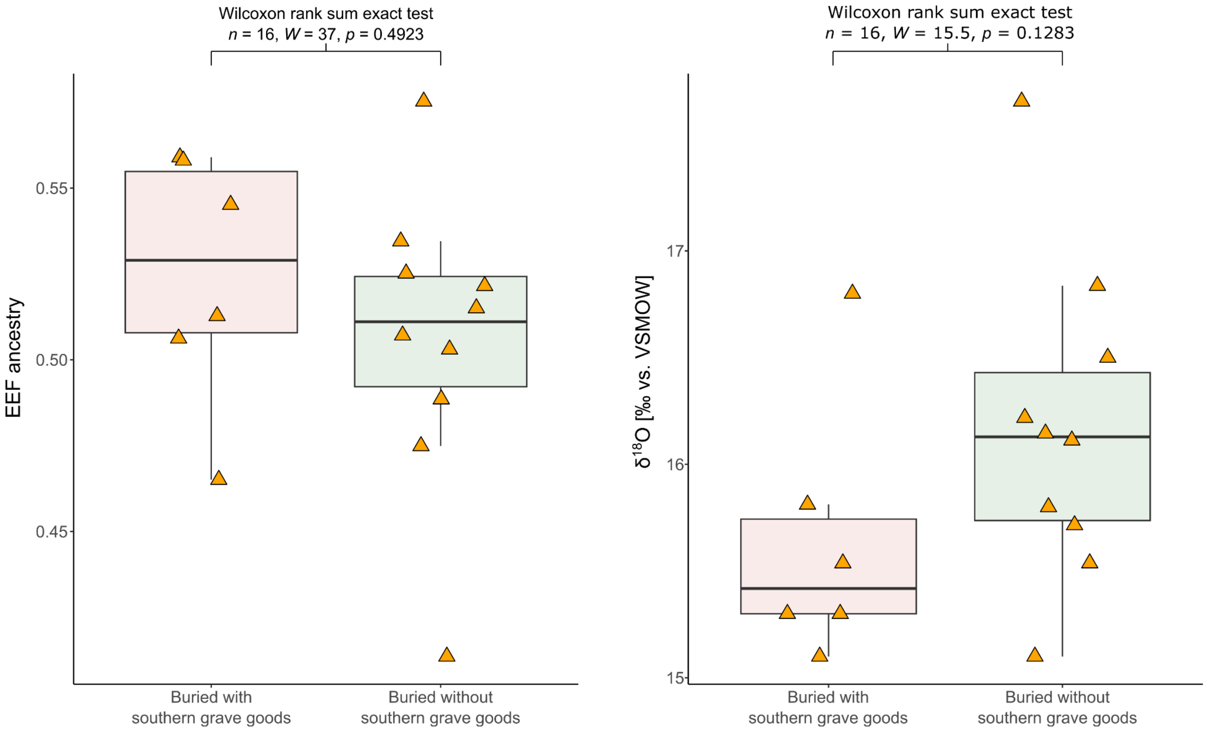

Supplementary Figure 2.11. Variation in aDNA and isotopic data across Magdalenenberg individuals buried with and without "southern" grave goods. a) The boxplots show the distribution of EEF ancestry (as proxy for southern European ancestry) for 10 individuals with and 6 individuals without southern grave goods. **b)** The boxplots show the distribution of $\delta^{18}O$ values (as proxy for long-range mobility) for 10 individuals with and 6 individuals without southern grave goods. The symbols and colours correspond to Figure 1. Bounds of the Box represent the 25th and 75th Percentile. The centre represents the median. Whiskers represent the smallest value greater than the 25th Percentile minus 1.5 times the interquartile range and largest value less than the 75th Percentile plus 1.5 times the interquartile range, respectively. Outliers present the minimum and maximum values in the data. The results of a two-sided Wilcoxon rank sum exact test between both groups are indicated above the boxplots.

Supplementary Note 3: Latent pedigree modelling

Introduction

Given our previous analysis on kinship and mitochondrial DNA, we evaluate probabilistically how the two princely burials from Eberdingen-Hochdorf (HOC001) and Asperg-Grafenbühl (APG001) are related to each other. We first collect the following central observations from Genetic analyses:

1. APG001 and HOC001 are genetically related as second degree or first degree, according to pairwise autosomal mismatch rates (see Supplementary Note on Kinship 2).
2. APG001 and HOC001 share the same mitochondrial sequence, so must be closely maternally related.
3. APG001's parents are not closely related to each other (see Supplementary Note 2 on Inbreeding), given a lack of extended runs of homozygosity in APG001's genome.

Furthermore, we have the following archaeological and anthropological evidence for the two burials:

1. HOC001 was buried between 540 and 520 BCE [28–30]
2. HOC001 probably died aged 40-50 years old [28,31]
3. APG001 was buried between 500 and 480 BCE [28]
4. APG001 probably died aged 20-40 years old [31,32]

These constraints leave a finite set of possibilities for the two individuals to be related. Specifically, the following relationships are consistent with a first or second degree relationship (see Supp. Fig. 3.1 for specific examples of each of these):

1. HOC001 was APG001's father, or vice versa (1st degree)
2. HOC001 was APG001's full sibling (1st degree)
3. HOC001 was APG001's half sibling (2nd degree)
4. HOC001 was APG001's uncle, or vice versa (2nd degree)
5. HOC001 was APG001's grandfather or vice versa (2nd degree)
6. HOC001 and APG001's were double-first-cousins (2nd degree), meaning that one of HOC001 parents and one of APG001's parent were 1st cousins, as were the other two.

However, some of these scenarios are unlikely given the very close mitochondrial relationship between the two, and the absence of long runs of homozygosity in the younger individual, as we will discuss further below. We will first proceed now with introducing the modelling itself and some visualiations of posterior distributions that help getting intuition for the likelihood of the different scenarios. The main part of this section is the last section, "model comparison", where all models are discussed.

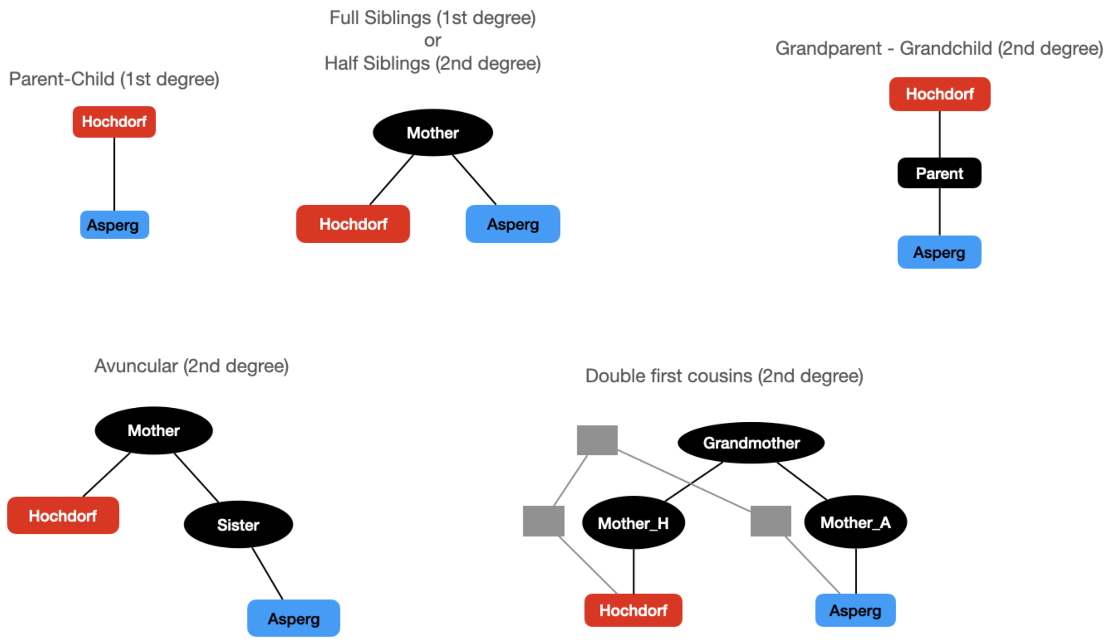

Supplementary Figure 3.1: Plausible pedigrees connecting Hochdorf (HOC001) and Asperg (APG001) on the maternal line, consistent with the genetic and archaeological/anthropological constraints, see table above.

Model implementation and intuition through posteriors

The general idea for latent pedigree modelling is to consider all members of the pedigree with principally unknown birth and death ages, with priors set by the archaeological and anthropological data. Individuals are connected through their joint ancestors, typically their mother or grandmother. We first express our knowledge of the archaeological and anthropological data through probability distributions.

Burial dates

We generally consider the (unknown) burial dates of both HOC001 (***burial_h***) and APG001 (***burial_a***) to be governed by a Gaussian prior distribution (Supp. Fig. 3.2). Specifically, we have

$$burial_h \sim normal(-\ 530,\ 6)$$
$$burial_a \sim normal(-\ 490, 6),$$

where negative mean values denote the mean BCE dates for the two burials, and the standard deviation of 6 here is chosen such that around 90% of the probability mass are placed within 20 year intervals around the mean.

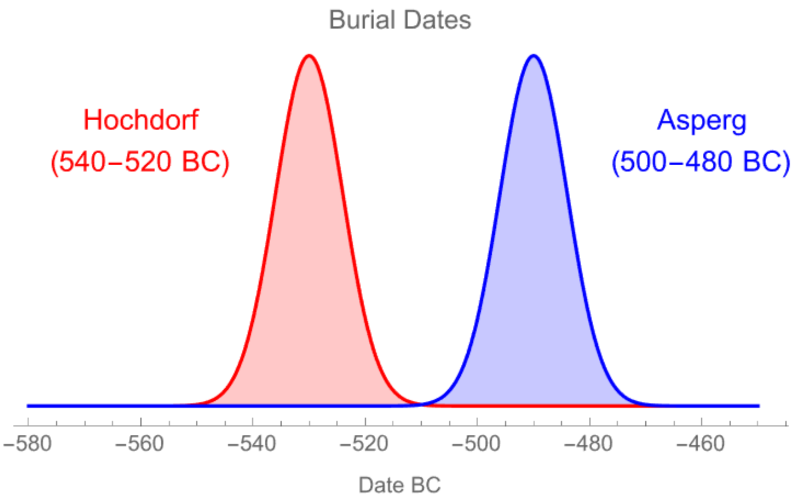

Supplementary Figure 3.2: Burial date prior distributions. The distributions are chosen such that 90% of the probability mass lies within the interval specified in the archaeological literature.

Age at death

Age at death is also governed by normal priors, reflecting the anthropological age estimates (Supp. Fig. 3.3):

$$age_h \sim normal(45,\ 6)$$
$$age_a \sim normal(30, 6),$$

where the means reflect the mean of the osteological age assessment of the two individuals, and the standard deviation again reflects 20 year uncertainties around those dates (in case of HOC001 the osteological assessment suggests 40-50, so only 10 years uncertainty, but we chose to be more conservative and allow for a broader interval in the prior).

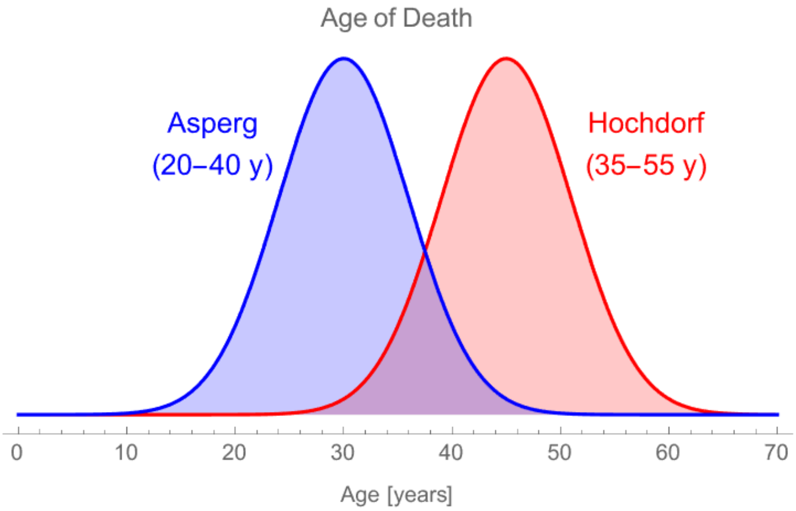

Supplementary Figure 3.3: Age of death prior distributions. The distributions are chosen such that 90% of the probability mass lies within the interval specified obtained from osteological analyses.

Age of Motherhood

For our modelling we require priors for the age at which mothers give birth to children. Important here is that we require not the probability for the mother's age of the *first* child, but for *any* child. There is little quantitative evidence for how this probability would look in prehistory, but a conservative biological assumption appears to be an interval for motherhood between 15 and 45 years of age of the mother [33]. There is, however, a recent quantitative estimate of the mean age of mothers throughout human history [34], which uses the fact that the spectrum of mutations in the germline of the human genome depends on the age of parents, which yields an estimated average of 23.2 years of the average mother's age (and 30.7 for fathers). Taken together, we need a distribution with the following properties:

1. a mean of 23.2 years
2. a 0.005-quantile at 15 years
3. a 0.995-quantile at 45 years

We chose a generalised Gamma distribution for this (which is simply a gamma distribution shifted on the x axis), which has three parameters. When making a least-square fit of such a distribution to the three features above, we obtain a best-fitting prior distribution (Supp. Fig. 3.4):

$$age_m - 14.3744 \sim gamma(2.31385,\ 0.262176)$$

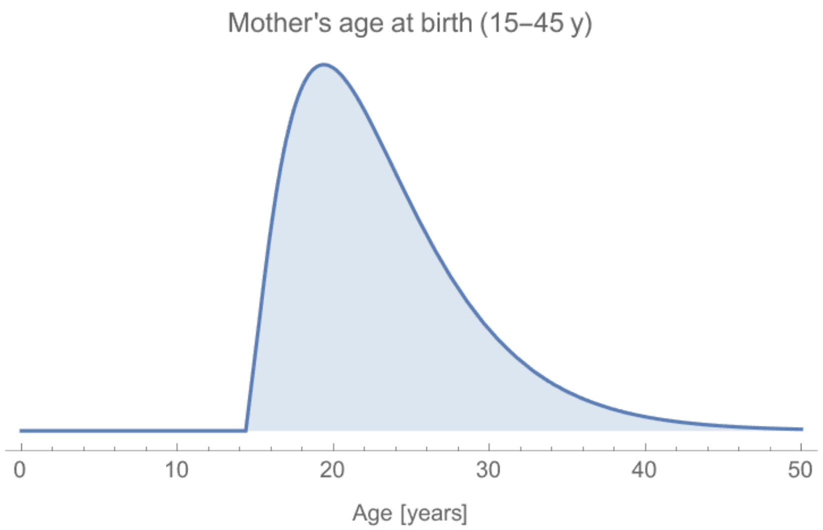

Supplementary Figure 3.4: Age of a mother. The distribution is chosen such that 0.005 and 0.995 quantiles are at 15 and 45 years, respectively. The mean is chosen to be 23.2 years, as reported in recent literature [34].

Age of Fatherhood

For the age at which a man can become a father, we proceed similarly as above, but with the following constraints:

1. a mean of 30.7 years (see again [34])
2. a 0.005-quantile at 15 years
3. a 0.995-quantile at 70 years

Which results in the distribution

$age_m - 13.4481 \sim gamma(2.62396,\ 0.152097)$

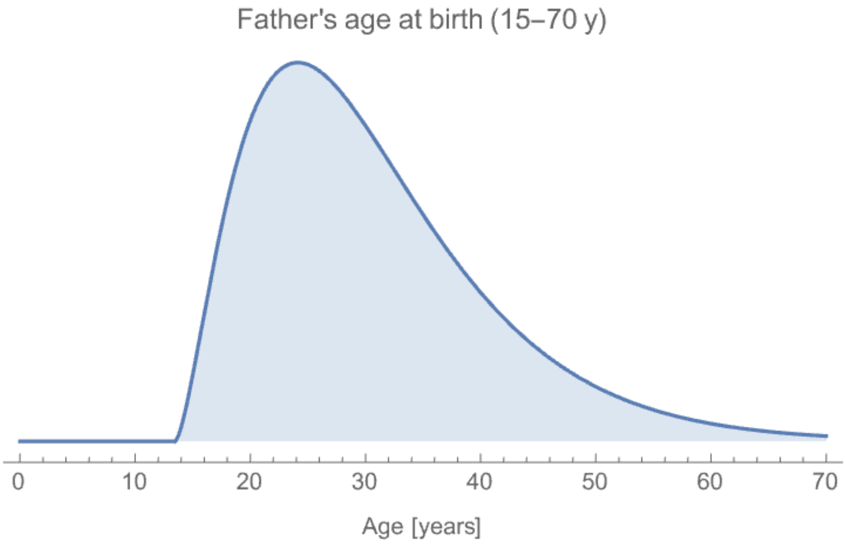

Supplementary Figure 3.5: Age of a father. The distribution is chosen such that 0.005 and 0.995 quantiles are at 15 and 70 years, respectively. The mean is chosen to be 30.7 years, as reported in recent literature [34].

Sibling Model

We define the sibling model, by HOC001 and APG001 having been born to the same mother (Supp. Fig. 3.1). This means that there is a true, and unknown, age of birth of HOC001's mother, and this birth-date, together with the (also unknown) birth-dates of HOC001 and APG001 determines the age of motherhood for both. The model is best summarised as Code in the probabilistic modelling language Stan[35]:

```
parameters {
  real burial_h;
  real burial_a;
  real<lower=0> age_h;
  real<lower=0> age_a;
  real<lower=14.3744> age_m_h;
}

transformed parameters {
  real birth_date_mother = burial_h - age_h - age_m_h;
  real<lower=14.3744> age_m_a = burial_a - age_a - birth_date_mother;
}

model {
  burial_h ~ normal(-530, 6);
  burial_a ~ normal(-490, 6);
  age_h ~ normal( 45, 6);
  age_a ~ normal( 30, 6);
  age_m_h - 14.3744 ~ gamma(2.31385, 0.262176);
  age_m_a - 14.3744 ~ gamma(2.31385, 0.262176);
}
```

The first block defines the independent parameters of the model, which are the two burial dates, the two ages-at-death, and the age of the mother when giving birth to the first child, HOC001 (*age_m_h*).

The second block then defines the birth date of the mother, which is of course simply derived from HOC001's burial date and age, as well as his mothers age. The age of the mother when giving birth to the second child is fully defined by APG001's burial date and age, as well as the mother's birth date. So neither of the two parameters defined in the second block are independent parameters, but fully defined by the five parameters from the first block.

The third block then denotes for each of the five parameters as well as the transformed parameter *age_m_a* its probability according to the chosen priors (see above).

We then use the Stan command line application to perform Markov Chain Monte Carlo sampling for this model, with initial values at the means of the respective prior distributions, yielding a joint posterior distribution for the five parameters.

While the joint posterior cannot be visualised conveniently, we can visualise the marginal posterior distributions for each parameter (Supp. Fig. 3.6).

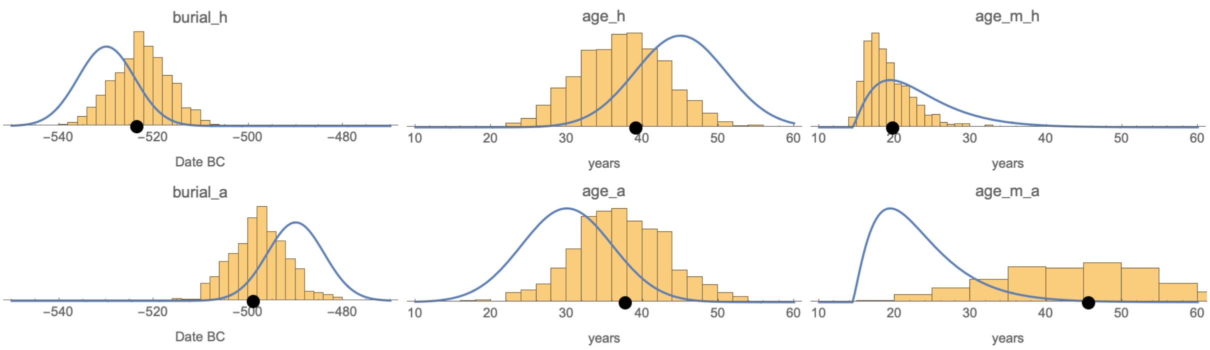

Supplementary Figure 3.6: Marginal Posterior distributions for the sibling model. The marginal posterior distributions are shown for all five independent parameters and age_m_a. The black dot indicates the maximum posterior point.

It becomes clear when looking at the posterior distributions for the sibling model, that most of the posterior weight is outside the specified priors, rendering the model not very well fitting. In particular, the age of the mother when giving birth to her second child (APG001), *age_m_a* is at the upper end of biological plausibility, with the maximum posterior at around 45 years, and much of the posterior weight even older. While the marginal posterior distribution for *age_m_a* does also include younger values, those then would result in less plausible values of all other parameters, which is not visible in the marginal visualisation.

Avuncular model

In the avuncular model considered here, APG001's mother was HOC001's sister (Supp. Fig. 3.1), so we have one more parameter:

```
parameters {
```

```
  real burial_h;
  real burial_a;
  real<lower=0> age_h;
  real<lower=0> age_a;
  real<lower=14.3744> age_m_h;
  real<lower=14.3744> age_m_a;
}

transformed parameters {
  real birth_date_grandmother = burial_h - age_h - age_m_h;
  real birth_date_sister = burial_a - age_a - age_m_a;
  real<lower=14.3744> age_m_s = birth_date_sister - birth_date_grandmother;
}

model {
  burial_h ~ normal(-530, 6);
  burial_a ~ normal(-490, 6);
  age_h ~ normal( 45, 6);
  age_a ~ normal( 30, 6);
  age_m_h - 14.3744 ~ gamma(2.31385, 0.262176);
  age_m_a - 14.3744 ~ gamma(2.31385, 0.262176);
  age_m_s - 14.3744 ~ gamma(2.31385, 0.262176);
}
```

In comparison to the sibling model, we have one additional independent parameter, as now *age_m_a* and *age_m_h* refer to the age of two different mothers. We again introduce some derived ("helper") parameters, to ultimately derive *age_m_s*, which is the age of HOC001's mother when giving birth to HOC001's sister (APG001's mother). The model itself includes one more probability evaluation, now amounting to three birth events reflected by three densities for *age_m_h*, *age_m_a* and *age_m_s*.

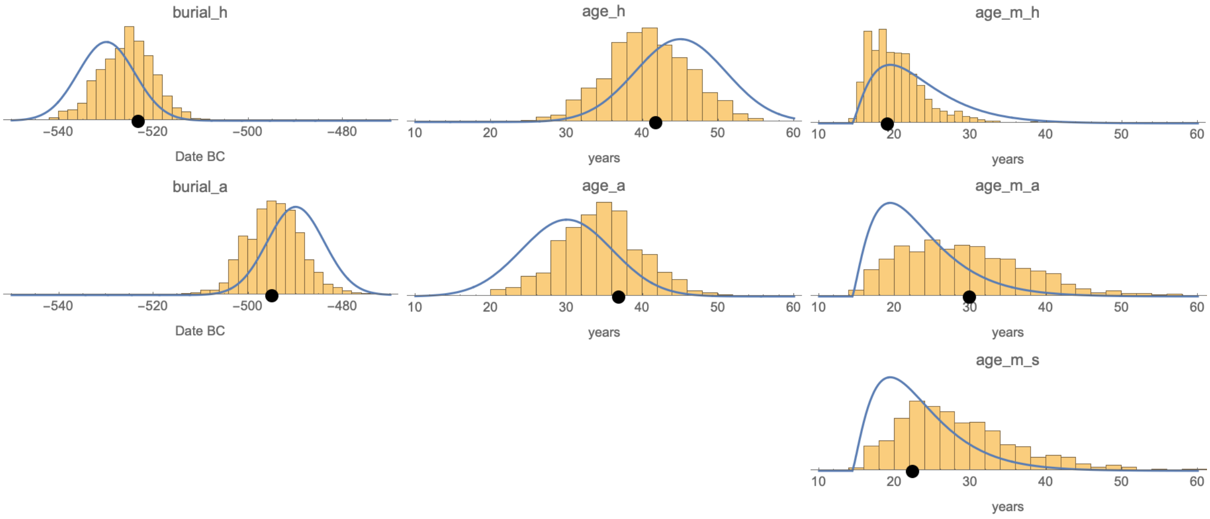

Supplementary Figure 3.7: Marginal Posterior distributions for the avuncular model. The marginal posterior distributions are shown for all six independent parameters and age_m_s. The black dot indicates the maximum posterior point.

Overall, this model fits much better than the sibling model (Supp. Fig. 3.7). In particular, the maximum posterior estimates are all within the prior distributions, especially for the mother's ages, which are all between 20 and 30 years, in contrast to the sibling model, which estimated 45 years as the age of APG001's mother.

Cousin model

The avuncular model has one more parameter than the avuncular model, due to one more latent family ancestor. In this model, the two family sides are connected via their grandmother. We model four birth processes: The two independent births of HOC001 and APG001, with their mothers ages *age_m_h* and *age_m_a*, respectively. And then the births of those two mothers from their shared mother (HOC001's and APG001's grandmother), which are termed age_m2_h and age_m2_a (the age of the grandmother when giving birth to HOC001's and APG001's mother, respectively):

```
parameters {
  real burial_h;
  real burial_a;
  real<lower=0> age_h;
  real<lower=0> age_a;
  real<lower=14.3744> age_m_h;
  real<lower=14.3744> age_m_a;
  real<lower=14.3744> age_m2_h;
}

transformed parameters {
  real birth_date_grandmother = burial_h - age_h - age_m_h - age_m2_h;
  real<lower=14.3744> age_m2_a =
       burial_a - age_a - age_m_a - birth_date_grandmother;
}

model {
  burial_h ~ normal(-530, 6);
  burial_a ~ normal(-490, 6);
  age_h ~ normal( 45, 6);
  age_a ~ normal( 30, 6);
  age_m_h - 14.3744 ~ gamma(2.31385, 0.262176);
  age_m_a - 14.3744 ~ gamma(2.31385, 0.262176);
  age_m2_h - 14.3744 ~ gamma(2.31385, 0.262176);
  age_m2_a - 14.3744 ~ gamma(2.31385, 0.262176);
}
```

The crucial part is the connection between *age_m2_h* and *age_m2_a*, which are not independent parameters because the grandmother is the same person in this case. As can be seen in the “Transformed parameters” block, this non-independence is expressed by the fact that once we know HOC001’s burial date, *burial_h*, his age of death, *age_h*, the age of his mother at his birth, *age_m_h* and the age of his grandmother at her birth, *age_m2_h*, we know the grandmother’s birth date, which then determines *age_m2_a* together with the respective family tree history of APG001 mediated through variables *burial_a*, *age_a* and *age_m_a* (Supp. Fig. 3.9).

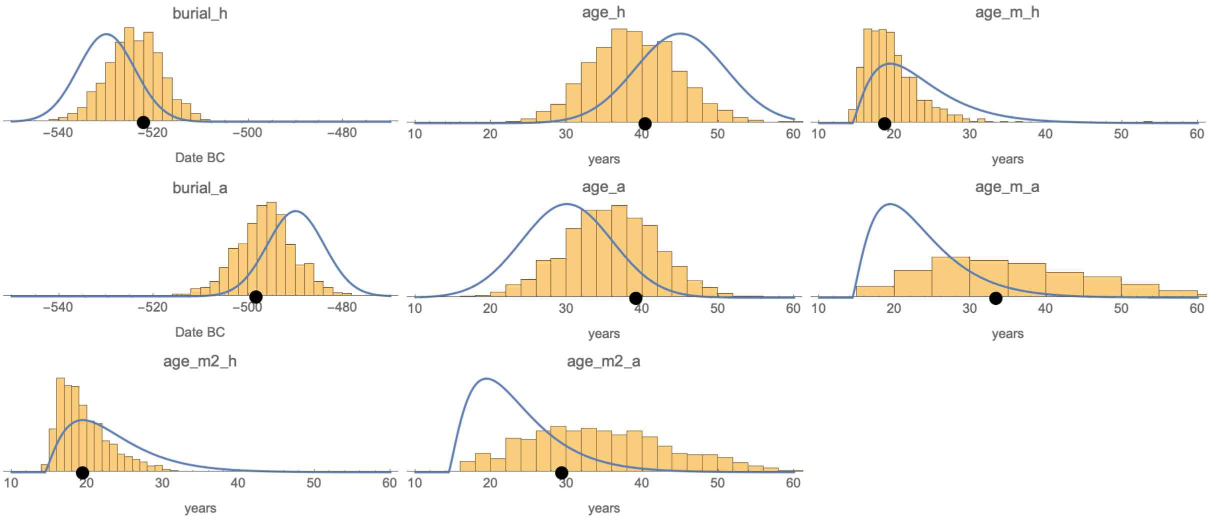

Supplementary Figure 3.9: Marginal Posterior distributions for the cousins model. The marginal posterior distributions are shown for all six independent parameters and age_m2_a. The black dot indicates the maximum posterior point.

Overall, the (double-)cousin model appears to fit better than the sibling model, thanks to the additional degrees of freedom in the birth dates of the mothers of HOC001 and APG001.

Additional models, inconsistent with genetic data

For the sake of completeness, we test two additional scenarios, which are however inconsistent with the mitochondrial DNA and runs of homozygosity, as we will argue below. First, we tested a parental model, in which HOC001 is APG001’s father, and one where APG001 is HOC001’s father. We only here show only the posterior estimates for the first case, which is at least chronologically more likely (Supp Fig. 3.10):

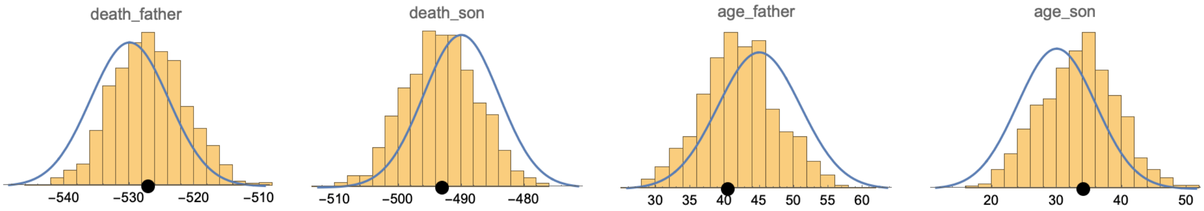

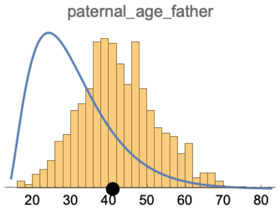

	5-Percentile	Median	95-Percentile	MaxLL
death_father	-535.584	-526.867	-517.61	-527.254
death_son	-502.499	-493.083	-483.553	-493.008
age_father	33.168	42.0746	51.6653	40.5084
age_son	23.5967	33.731	42.8031	34.0113
paternal_age_father	26.584	41.8898	60.2292	40.7424

Supplementary Figure 3.10: Marginal Posterior distributions for the father model. Parameter names are self-explanatory. Model code can be seen on the github-repository, see Code availability statement at the end of the section.

Second, we tested four grand-father models:

1. HOC001 is paternal grandfather of APG001
2. HOC001 is maternal grandfather of APG001
3. APG001 is paternal grandfather of HOC001
4. APG001 is maternal grandfather of HOC001

The first two are very similar, the latter two are extremely unlikely. We here show posteriors only for the first case (Supp. Fig. 3.11):

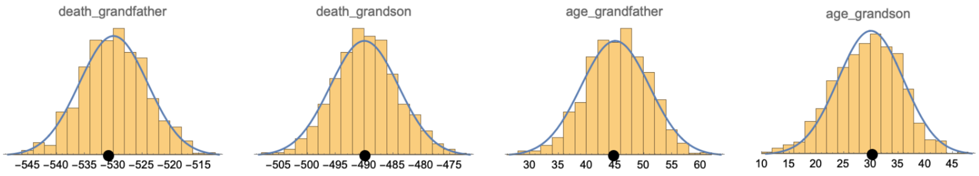

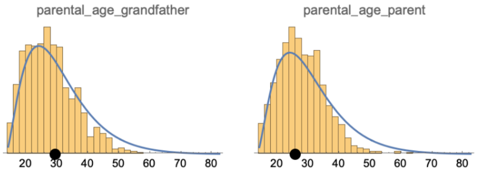

	5-Percentile	Median	95-Percentile	MaxLL
death_grandfather	-539.13	-529.688	-519.954	-530.859
death_grandson	-498.947	-489.708	-480.07	-489.936
age_grandfather	36.4586	45.5126	53.8876	44.8004
age_grandson	19.1795	29.7932	38.5214	30.3085
parental_age_grandfather	16.9716	27.1061	43.2823	29.5939
parental_age_parent	17.0474	27.3295	40.8773	25.8206

Supplementary Figure 3.11: Marginal Posterior distributions for the grandfather model. Parameter names are self-explanatory. Model code can be seen on the github-repository, see Code availability statement at the end of the section.

This model fits very well in terms of matching the archaeological and anthropological data. It is, however, unlikely given genetic evidence, see below.

Model comparison through marginal likelihoods

The intuition gained from the posterior distributions visualised above is: the better the posterior distributions of burial dates, age-at-death and parental ages fit the priors, the better we would consider the "overall fit" of a model. This makes sense: in a good model, we expect the posterior distributions (i.e. the parameter distributions that make the model work)

to be plausible. If a model only works if a mother's age is extremely young, or extremely old relative to biological plausibility, or if the model only works if one of the burials would have to date way earlier or later than we know from archaeology, we would discard the model as non-fitting. We would like to express this "overall fit" of the posteriors by a single number, in order to compare different models.

However, this is not trivial, given the different number of parameters involved. For example, a naive approach might be to compare the maximum posterior values for each model, but the posterior probability itself depends on the number of parameters in a non-trivial way: since every parameter comes with priors, the joint prior and hence also joint posterior for any single point in the parameter space will be lower if there are more parameters (since the overall parameter space is larger and the joint prior and joint posterior are normalised). On the other hand, more parameters also make it easier for the model to find a good-fitting model.

An appropriate way to compare models in this particular case are marginal likelihoods, which are computed as the integral of the model likelihood (i.e. the unnormalised posterior) over the prior. Specifically, we have

$$M = \int p(\theta) L(\theta) d\theta,$$

where θ denotes the parameter vector, $p(\theta)$ is the prior probability, and $L(\theta)$ the likelihood within a specific pedigree model. Note that compared to orthodox Bayesian analysis, our models do not involve "data" in a traditional sense, but only specifies links among the parameters. Due to the fact that the joint prior is normalised over the entire prior domain, the marginal likelihood takes into account model complexity: the more parameters a model has, the larger its prior space. As the integration runs over the entire space, not just over the region of high likelihood, a more complex model will typically be automatically penalised by containing a relatively larger region of the parameter space, and hence typically also a larger region with low likelihood and therefore giving lower marginal likelihoods, unless the model complexity is offset by a substantially better fit.

For example, in case of the sibling model, we would have

Θ = {burial_h, burial_a, age_h, age_a, age_m_h},

and the joint prior $p(\theta)$ would be the product of the priors as specified above in this section, so a product of four specific normal probability densities for the burial dates and ages, and one generalised gamma density for the mother's age, as specified in the Stan code (which implicitly multiplies densities - or rather sums up the log-densities):

```
burial_h ~ normal(-530, 6);
burial_a ~ normal(-490, 6);
age_h ~ normal( 45, 6);
age_a ~ normal( 30, 6);
age_m_h - 14.3744 ~ gamma(2.31385, 0.262176);
```

The likelihood in this example would then be an additional generalised gamma density applied to age_m_a, which in turn is computed as a combination of the five parameters as specified in the Stan model (here shortened):

```
birth_date_mother = burial_h - age_h - age_m_h;
age_m_a           = burial_a - age_a - birth_date_mother;
age_m_a - 14.3744 ~ gamma(2.31385, 0.262176);
```

With these ingredients we can in principle then compute the marginal likelihood as the integral of the Likelihood over the joint prior. This integration, however, is notoriously hard due to the high dimensionality and the often comparably small region of high likelihood within this space.

We here use a relatively new method to compute this integral, published in 2020 by Johannes Reichl [36]. The method uses the posterior samples from our Stan computation, which poses an advantage over alternative methods like Bridge sampling [37] or reversible Jump MCMC [38].

Briefly, the method computes the marginal likelihood by taking the harmonic mean of the posterior samples (which thanks to Bayes formula can be shown to estimate the marginal likelihood). Known instabilities of the naive harmonic mean estimator [39], are then addressed by constraining the integration to a geometric region of high likelihood, defined roughly by a hypersphere. All necessary computational steps are relatively simple to implement, and code samples are given in the original paper [36]. We implemented and tested the method in the Wolfram Language. The notebook and definitions are available online (see Code Availability statement at the end).

In total, we considered 11 models a priori, and their marginal likelihoods and other aspects are summarised in the following table, sorted by marginal log-likelihood:

	**Marginal LLH**	**NrParams**	**Degree**	**MT/ROH consistency**
**H. is A.'s maternal grandfather**	-3.645	5	2	FALSE
**H. is A.'s paternal grandfather**	-3.858	5	2	FALSE
**H. is A.'s father**	-4.989	4	1	FALSE
**H. is A.'s uncle**	-5.675	6	2	TRUE
**H. and A. are double first cousins**	-8.875	7	2	TRUE
**A. and H. are full siblings**	-10.178	5	1	TRUE
**A. and H. are half siblings**	-10.178	5	2	TRUE
**A. is H.'s uncle**	-14.95	6	2	TRUE
**A. is H.'s father**	-23.551	4	1	FALSE
**A. is H.'s maternal grandfather**	-34.207	5	2	FALSE
**A. is H.'s paternal grandfather**	-34.993	5	2	FALSE

Note that in our avuncular models (HOC001 is APG001's uncle or vice versa), we did explicitly not consider the paternal case, where APG001's father would be HOC001's brother, because we already know that those models would be far less likely given that they cannot then explain the matching mitochondrial sequence, see below. While we do explicitly include inconsistent models that ignore this, such as the father- and grandfather models, in case of the avuncular model the maternal model is consistent while the paternal one is not, so we consider it unnecessary to compute the alternative which is so much less likely (see discussion below).

Autosomal relatedness

These above computations yield the marginal model likelihoods given only archaeological and anthropological data. Genetic data has not been factored in yet. In order to do that, we first recall the posterior probabilities from the BREADR[40] analysis for autosomal relatedness, which resulted in a probability of 98.8% for second-degree relatedness and 1.2% for first-degree relatedness. We can therefore add to all likelihoods the log-values for autosomal relatedness as log(0.998) and log(0.012).

Mitochondrial relatedness

In many models, we have "built in" our knowledge of a perfect match of mitochondrial sequences, suggesting a close maternal relationship. In the sibling model, the avuncular models, and the cousin model, the two individuals end up being maternally related by construction. This is not the case for the parental and the grand-parental models. Here, the maternal ancestry in APG001 is by construction separate from the one in HOC001. So normally we would not assume their sequences to match. But since we are dealing with probabilities, we need to cast this into numbers. We consider two possibilities for how their sequences could still match:

Unrelated case

We first compute the probability that they simply match by chance, given that they are completely unrelated. This can be computed using coalescence theory. Assuming a mitochondrial effective population size of *N*, the distribution of pairwise coalescence times $p(t)$ follows an exponential distribution:

$$p(t) = \frac{1}{N} e^{-t/N}$$

Given a coalescence time *t,* the probability of a perfect sequence match equals the probability that no mutation has occurred since the common ancestor at time t. Since there are two independent branches on which mutations could have occurred, and given a sequence length L and a per-generation mutation rate μ, we have

$$p(match \mid t) = e^{-2L \mu t}$$

So the total probability that two randomly sampled mitochondrial sequences match perfectly is

$$p(match) = \int_0^\infty p(match \mid t)\, p(t) dt = \int_0^\infty e^{-2L\mu t} \tfrac{1}{N} e^{-t/N} dt = \tfrac{1}{N} \int_0^\infty e^{-(2L\mu + 1/N)t} dt$$
$$= (1 + 2LN\mu)^{-1}$$

An alternative way to derive this result is to consider both coalescence and mutation as competing Poisson processes. The rate of the combined process is

$$\tfrac{1}{N} + 2L\mu$$

And the probability that coalescence happens first is then simply

$$\tfrac{1}{N} / \left(\tfrac{1}{N} + 2L\mu\right)^{-1} = (1 + 2LN\mu)^{-1}.$$

For a sequence length of $L = 16569$ and a per-generation mutation rate of $\mu = 6.25 \times 10^{-7}$ (based on a generation time of 25 years and the per-year mutation rate estimated in [41]), we obtain:

- $p(match \mid N = 1000) = 0.046$
- $p(match \mid N = 10000) = 0.0048$
- $p(match \mid N = 100000) = 0.00048$

The mitochondrial effective population size is not easy to estimate in our case, but at least for autosomal effective population sizes, we know from [11] that most populations studied so far from the Bronze age and later have effective population sizes of at least 10,000 if not 100,000. The mitochondrial effective population size is likely different from that (larger with female exogamy, smaller with male exogamy). In fact, most evidence points to a generally larger effective population size in mitochondria than at least for Y-chromosomes [42], and most studied cases in the archaeogenetic record show evidence for female exogamy (for example [43,44]). There is evidence that this is the case also for the population studied here, given that out of 16 unrelated samples in our study, we find 16 different mitochondrial haplotypes. This corresponds to 120 possible pairs without a match and again consistent with a low unrelated matching rate of probably less than 0.01, and arguably closer towards 0.005 than 0.05.

Cryptic background relatedness

Apart from a chance match, which, as we see, gets increasingly unlikely given a presumably large effective population size, we should also consider the case of cryptic background relatedness. In the case of the parental model, where HOC001 would be APG001's father or vice versa, if APG001's parents would be first cousins, we could expect a mitochondrial match with probability $1/4$ (each has two grandmothers, and as first cousins, they share exactly one. The probability that the shared one is the maternal grandmother on both sides, is therefore $1/2 \times 1/2 = 1/4$). For second cousins it would be $1/4 \times 1/4 = 1/16$, for third cousins $1/64$ and for fourth cousins $1/256$.

In the case of the grandparental model, where HOC001 would be APG001's maternal grandfather, we would get a mitochondrial match if HOC001 and his female partner were

distant cousins, i.e. APG001's mother would be (lowly) inbred. Note that this is different from the paternal grandparental model, where there is no simple scenario of cryptic relatedness that could explain a match between HOC001 and APG001 in this case.

There is some evidence for a generally low inbreeding level in this population, given that we find low evidence of runs of homozygosity in many of our studied samples. Considering only the number of short fragments of runs of homozygosity of 4-8 cM, we find on average 1.95 (i.e. 2) such segments in our samples (some have zero, but others have more, see Supp. Table 2.5). According to simulations done in [11] (Figure S16), we can estimate an average parental relatedness of between 3rd and 4th cousins, so a chance of a cryptic mitochondrial match of between 1/64 and 1/256. We, therefore, consider

$$p(match) = 0.01$$

as a good estimate for the chance that an individual's two parents have the same mitochondrial sequence, which also agrees with the 120 pairs of unrelated mitochondria without a match, see argument above.

Updating the likelihoods given mitochondrial and autosomal evidence

We proceed by computing a table including the genetic likelihoods for the autosomal degree of relatedness by BREADR (see above), and the above-mentioned penalty of 0.01 (converted to log) for the models labelled inconsistent with MT/RoH, and a penalty of 0.005 for the paternal grandparental model (which has no easy scenario of cryptic background relatedness that could explain the mitochondrial match, so we are left with the coalescence-based probability), and compute a combined log likelihood for each model:

Pedigree	Marginal LLH	MT/ROH consistency	Genetic LLH	MT/ROH LLH	Combined LLH	Bayes Factor
H. is A.'s uncle	-5.6749	TRUE	-0.012073	0	-5.6869	1
H. is A.'s maternal grandfather	-3.6453	FALSE	-0.012073	-4.6052	-8.2625	0.076111
H. and A. are double first cousins	-8.8751	TRUE	-0.012073	0	-8.8871	0.040754
H. is A.'s paternal grandfather	-3.8585	FALSE	-0.012073	-5.2983	-9.1688	0.030749
A. and H. are half siblings	-10.178	TRUE	-0.012073	0	-10.19	0.011078
H. is A.'s father	-4.9888	FALSE	-4.4228	-4.6052	-14.017	0.0002
A. and H. are full siblings	-10.178	TRUE	-4.4228	0	-14.6	0.0001
A. is H.'s uncle	-14.95	TRUE	-0.012073	0	-14.962	0.00009
A. is H.'s father	-23.551	FALSE	-4.4228	-4.6052	-32.579	0
A. is H.'s maternal grandfather	-34.207	FALSE	-0.012073	-4.6052	-38.825	0
A. is H.'s paternal grandfather	-34.993	FALSE	-0.012073	-5.2983	-40.303	0

So taking everything together, the avuncular model where HOC001 is APG001's uncle is the most likely model, because it is the best model that fits the archaeological and anthropological data, as well as the genetic data including the MT-sharing and absence of

RoH in APG001. The second best model is the maternal grandfather model, where HOC001 would be the father of APG001's mother. This model explains the matching MT sequences via cryptic relatedness, which is unlikely (probability 0.01), but given the better fit of anthropological and archaeological data, this model still has a significantly non-zero Bayes factor (amounting to 6.6% of posterior probability, see below).

As a final step, we turn the combined Bayes Factors into a normalised posterior probability distribution over all 11 models:

**Pedigree**	**Combined LLH**	**Model Probability**
**H. is A.'s uncle**	-5.6869	0.863
**H. is A.'s maternal grandfather**	-8.2625	0.066
**H. and A. are double first cousins**	-8.8871	0.035
**H. is A.'s paternal grandfather**	-9.1688	0.027
**A. and H. are half siblings**	-10.19	0.009
**H. is A.'s father**	-14.017	0
**A. and H. are full siblings**	-14.6	0
**A. is H.'s uncle**	-14.962	0
**A. is H.'s father**	-32.579	0
**A. is H.'s maternal grandfather**	-38.825	0
**A. is H.'s paternal grandfather**	-40.303	0

Code availability

All code for the latent pedigree analyses can be accessed via a github repository: https://github.com/stschiff/celtic_relationship_analysis. The version of the code used in the published version of the paper can be accessed by a corresponding tag and release.

Supplementary Note 4: The formation of the Hallstatt Gene pool

General population genetic affinities

To assess the population genetic affinities of early Iron Age/Hallstatt period individuals from southwestern Germany, we performed principal component analysis, as described in *Methods*. For the *European PCA*, we used the following present-day 36 populations to calculate the principal components: Norway, Sweden, Denmark, Germany, Netherlands, Belgium, Ireland, Scotland, Wales, England, France, Spain, Portugal, Italy, Finland, Russia, Belarus, Estonia, Latvia, Lithuania, Poland, Ukraine, Slovakia, Hungary, Slovenia, Romania, Bulgaria, Croatia, Serbia, Kosovo, Bosnia, North Macedonia, Montenegro, Albania, Greece, and Italy (Supp. Fig. 4.1a). We note that PC1 and PC2 of this PCA setup are highly correlated with geography, with PC1 corresponding to a north-to-south gradient and PC2 corresponding to an east-to-west gradient.

In general, the *European PCA* implies high genetic similarity between the Hallstatt genomes from southwestern Germany and i) present-day genomes from France (Supp. Fig. 4.1b) as well as ii) Middle Bronze Age genomes from the Bavarian Lech valley in southern Germany and Iron Age genomes from France and the Czech Republic (Supp. Fig. 4.2). This strong genetic affinity between Hallstatt individuals from southwestern Germany and present-day French can also be observed in formal F_4 and F_{ST} statistics, as described in Supplementary Note 5. However, one individual distinctively deviates from the main cluster. The La Tène period LAN001 individual from Alte Burg clusters instead together with present-day individuals from the Netherlands, Denmark, and Norway, as well as Bronze Age and Iron Age individuals from these regions.

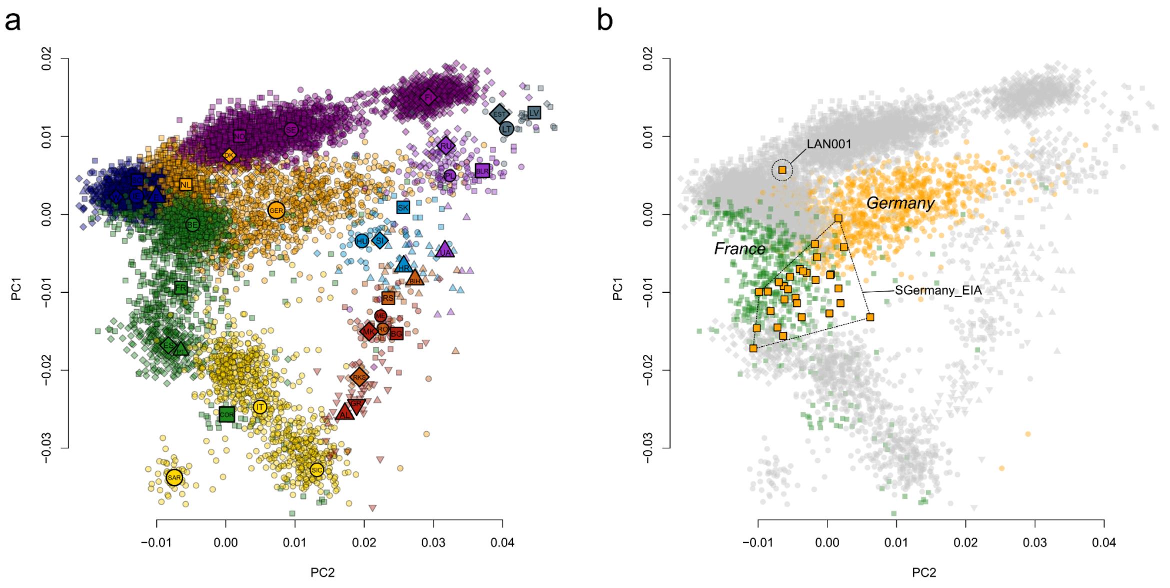

Supplementary Figure 4.1. Principal Component Analysis of European variation. a) Principal Components Analysis of present-day genomes from Europe. IE = Northern Ireland & Ireland, WA = Wales, SC = Scotland, E = England, NL = Netherlands, GER = Northern Germany, DK = Denmark, NO = Norway, SE = Sweden, BE =

Belgium, FR = France, ES = Spain, PT = Portugal, COR = Corsica, IT = Italy, SIC = Sicily, SAR = Sardinia, FI = Finland, EST = Estonia, LV = Latvia, LT = Lithuania, RU = Russia, BLR = Belarus, PL = Poland, SK = Slovakia, HU = Hungary, SL = Slovenia, UA = Ukraine, HR = Croatia, BIH = Bosnia, RS = Serbia, ME = Montenegro, RO = Romania, MK = North Macedonia, BG = Bulgaria, RKS = Kosovo, GR = Greece, AL = Albania. **b)** Genetic structure of novel ancient individuals (depicted as orange squares) in this study, projected onto a).

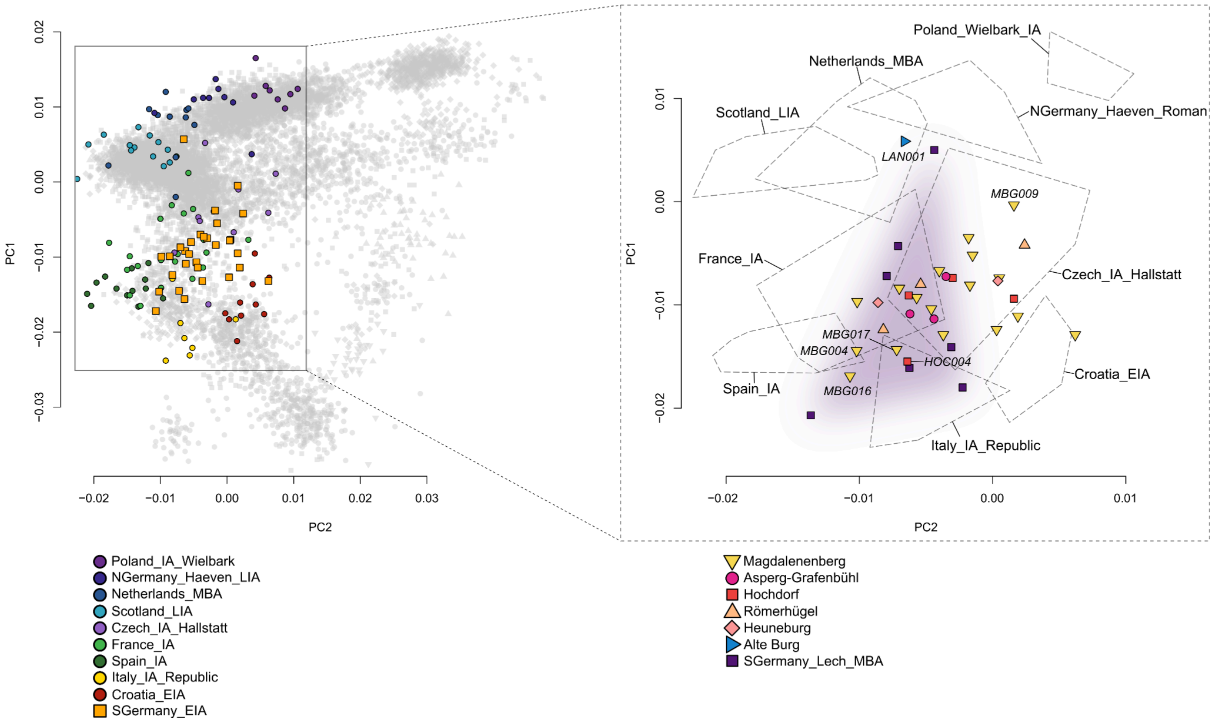

Supplementary Figure 4.2. Principal Component Analysis of European variation and ancient population structure. Genetic structure of published and novel ancient individuals in this study, projected onto figure 4.1. The left plot shows several Iron Age individuals plotted together with our novel samples from early Iron Age southern Germany. In the right plot, the ancient reference groups are only shown as convex hulls for better visibility. Novel Iron Age and previously published Middle Bronze Age samples from southern Germany are represented with individual symbols. The symbols and colours correspond to Figure 1. Outlier individuals mentioned in the main text are labelled.

To further infer the population genetic affinities of the early Celtic population (excluding the La Tène period outlier LAN001), we compared it to several published samples from Europe using different *F*-statistics[45]. We first calculate i) shared drift from an outgroup via outgroup F_3 statistics between *SGermany_EIA* and contemporaneous ancient populations, ii) genetic differentiation via F_{ST}[46] between *SGermany_EIA* and contemporaneous ancient populations, and iii) symmetry tests via F_4 statistics between ancient and present-day populations. We computed the outgroup F_3 and F_4 statistics in ADMIXTOOLS[45] applying the programs *qp3Pop* (with the option *inbreed* set to YES) and *qpDstats* (with *f4mode* set to YES), and used Yoruba in Ibadan, Nigeria (YRI.SG) as outgroup population in F_3 statistics and Han Chinese from Beijing (CHB.SG) as outgroup population in F_4 statistics.

For the F_3 statistics, the results are reported in Supplementary Tables 3.1-3. The highest fractions of shared genetic drift were measured between *SGermany_EIA* and Bronze Age samples from Sardinia (probably due to high percentages of *EEF* ancestry in *SGermany_EIA*), Bronze Age samples from southern Germany, as well as Iron Age samples from Slovenia, Switzerland, and the Czech Republic, suggesting that the strong cultural links

between those regions were also reflected in the ancestry of those groups. In contrast, the northern European outlier LAN001 (dating to the Late Iron Age La Tène period) shows the highest genetic similarity to Bronze Age, Iron Age, and early medieval individuals from the Netherlands, northern Germany, Denmark, and Sweden (Supp. Table 3.3).

For F_{ST}, the results are reported in Supp. Tables 3.4-5. Lowest genetic differentiation was observed between *SGermany_EIA* and Iron Age samples from northern France, Central Germany (the Late Iron Age/Roman period Haßleben individuals), the Czech Republic, Austria, Hungary, and Slovakia, which is consistent with the results obtained using outgroup F_3 statistics.

For the F_4 statistics, we calculated for 86 Bronze Age, Iron Age, and medieval populations all possible combinations of the *F*-statistic F_4(CHB, X; *TestA*, *TestB*). *TestA* and *TestB* iterate through the following 16 present-day European populations:

- Balkan Peninsula (Bosnia, Bulgaria, Montenegro, North Macedonia, Romania, Serbia)
- Basque.DG
- Bedouin.DG
- Belgium
- Denmark
- Finland
- France
- Germany
- Ireland
- Italy
- Netherlands
- Poland
- Sardinian.DG
- Spain
- Sweden
- Yoruba.DG

We extracted all unique combinations per ancient population X and then performed PCA on the computed F_4 statistics (Supp. Fig. 4.3, Supp. Table 3.6). We note that in this PCA setup the positions of the ancient populations along PC1 and PC2 strongly resemble the geographical origin of the tested populations. Similarly to the PCA calculated on individual present-day genotype data, we highlight that the *SGermany_EIA* population plots between Bronze and Iron Age individuals from France and the Czech Republic as well as individuals from central-eastern Europe, especially Hungary and Slovenia, indicating an intermediate position of the Hallstatt population of southwestern Germany between those groups.

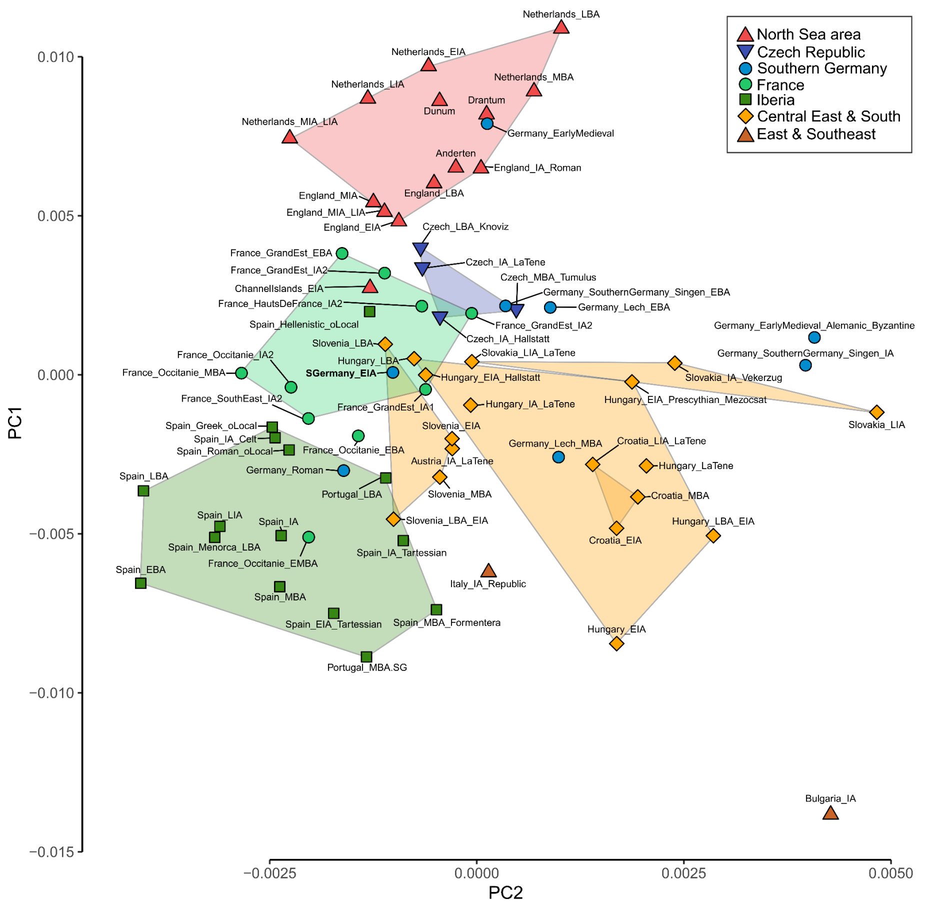

Supplementary Figure 4.3. Principal Component Analysis of F_4 statistics. PCA of F_4 statistics of the form F_4(CHB, ancient population; TestA, TestB). TestA and TestB iterate through 15 present-day European populations (Methods).

Supervised ADMIXTURE modelling

We proceeded to decompose the ancestry of the early Celtic individuals applying supervised model-based clustering implemented in ADMIXTURE[14]. For that, we followed the approach described in Gretzinger et al 2022[5]. We initially used an unsupervised approach to identify clusters in 9,213 present-day Europeans. We then selected the present-day populations in which a component was maximised to represent this cluster as source within the supervised ADMIXTURE setup. Those components are:

- $FIN_{Finnish}$ (maximised in Finns; *n* = 606)
- $CNE_{Continental\ North\ European}$ (maximised in Danes, northern Germans, and Dutch; *n* = 905)
- NOR_{Norse} (maximised in Swedes and Norwegians; *n* = 1910)
- $WBI_{Western\ British\ Irish}$ (maximised in Irish, northern Irish, Scottish, and Welsh; *n* = 667)
- $CWE_{Continental\ Western\ European}$ (maximised in Spanish and French; *n* = 812)
- BAL_{Baltic} (maximised in Russians, Belarussians, Latvians, Lithuanians, and Polish; *n* = 167)

We further added the following non-European sources in order to also detect Asian and African genetic variation:

- $WAS_{West\ Asian}$ (represented by Greek, Cypriot, Turkish, Druze, and Assyrians; *n* = 67)
- $NEA_{Middle\ Eastern}$ (represented by Bedouins, Palestinians and Saudi; *n* = 121)
- $AFR_{African}$ (represented by Esan, Mende, and Yoruba; *n* = 300)

- $SAS_{South\ Asian}$ (represented by Punjabi, Gujaratis, Brahui, and Balochi; *n* = 286)
- $EAS_{East\ Asian}$ (represented by Han Chinese and Japanese; *n* = 207)
- $NAS_{North\ Asian}$ (represented by Yakut and Hezhen; *n* = 28)

Subsequently, we modelled 5,142 ancient European and West Asian individuals, dating between the Late Neolithic (5,000 BP) and the Modern Period (150 BP), at *K* = 12 (Supp. Table 3.7).

As expected ancient British-Irish Islanders show the largest WBI component, reflecting highest genetic affinity to present-day British Irish-Islanders (Supp. Table 3.7) (e.g. Scotland_Shetland_IA.SG: 99%, Wales_LBA: 89%,Ireland_EBA.SG: 89%). Similarly, ancient individuals from the Baltics exhibit the highest fractions of BAL ancestry (Lithuania_Bailuliai_BarrowCulture.SG: 99%, Latvia_BA: 99%, Estonia_BA.SG: 96%). The NOR component is maximised in Bronze and Iron Age samples from Norway and Sweden (Sweden_IA_2.SG: 99%, Norway_IA: 95%, Sweden_Late_N.SG: 92%), while Bronze and Iron Age samples from present-day Denmark appear as mixture between the NOR and the CNE components, probably reflecting their intermediate positions between those two poles of ancestry. The CNE component is maximised in Iron Age and early mediaeval samples from present-day northern Germany (NGermany_Haeven_Roman: 71% and NGermany_EMA: 78%). Similarly, Bronze and Iron Age individuals from present-day Netherlands and central Germany carry mostly WBI and CNE ancestry, congruent with their geographical origin.

Interestingly, Bronze and Iron Age individuals from western and central Europe are dominated by the CWE component, maximised in ancient Iberians and Sardinians. In Iron Age samples from present-day eastern and northern France, southern Germany, the Czech Republic, and Slovenia, CWE ancestry is the largest component, however, we also detect BAL, WBI, CNE and WAS ancestry in these individuals, probably indicative of admixture with neighbouring northern, northeastern, and southeastern populations. For example, we highlight that the Slovakian Iron Age Vekerzug and Hungarian Iron Age Mezőcsát individuals show especially high percentages of eastern European BAL ancestry (34% and 28%, respectively), consistent with the eastern material influences in those two cultures[47]. In general, the supervised ADMIXTURE results support the results of PCA, F_4, and F_{ST} statistics, indicating a strong genetic similarity between Hallstatt and La Tène individuals from western and central Europe and present-day Spanish and French.
In general, we do not find one homogeneous gene pool that covers the whole geographical area that is associated with the Celtic languages and the spread of the Hallstatt and La Tène cultures. While the CWE component is the largest ancestry component in most populations that inhabited these regions from the Middle Bronze Age to the end of the Iron Age and Roman period, indicating a shared biological background for all those groups, we however observe strong genetic variation that is structured geographically (Figure 3).

Pairwise qpWave testing

To directly test genetic similarity between ancient groups, we tested on the population level whether the *SGermany_EIA* individuals were consistent with forming a clade with 105 ancient European populations using qpWave[12,13] from ADMIXTOOLS[45] (v.4.1) (Supp. Table 4.10). We set the option allsnps: YES to specify that the F_4 statistics, which are the basis of qpWave analyses, should be computed using the union of SNPs with coverage in all four

groups that contribute to each F_4 statistic. We used a set of 11 Outgroups in the following order: YRI.SG, Poland, Finland, Sweden, Denmark, Ireland, Wales, Italy, Spain, Belgium, Netherlands (see Methods, set I). Of 92 tested populations, seven are genetically indistinguishable from the *SGermany_EIA* population, namely *France_GrandEst_IA1.SG, France_GrandEst_IA2, Slovakia_LIA_LaTene, France_HautsDeFrance_IA2.SG, Czech_IA_Hallstatt, Germany_Lech_MBA*, and *Germany_Hassleben_Germanic.SG.* Three (43%) of those populations are located in France, two (29%) in Germany, one in the Czech Republic (14%) and one in Slovakia (14%), dating between the Middle Bronze Age and the Late Iron Age/Roman period. The results are shown in Figure 4b.

qpAdm modelling using distal sources

So far, we have shown that there is strong genetic similarity between Hallstatt and La Tène groups from western and central Europe, but also between those ancient groups and present-day western Europeans, especially French, Spanish, and Belgians. To reconstruct how the Iron Age Hallstatt gene pool of southwestern Germany has formed and how it is ancestrally related to the present-day populations of western Europe, we reproduced the approach described in Patterson et al. 2021[48] of decomposing the ancestry of ancient individuals into three ancestral sources (Balkan Neolithic farmers with minimal hunter–gatherer admixture (*EEF*), Yamnaya and Poltavka pastoralists (*OldSteppe*), and 18 Mesolithic hunter–gatherers from across western Europe (*WHG*)), using the same set of right and outgroup populations (see Methods, set II). We calculated ancient admixture profiles using two different setups: i) for each individual separately (Supp. Fig. 4.4, Supp. Table 3.8) and ii) for individuals pooled together as populations (Supp. Figure 4.5, Supp. Table 3.9, 4.1 & 4.2). For setup ii), we compared population-based estimates of EEF, Steppe, and WHG ancestry estimates among 153 Bronze Age, Iron Age, early medieval and present-day groups using PCA (Supp. Figure 5.2).

Similar to the results of Patterson et al. 2021 for prehistoric Britain, we observe a marked increase of *EEF* ancestry in southwestern Germany during the Bronze Age, increasing from 44.1% ± 0.7% during the Early Bronze Age to 49.6% ± 0.6% during the early Iron Age, which is consistent with prolonged admixture with a southern European source that experienced less gene flow from steppe-related populations, at least until the Middle Bronze Age (53.6% ± 1.2%) (Supp. Fig. 4.4c, Supp. Fig. 4.5a). The continuous nature of this admixture process was tested using DATES[49]. We observe that admixture time decreases significantly with the date of each individual (Spearman's rank correlation, p = 2.98e-7). Moreover, the slope of this decrease is close to 1.0 (0.75 ± 0.13), a result incompatible with a single pulse of admixture, but compatible with stationary continuous and ongoing admixture (Supp. Fig. 4.5d, Supp. Table 3.11). The same pattern of increasing affinity to *EEF* is also observed when calculating individual F_4 statistics of the form F_4(YRI, Test, Steppe, *EEF*) (Supp. Fig. 4.4a, Supp. Table 3.10). Additionally, we also observe a reduction in F_4 and EEF point estimate variances between the time periods, especially between the Middle Bronze Age and the Iron Age, indicating a homogenisation of the gene pool in terms of *EEF* ancestry (Supp. Fig. 4.4b & d). Consequently, as shown in Supplementary Figure 4.5a, the *EEF* ancestry proportions of France and southern Germany are nearly identical during the Iron Age. Correspondingly, *Steppe* ancestry decreased from the Early Bronze Age 43.5% ± 0.8%) to the Middle Bronze and Iron Age (34.6% ± 1.4% and 36.4% ± 0.7%) (Supp. Fig.

4.5b). To investigate the source of this *EEF*-enriched ancestry, we applied qpAdm and tested an two-way admixture model for *SGermany_EIA*, where on source is defined to be *Germany_Lech_EBA* and the second source iterates through 109 prehistoric and historic populations from Europe, especially covering central, southern, southwestern and western European populations. We note that most groups that produce a fitting *p*-value ($p > 0.01$) are either located in France or on the Iberian or Italian peninsulas, suggesting an southwestern European origin of the introgressing *EEF*-enriched ancestry (Supp. Table 3.12).

In contrast, with the beginning of the Early Middle Ages, we note a drop in *EEF* ancestry (to 38.9% ± 0.6%) that most likely reflects the introduction of *Steppe*- and *WHG*-enriched CNE and NOR ancestry (as seen in the *SGermany_EMA* population). During this time, the population of southwestern Germany closely resembles Iron Age and early medieval populations from northern Germany and Scandinavia, exhibiting elevated levels of *WHG* (14% ± 0.6%) and *Steppe* (47.1% ± 0.8%) ancestry (Supp. Fig.4.5b), reflecting the demographic changes illustrated with PCA, F_4, F_{ST}, and ADMIXTURE analyses. However, when analysing present-day Germans, we infer that they on the other hand harbour on average higher levels of *EEF* ancestry (41.5% ± 0.4%) and lower levels of Steppe (44.5% ± 0.5%) than the population of the preceding Early Middle Ages, suggesting a resurgence of *EEF*-enriched ancestry.

In the wider context of the European Iron Age gene pool, we observe the same pattern as inferred using supervised ADMIXTURE. While there is strong genetic resemblance between Hallstatt and La Tène groups in western Europe in terms of their ancestral ancestry components, there is strong regional variation associated with geography (Supp. Fig. 4.5c). *Steppe* ancestry is significantly associated with latitude and longitude (Spearman's rank correlation; $S = 1831380$, $p < 2.2e\text{-}16$ and $S = 3404746$, $p = 0.000722$), whereas *WHG* ancestry is correlated with longitude (Spearman's rank correlation; $S = 5483160$, $p = 2.676e\text{-}07$) but not with latitude (Spearman's rank correlation; $S = 4380682$, $p = 0.5578$). Thus, we can infer that the groups inhabiting the borders of the Hallstatt and later La Tène cultural spheres were admixing with neighbouring populations, and in general, exhibited population structure that followed geography. In the wider European context, *Steppe* ancestry peaks in the north (Supp. Fig. 4.5c), especially in Scandinavia where *Steppe* ancestry does not increase north of latitude 54.5°N ± 1.3, mirroring the distribution of CNE and NOR ancestry (which is potentially associated with the distribution of Germanic-speaking groups). *WHG* ancestry is found in the highest proportions on the one hand in southern France and eastern Spain, and on the other hand along the Baltic shore as well as in Hungary and the Czech Republic where we measure the highest fractions of BAL ancestry. EEF ancestry is most prominently found in Iberia, Italy, and the Balkans, following the distribution CWE and WAS ancestry.

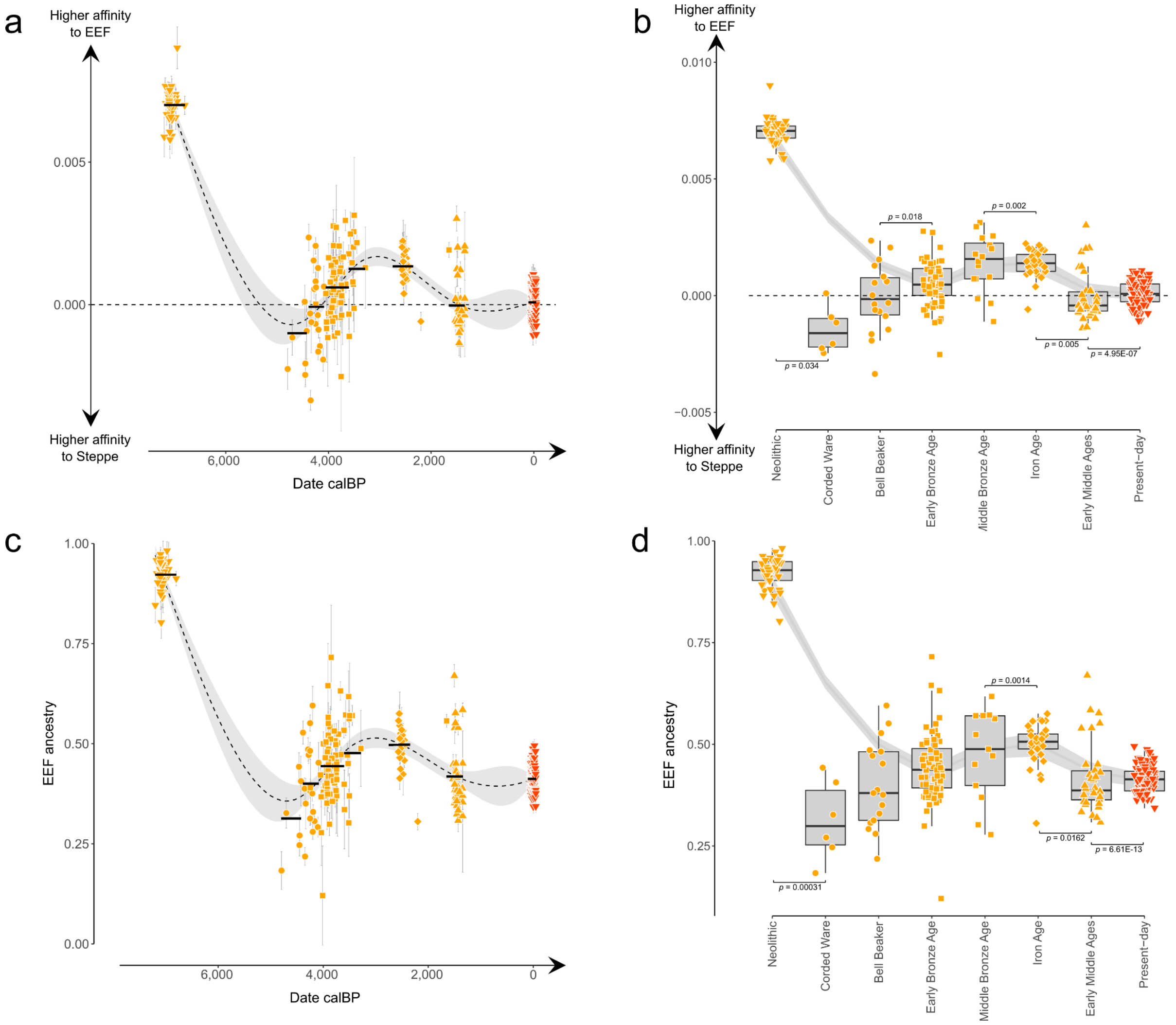

Supplementary Figure 4.4. Changes in affinity to Early European Farmers. a) F_4 statistic of the form F_4 (YRI, Test; Steppe, EEF) for ancient and present-day individuals from southern Germany (n = 319). Shown is the Loess Regression (and its 95% CI) and the mean per period (Early Neolithic, Corded Ware period, Bell Beaker period, EBA, MBA, IA, EMA, and present-day) in black. Point estimates for ancient genomes (n = 219) are depicted in orange, present-day genomes in red (n = 100). Error bars denote one standard error. **b)** Distribution of F_4 statistics of the form F_4(YRI, Test; Steppe, EEF) in southern Germany from the early Neolithic to the present-day. Sample sizes are 45, 6, 17, 68, 13, 31, 38 and 100 for the Neolithic, Corded Ware, Bell Beaker, EBA, MBA, IA, EMA, and present-day period, respectively. Shown are the significant p-values from pairwise two-sided F-tests. Bounds of the Box represent the 25th and 75th Percentile. The centre represents the median. Whiskers represent the smallest value greater than the 25th Percentile minus 1.5 times the interquartile range and largest value less than the 75th Percentile plus 1.5 times the interquartile range, respectively. Outliers present the minimum and maximum values in the data. **c)** Same as a) for Early European Farmer ancestry as inferred using qpAdm in ancient and present-day southern German individuals (n = 319). **d)** same as b) for the distribution of Early European Farmer ancestry in present-day southern Germany from the early Neolithic to the present-day. Sample sizes are the same as in panel b). Error bars denote one standard error.

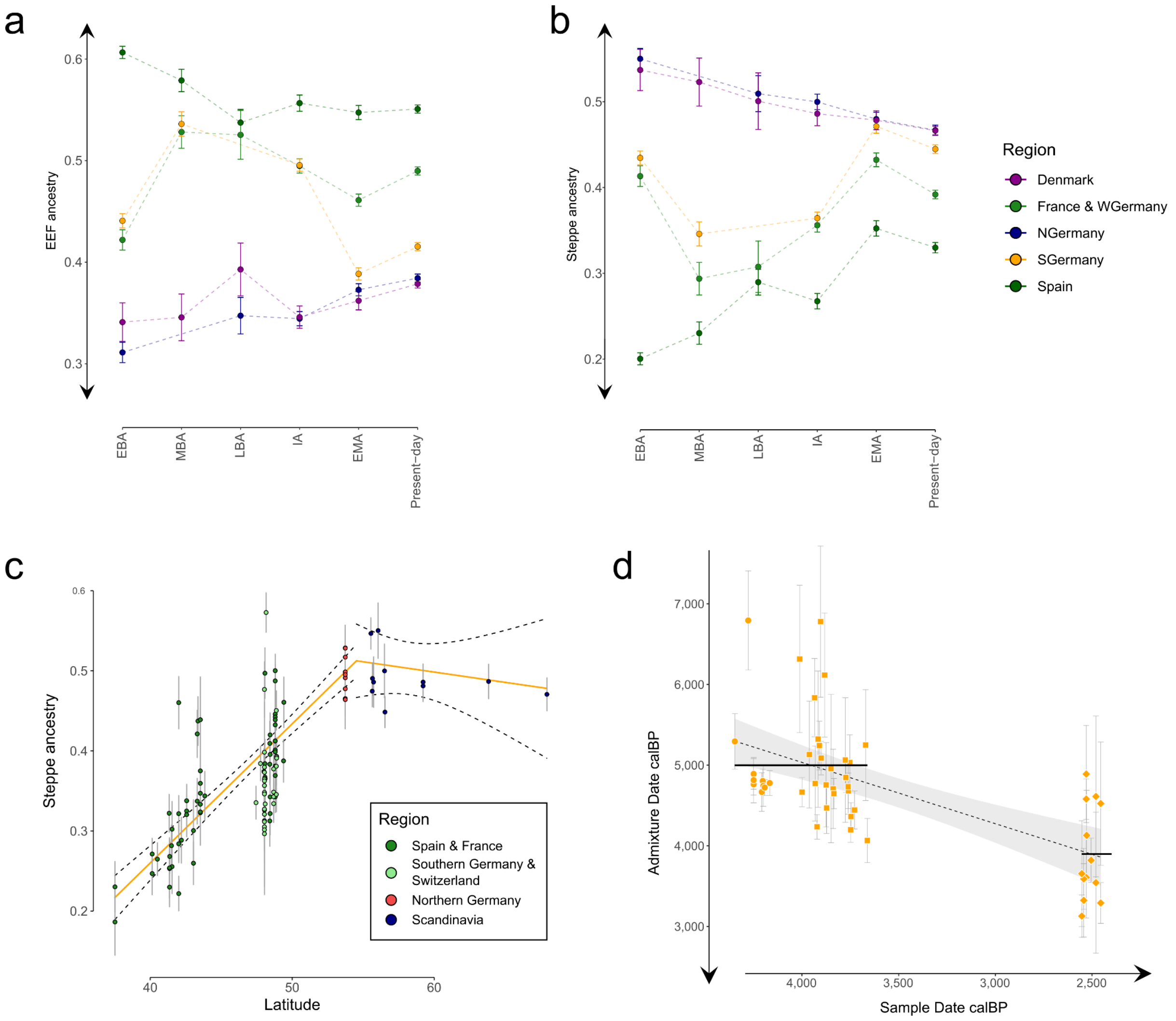

Supplementary Figure 4.5. Genetic differentiation between northern and southern Europe. a) Point estimates of EEF ancestry for Bronze, Iron Age, early medieval and present-day populations from five European regions fitting a three-way admixture model (EEF + WHG + Steppe) using *qpAdm*. Error bars denote one standard error. **b)** same as a) for *Steppe* ancestry. **c)** Point estimates for the proportion of Steppe ancestry along latitude in Iron Age individuals (*n* = 112) from Spain and France (*n* = 60), Switzerland and southern Germany (*n* = 32), northern Germany (*n* = 9), Denmark, Sweden, and Norway (*n* = 11) modelled as a two-stage linear process. Error bars denote one standard error. The dotted lines indicate the 95% confidence interval. **d)** Chronology of admixture between EEF and Steppe sources. Shown are the estimated admixture dates (in years before present) between EEF and Steppe sources in Bronze and Iron Age individuals from southern Germany (*n* = 51) as inferred using DATES. Error bars of point estimates denote one standard error. The dotted line indicates the 95% confidence interval of the linear regression

Supplementary Note 5: Population genetic changes after the Hallstatt period

The end of the Hallstatt gene pool in southern Germany

The aforementioned substantial changes in *EEF*, *Steppe*, and *WHG* ancestry profoundly changed the population genetic affinities of the post-Neolithic groups inhabiting present-day southern Germany. The initial rise of *EEF* ancestry and its abrupt decrease are also visible in the affinity to present-day populations. To demonstrate these changes, we calculated F_4 statistics of the form F_4(CHB, TestA; Ireland, TestB), where TestA iterates through 18 ancient and one present-day group from southern and central Germany and TestB iterates through 12 present-day European populations (Supp. Fig. 5.1, Supp. Table 4.2). Present-day Ireland was used for comparison due to its high proportion of Steppe and low amount of WHG ancestry. Additionally, we assume that most ancient samples from Germany should be, depending on their ancestry, closer related to either continental southern European or continental Northern European, highlighting changes in the local ancestry due to influx from the South or the North.

The early Neolithic LBK population from Stuttgart-Mühlhausen[50] is closest related to present-day Italians, reflecting the high proportions of *EEF* ancestry[51,52], which today peak in Italy, especially Sardinia[53]. The later Middle Neolithic individuals from Salzmünde[54], Baalberge[55], and Blatterhöhle[54], however, show higher genetic affinity to present-day Spanish, which might be explained by the resurgence of *WHG* ancestry in those individuals due to gene flow from hunter-gatherers after the LBK period[13,50,54].Today, higher percentages *WHG* ancestry are found in Spanish than in Italians, potentially causing the observed attraction of the Middle Neolithic individuals (Supp. Table 4.1).

In contrast, during the Late Neolithic, the Corded Ware and Bell Beaker periods, as well as the Early Bronze Age, no present-day population is significantly closer related to those ancient groups than present-day Irish, although Scottish and Welsh are equally close. This corresponds to the introduction of *Steppe* ancestry, which is today found in the highest frequencies in northern Europe, especially in Ireland and western Scotland (Supp. Table 4.1). Corresponding to the increase of *EEF* ancestry from the Early Bronze Age onwards, the genetic affinity of the southern German population changes again, exhibiting the strongest genetic affinity present-day Spanish, consistent with the predominance of CWE ancestry during the Hallstatt period as inferred using supervised ADMIXTURE in Supplementary Note 4.

The affinity towards present-day Spanish is preserved during the Roman period until the beginning of the Early Middle Ages. Samples from several sites in Bavaria[56] and Niederstotzingen[57] exhibit significant attraction to present-day Dutch, northern Germans, and Danish, a pattern that is maintained in the present-day German population. This is associated with a substantial increase in both *Steppe* and *WHG* ancestry during the Early Middle Ages, indicative of migrations from northern Germany and southern Scandinavia

introducing (*Steppe*- and *WHG*-enriched) CNE ancestry to southern Germany. Applying the MOBEST approach described in Supplementary Note 2 to the early medieval samples from southern Germany, consequently places the predicted origin area of those individuals along the North Sea and Baltic Sea shore, in present-day Lower Saxony and Schleswig-Holstein (Supp. Fig. 5.5).

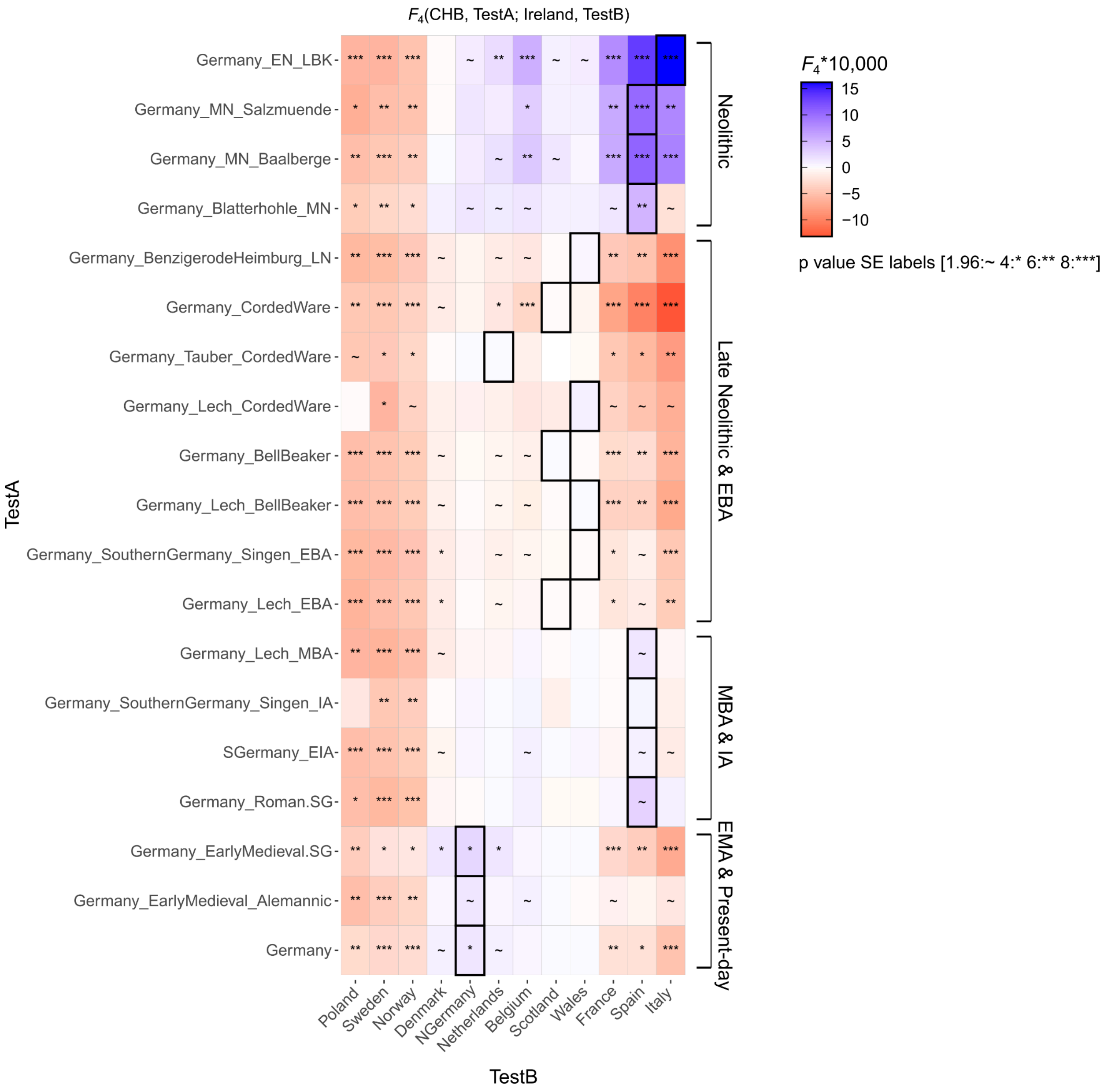

Supplementary Figure 5.1. Changes in affinity to present-day European populations through time. Shown are *F*-statistics of the form F_4(CHB, TestA; Ireland, TestB). TestA iterates through 18 ancient and one present-day southern and central German population. Negative values indicate that the TestA population is closer related to Ireland than to TestB; positive values indicate that the TestA population is closer related to TestB than to Ireland.

In contrast to southern Germany (Supp. Fig. 5.2b), northern Germany was less affected by the resurgence of *EEF* ancestry after the end of the Neolithic (Supp. Fig. 5.2c, Supp. Table 4.3). While we observe a general increase of *EEF* ancestry, from 31.1% ± 1% during the

Early Bronze Age to 34.4% ± 0.7% during the Late Iron Age/Roman period, this is far less pronounced than the increase detected in southern Germany (Supp. Fig. 4.5a). We further note an increase in *WHG* ancestry, resulting in strong genetic similarity between the Iron Age populations of northern Germany, Denmark, and Scandinavia (Supp. Fig. 5.2c). Such a *Steppe*- and *WHG*-enriched population must have transmitted ancestry from the Baltic Sea area to southern Germany, lastingly impacting the local population.

Similarly to southern Germany, we observed such a sequence of increase and decrease of *EFF* ancestry also in individuals from the present-day Czech Republic[48] (Supp. Fig. 5.2d, Supp. Table 4.3). During the EBA, the *EEF* proportion of individuals from present-day Czech Republic (31.8%) is even lower than the proportion in individuals from present-day southern Germany (44.1%), but increases to 43.9% during the Hallstatt period of the Iron Age (peaking during the Middle Bronze Age tumulus culture with 45%). During the early Middle Ages, the *EEF* fraction is then again reduced to pre-EBA levels, with one individual exhibiting 31% *EEF* ancestry, but 46.7% *Steppe* ancestry (the highest percentage since the Early Bronze Age), as well as the highest *WHG* ancestry proportion measured in ancient individuals from Czech Republic (22.3%). As in southern Germany, this change can only be explained by the introduction of new ancestries enriched in *Steppe* and *WHG* ancestry. However, the ratio of *WHG* to *Steppe* ancestry in the medieval individuals from Czech Republic (47.8) is much higher than in the early medieval individuals from southern Germany (0.314). This indicates that the rise in *Steppe* and *WHG* ancestries in those two regions was caused by different sources of ancestry. The source for the *Steppe*- and *WHG*-enriched ancestry that entered the region of present-day Czech Republic during the Early Middle Age (probably in form of the Slavic expansion) is most likely located in northeastern Europe, where higher percentages of *WHG* ancestry are found than in Scandinavia and other parts of northwestern Europe. Similar proportions and ratios of *Steppe* and *WHG* ancestry are found in Bronze Age individuals from present-day Latvia (0.51), Iron Age (0.441) and Medieval individuals from present-day Estonia (0.453), and Late Bronze Age individuals from present-day Lithuania (0.417).

We can estimate the impact of this gene flow event using the supervised ADMIXTURE approach described in Supplementary Section 4. If we use the CNE and NOR components as proxy for the incoming northern European ancestry, we estimate an increase of median CNE + NOR ancestry from 2.8% during the Iron Age to 62.5% during the Early Middle Ages (Supp. Fig. 5.3). The introduction of substantial fractions of northern Europeans ancestry is then also visible using F_{ST} and F_4 statistics: While the Hallstatt population of Iron Age southern Germany exhibits highest genetic affinity to present-day French, Belgians, Spanish, and English (Supp. Fig. 5.4a, Supp. Table 4.4 & 4.6), the early medieval population shows strongest attraction to present-day northern Germans, Dutch, Danish, and Scandinavians (Supp. Fig. 5.4b, Supp. Table 4.5 & 4.7).

We used qpWave with the basic set of outgroups described in *Methods* and Supplementary Note 4 to formally test for genetic continuity (Supp. Fig. 5.6, Supp. Table 4.9). We note that Middle Bronze Age samples from the Lech valley, Iron Age samples from Baden-Württemberg, and one Roman period individual from Bavaria are genetically indistinguishable, indicating genetic continuity during this period of time. However, none of those groups can be modelled as forming a clade with early medieval samples from Bavaria

and Baden-Württmeberg, who are in turn genetically indistinguishable from present-day northern Germans, highlighting the northern European origin of the incoming ancestry.

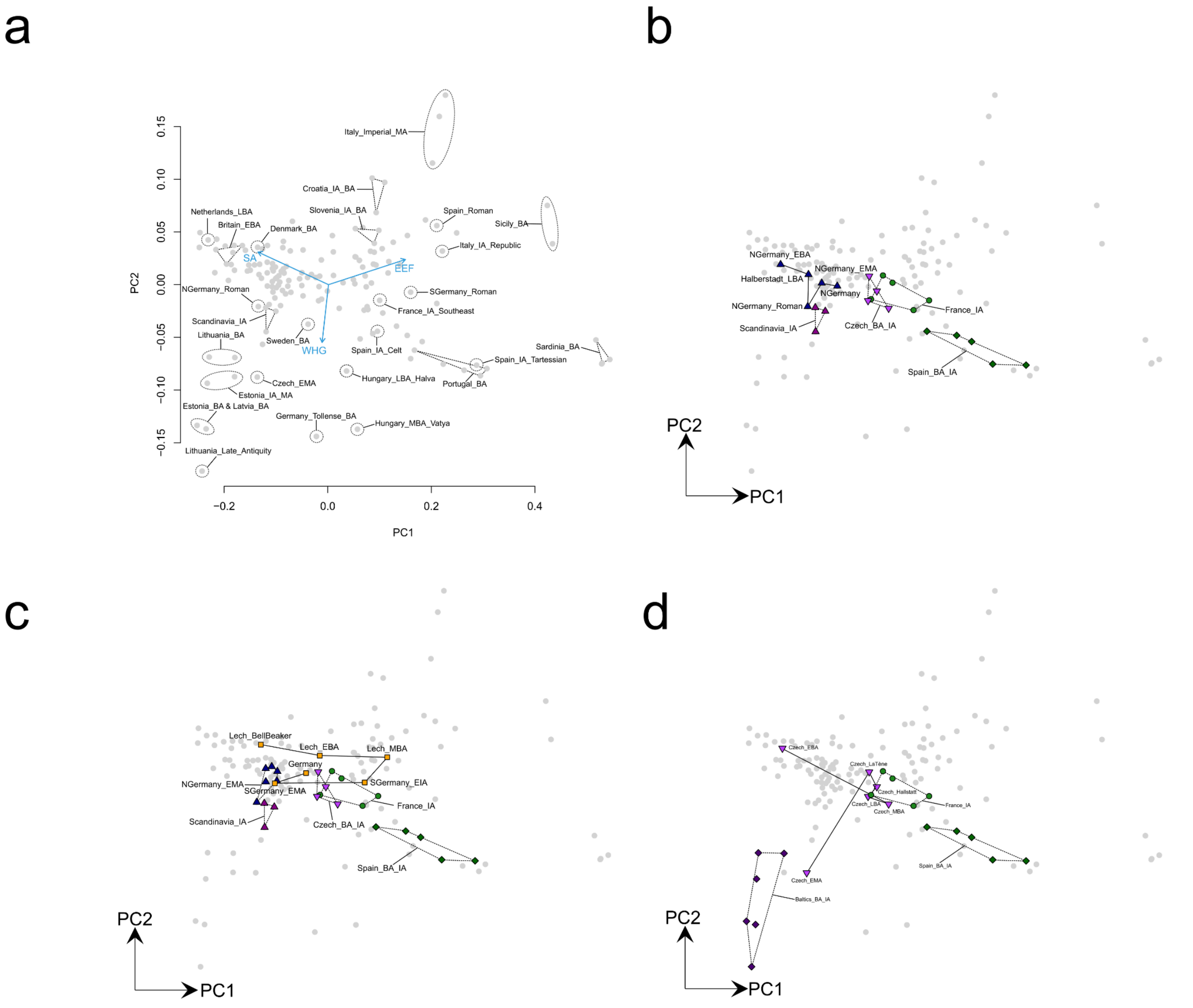

Supplementary Figure 5.2. Changes of ancestral ancestry components through time. Shown is a PCA of WHG, EEF, and Steppe ancestry estimates for 153 ancient and present-day populations from Europe. **a)** Several relevant groups are highlighted for reference. **b)** Changes in ancestral admixture components highlighted for southern Germany. **c)** same for northern Germany. **d)** same for the Czech Republic.

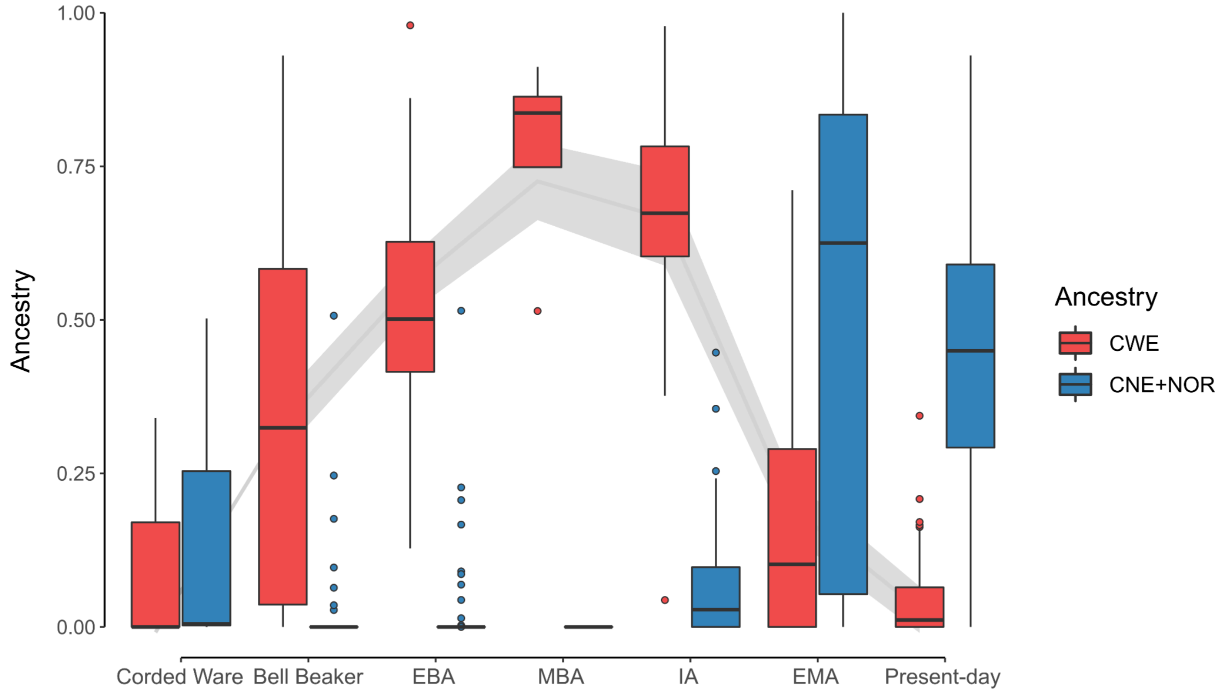

Supplementary Figure 5.3. Changes of northern European ancestry in southern Germany through time. Boxplots depdict individual estimates of CNE + NOR ancestry from supervised ADMIXTURE for seven post-Neolithic periods. Bounds of the Box represent the 25th and 75th Percentile. The centre represents the median. Whiskers represent the smallest value greater than the 25th Percentile minus 1.5 times the interquartile range and largest value less than the 75th Percentile plus 1.5 times the interquartile range, respectively. Outliers present the minimum and maximum values in the data. Sample sizes are 6, 17, 68, 13, 31, 38 and 100 for the Corded Ware, Bell Beaker, EBA, MBA, IA, EMA, and present-day period, respectively.

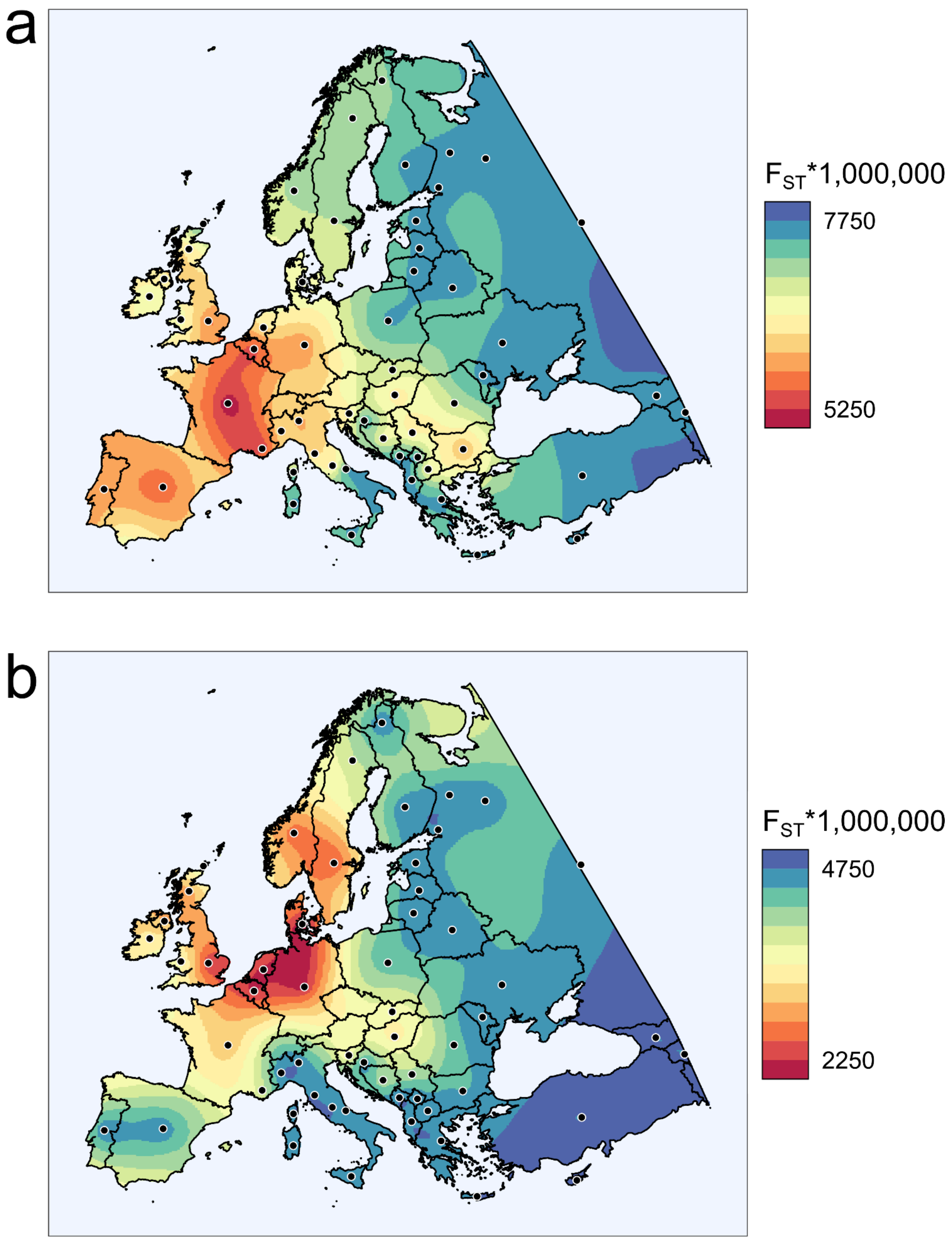

Supplementary Figure 5.4. Genetic affinities of groups from present-day southern Germany in the Iron Age and the early Middle Ages. a) Interpolation of F_{ST} calculated between SGermany_EIA and 58 present-day European groups. **b)** same for SGermany_EMA.

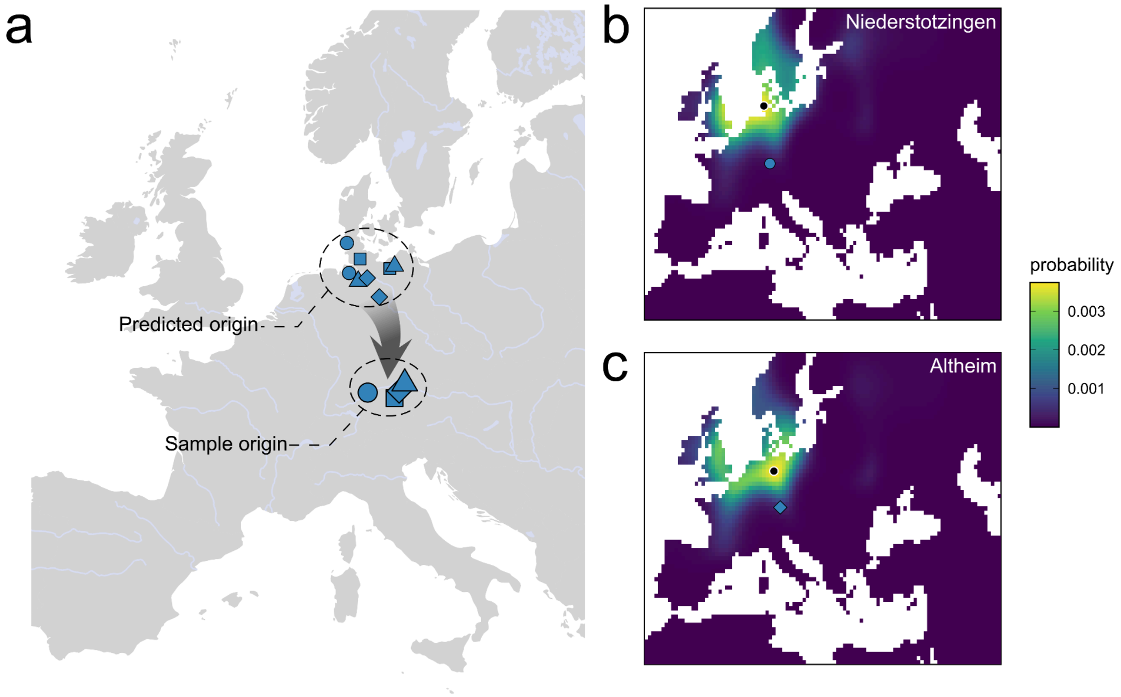

Supplementary Figure 5.5. Spatial inferences on the origin of early medieval individuals. a) MOBEST predictions of the geographic regions where the ancestors of early medieval individuals(CNE+NOR ancestry > 95%) from present-day southern Germany (Bavaria & Baden-Württemberg) originated. Shown are the points of maximum probability, symbols and colours correspond to Figure 1. **b)** Genetic similarity probability map for an individual from Niederstotzingen (NIEcap12b) [57] (Baden-Württemberg). The filled shape shows the burial location, and the black dot the point of maximum probability. **c)** same as b) for an individual from Altheim (ALH_1) [56] (Bavaria).

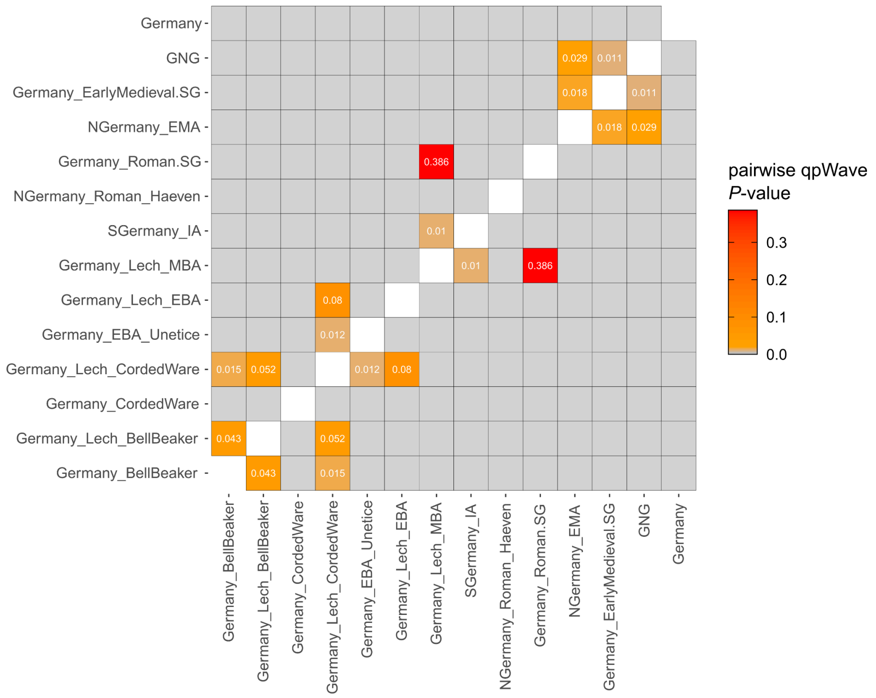

Supplementary Figure 5.6. Genetic continuity and discontinuity in post-Neolithic southern Germany. Pairwise testing for genetic continuity and homogeneity between groups from southern Germany. Coloured

squares depict whether a particular target group (row) can be modelled using a single source group (column). Rank *p*-values higher than 0.01 (corresponding to a single source group) were obtained using generalised-likelihood ratio tests implemented in qpWave with a static set of 11 present-day outgroups.

Modelling present-day Germany

When comparing Iron Age and early medieval samples from Germany to the present-day population using PCA or F_4 statistics, it is evident that the present-day German gene pool cannot be explained as a simple two-way admixture between an *EEF*-enrich Early Iron Age southern source and a *Steppe* (and *WHG*) enriched northern source. Consequently, a qpAdm mixture model using *SGermany_EIA* and the Late Iron Age/Roman period population from Häven, Mecklenburg-Vorpommern, fails. While this population is generally a good proxy for the ancestry found in southern Germany during the Early Middle Ages, representing the pre-Migration period inhabitants of northern Germany, this ancestry cannot account for the high percentage of *WHG*-enriched eastern European ancestry found in a large proportion of present-day Germans (following a west-to-east cline)[58,59].

Previous studies of Y-chromosome haplogroups in the present-day German gene pool showed minor Slavic paternal ancestry (~20%) in modern eastern German[60], indicating that the early medieval Slavic expansion in Europe was a demographic event rather than solely a linguistic spread of the Slavic language.

We observe that most present-day Germans are located in the genetic space between the *SGermany_EIA* and *NGermany_Haeven_LIA* populations, but show additional attraction to a northeastern source in PCA and F_4 statistics (Supp. Fig. 5.7, Supp. Table 4.12). Therefore, we selected prehistoric and historic populations from eastern and northeastern Europe as a potential third source for the qpAdm model described above. Indeed, the inclusion of a third source from northeastern Europe resulted in fitting models (Supp. Table 4.13). When applying this model to 1109 present-day German individuals separately, however, we detect high variability in northeastern European ancestry (Supp. Table 4.14), suggesting that not all parts of present-day Germany were equally affected by the introduction of northeastern Europe, as indicated by previous studies[58–60]. Regarding the timing of this admixture event, we have to assume that it occurred after the initial admixture between *SGermany_EIA* and *NGermany_Haeven_LIA,* probably during the later Early Middle Ages.

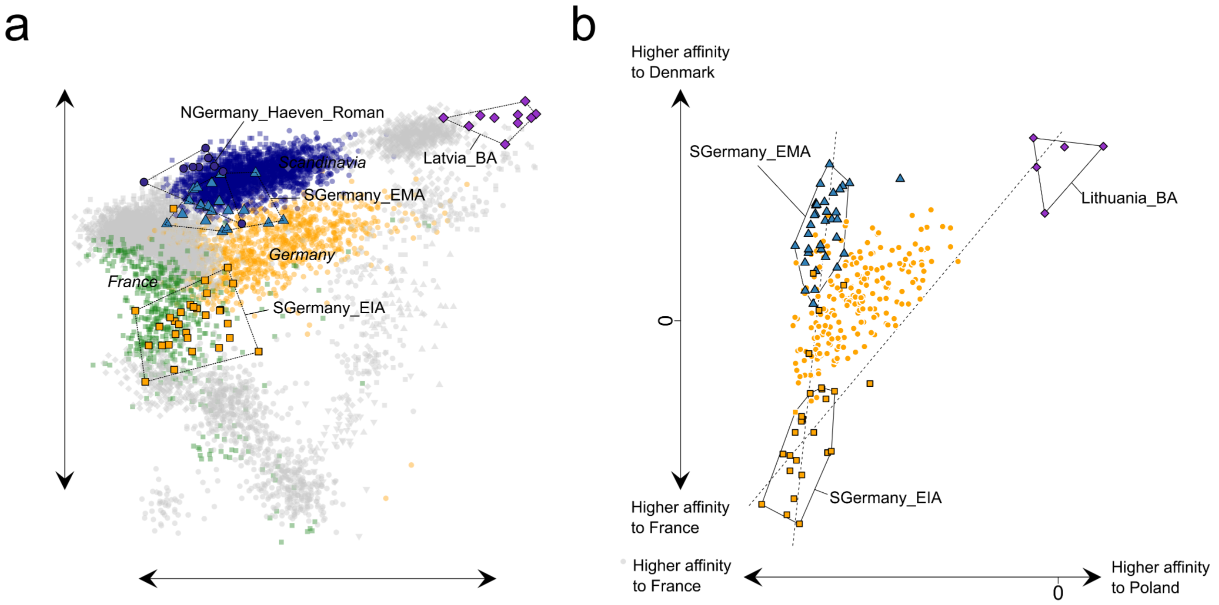

Supplementary Figure 5.7. Principal Component Analysis of European variation and ancient population structure. a) Genetic structure of published and novel ancient individuals in this study, projected onto 3.1a. The present-day populations of France, Germany, and Scandinavia (Denmark, Sweden, and Norway) are highlighted in green, orange, and dark blue, respectively. **b)** Scatterplot of the *F*-statistics of the form F_4(YRI, Test, France, Poland) and F_4(YRI, Test; France, Denmark). 100 randomly selected present-day German genomes are depicted as orange dots.

The end of the Hallstatt Gene pool in the Czech Republic

As described in the main text and Supplementary Note 2, we identify the only La Tène period individual within our dataset, LAN001, as an outlier in terms of excess northern European ancestry. PCA, outgroup F_3 statistics, as well as qpAdm and supervised ADMIXTURE modelling indicate a northwestern European origin for this sample, consistent with its oxygen and strontium signature.
While it is not possible to infer population genetic discontinuity between the Hallstatt and La Tène period on the basis of just one individual, we want to highlight that published samples from the Czech Republic[48] suggest a similar pattern for the eastern Hallstatt sphere.

In our *Northeastern European PCA* (calculated on the following present-day 18 populations: Norway, Sweden, Denmark, Germany, Netherlands, Belgium, Ireland, Scotland, Wales, England, France, Finland, Russia, Belarus, Estonia, Latvia, Lithuania, Poland) it is evident that the groups inhabiting the present-day Czech Republic experienced a substantial diversification in terms of northern European ancestry at the transition from the Hallstatt to the Late Tène period (Supp. Fig. 5.8a). While Hallstatt samples from present-day Czech Republic plot on top of the genetic diversity of present-day French, Belgians and Germans, close to Iron Age individuals from present-day France (Supp. Fig. 5.8b), we demonstrate that during the La Tène period the gene pool diversifies, with several samples showing attraction to present-day northwestern Europeans like Dutch, Danish, and Scandinavians, clustering together with Iron Age/Migration period samples from Lower Saxony, Mecklenburg-Vorpommern, Poland, and Scandinavia (Supp. Fig. 5.8c). This increased affinity to northern European populations is also seen in supervised ADMIXTURE modelling (Supp. Fig. 5.8d, Supp. Table 3.7), suggesting that the groups inhabiting the present-day

Czech Republic intensified their contacts with neighbouring populations to the northwest during the La Tène period (potentially along the amber trade routes), facilitating genetic exchange between those groups.

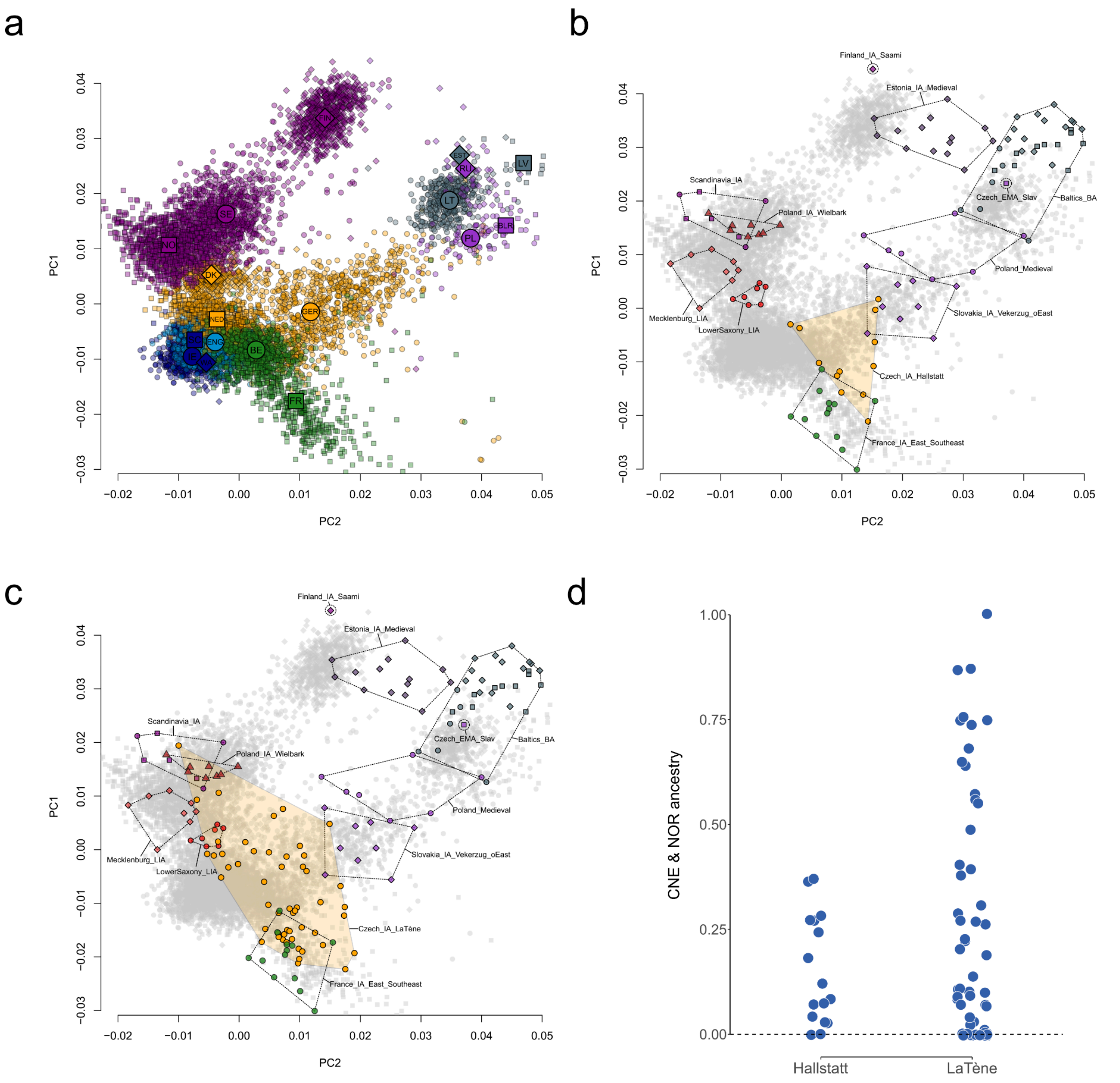

Supplementary Figure 5.8. Population genetic changes in the Czech Republic from the Hallstatt to the La Tène period. a) Principal Components Analysis of present-day genomes from northwestern Europe. IE = Northern Ireland & Ireland, WA = Wales, SC = Scotland, ENG = England, NL = Netherlands, GER = Northern Germany, DK = Denmark, NO = Norway, SE = Sweden, FIN = Finland, BE = Belgium, FR = France, LIT = Lithuania, LV = Latvia, EST = Estonia, POL = Poland, BLR = Belarus, RU = Russia. **b)** Genetic structure of published Hallstatt period samples from the Czech Republic, projected onto a). **c)** same for published La Tène period samples from the Czech Republic. **d)** Individual CNE + NOR ancestry estimates from supervised ADMIXTURE for Hallstatt and La Tène period samples from the Czech Republic.

Supplementary References

1. *Der Magdalenenberg Bei Villingen Im Schwarzwald: Ein Fürstengrabhügel Des 6. Vorchristlichen Jahrhunderts*. vol. 6 (Konrad Theiss, Stuttgart, 1980).
2. Kennett, D. J. *et al.* Archaeogenomic evidence reveals prehistoric matrilineal dynasty. *Nat. Commun.* **8**, 1–9 (2017).
3. Lipatov, M., Sanjeev, K., Patro, R. & Veeramah, K. R. Maximum Likelihood Estimation of Biological Relatedness from Low Coverage Sequencing Data. *bioRxiv* 023374 (2015) doi:10.1101/023374.
4. Ringbauer, H. *et al.* ancIBD - Screening for identity by descent segments in human ancient DNA. *bioRxiv* 2023.03.08.531671 (2023) doi:10.1101/2023.03.08.531671.
5. Gretzinger, J. *et al.* The Anglo-Saxon migration and the formation of the early English gene pool. *Nature* 1–8 (2022).
6. Hummel, S., Schmidt, D. & Herrmann, B. Molekulargenetische Analysen zur Verwandtschaftsfeststellung an Skelettproben aus Gräbern frühkeltischer Fürstensitze. in *Frühkeltische Fürstensitze. Älteste Städte und Herrschaftszentren nördlich der Alpen? Internationaler Workshop zur keltischen Archäologie in Eberdingen-Hochdorf 12. und 13. September 2003* (eds. Biel, J. & Krausse, D.) vol. 51 67–70 (Esslingen, 2005).
7. Steffen, C. *Gesellschaftswandel Während Der älteren Eisenzeit*. vol. 93 (Theiss, Stuttgart, 2012).
8. Katoh, K., Misawa, K., Kuma, K. & Miyata, T. MAFFT: a novel method for rapid multiple sequence alignment based on fast Fourier transform. *Nucleic Acids Res.* **30**, 3059–3066 (2002).
9. Kumar, S., Stecher, G. & Tamura, K. MEGA7: Molecular Evolutionary Genetics Analysis Version 7.0 for Bigger Datasets. *Mol. Biol. Evol.* **33**, 1870–1874 (2016).
10. Posth, C. *et al.* Palaeogenomics of Upper Palaeolithic to Neolithic European hunter-gatherers. *Nature* **615**, 117–126 (2023).
11. Ringbauer, H., Novembre, J. & Steinrücken, M. Parental relatedness through time revealed by runs of homozygosity in ancient DNA. *Nat. Commun.* **12**, 1–11 (2021).
12. Reich, D. *et al.* Reconstructing Native American population history. *Nature* **488**, 370–374 (2012).
13. Haak, W. *et al.* Massive migration from the steppe was a source for Indo-European languages in Europe. *Nature* **522**, 207–211 (2015).

14. Alexander, D. H., Novembre, J. & Lange, K. Fast model-based estimation of ancestry in unrelated individuals. *Genome Res.* **19**, 1655–1664 (2009).
15. Schmid, C. & Schiffels, S. Estimating human mobility in Holocene Western Eurasia with large-scale ancient genomic data. *Proceedings of the National Academy of Sciences* **120**, e2218375120 (2023).
16. Oelze, V. M. *et al.* Multi-isotopic analysis reveals individual mobility and diet at the Early Iron Age monumental tumulus of Magdalenenberg, Germany. *Am. J. Phys. Anthropol.* **148**, 406–421 (2012).
17. Koch, J. K. Vom Schwarzwald zum Mittelmeer und zurück. Fremde Objekte, Individuen und kulturelle Kontakte in der hallstattzeitlichen Gemeinschaft vom Magdalenenbergle. in *Neue Forschungen zum Magdalenenberg* (ed. Krausse, D.) vol. 77 38–52 (Esslingen, 2017).
18. Stephan, E. Rekonstruktion eisenzeitlicher Weidewirtschaft anhand archäozoologischer und isotopenchemischer Untersuchungen. in *Beiträge zur Archäozoologie und Prähistorischen Anthropologie* (ed. Benecke, N.) 65–79 (Beier & Beran, Langenweißbach, 2009).
19. McManus, E. *et al.* 'To the Land or to the Sea': Diet and Mobility in Early Medieval Frisia. *The Journal of Island and Coastal Archaeology* **8**, 255–277 (2013).
20. Jaouen, K. *et al.* Tracing intensive fish and meat consumption using Zn isotope ratios: evidence from a historical Breton population (Rennes, France). *Sci. Rep.* **8**, 1–12 (2018).
21. Willmes, M. *et al.* Mapping of bioavailable strontium isotope ratios in France for archaeological provenance studies. *Appl. Geochem.* **90**, 75–86 (2018).
22. Thomsen, E. & Andreasen, R. Agricultural lime disturbs natural strontium isotope variations: Implications for provenance and migration studies. *Science Advances* **5**, eaav8083 (2019).
23. Breitinger, E. Zur Berechnung der Körperhöhe aus den langen Gliedmaßenknochen. *Anthropol. Anz.* **14**, 249–274 (1937).
24. Erhardt, S. & Simon, P. *Skelettfunde Der Urnenfelder- Und Hallstattkultur in Württemberg Und Hohenzollern*. vol. 9 (Müller & Gräff, Stuttgart, 1971).
25. Wieland, G. & Francken, M. Ein hallstattzeitliches Schwertgrab bei Rosenberg-Sindolsheim (Neckar-Odenwald-Kreis, Baden-Württemberg).
26. Burger-Heinrich, E. Das hallstattzeitliche Gräberfeld von Dittigheim/Tauberbischofsheim. in *Die Hallstattzeit im Nordosten Baden-Württembergs* (ed. Baitinger, H.) vol. 46 409–443 (Theiss,

Stuttgart, 1999).

27. Krausse, D. *et al.* The ‘Keltenblock’ project: discovery and excavation of a rich Hallstatt grave at the Heuneburg, Germany. *Antiquity* **91**, 108–123 (2017).

28. Krausse, D. Vetternwirtschaft? Fragestellung und Design eines archäologisch-paläogenetischen Pilotprojekts zur sozialhistorischen Deutung späthallstattzeitlicher Elitegräber. in *Frühkeltische Fürstensitze. Älteste Städte und Herrschaftszentren nördlich der Alpen? Internationaler Workshop zur keltischen Archäologie in Eberdingen-Hochdorf 12. und 13. September 2003* (eds. Biel, J. & Krausse, D.) vol. 51 63–66 (Esslingen , 2005).

29. Hansen, L. *Die Goldfunde Und Trachtbeigaben Des Späthallstattzeitlichen Fürstengrabes von Eberdingen-Hochdorf (Kr. Ludwigsburg)*. vol. VIII (Landesamt für Denkmalpflege im Regierungspräsidium Stuttgart - Konrad Theiss Verlag, Stuttgart, 2010).

30. Verger, S. Le défunt de la grande tombe celtique de Hochdorf, du chef de famille aristocratique au roi bienheureux. *Histoire, archéologie et société - conférences académiques franco-chinoises* **16**, 36 (2012).

31. Wahl, J., Nehlich, O., Price, T. D. & Pusch, C. M. Fürsten, Fakten, Forschungslücken. Anthropologische Schlaglichter zur Urnenfelder- und Hallstattzeit in Südwestdeutschland. *Aktuelle Forschungen zu den Kelten in Europa. Forschungskolloquium für Landeskonservator Jörg Biel am 1. August 2008 in Altheim* (2010).

32. Biel., J. & Balzer, I. Fürstensitz und mehr. Der Hohenasperg. in *Die Welt der Kelten* (2012).

33. Wahl, J. Karies, Kampf und Schädelkult. 150 Jahre anthropologische Forschung in Südwestdeutschland. *Materialhefte zur Archäologie in Baden-Württemberg* (2007).

34. Wang, R. J., Al-Saffar, S. I., Rogers, J. & Hahn, M. W. Human generation times across the past 250,000 years. *Sci Adv* **9**, eabm7047 (2023).

35. Stan Development Team. *Stan Modeling Language Users Guide and Reference Manual*. https://mc-stan.org (2023).

36. Reichl, J. Estimating marginal likelihoods from the posterior draws through a geometric identity. *Monte Carlo Methods Appl.* **26**, 205–221 (2020).

37. Gronau, Q. F., Singmann, H. & Wagenmakers, E.-J. bridgesampling: An R Package for Estimating Normalizing Constants. *J. Stat. Softw.* **92**, 1–29 (2020).

38. Green, P. J. Reversible Jump Markov Chain Monte Carlo Computation and Bayesian Model

Determination. *Biometrika* **82**, (1995).

39. Newton, M. A. & Raftery, A. E. Approximate Bayesian inference with the weighted likelihood bootstrap. *J. R. Stat. Soc.* **56**, 3–26 (1994).
40. Rohrlach, A. B., Tuke, J., Popli, D. & Haak, W. BREADR: An R Package for the Bayesian Estimation of Genetic Relatedness from Low-coverage Genotype Data. *bioRxiv* 2023.04.17.537144 (2023) doi:10.1101/2023.04.17.537144.
41. Fu, Q. *et al.* A Revised Timescale for Human Evolution Based on Ancient Mitochondrial Genomes. *Curr. Biol.* 1–7 (2013).
42. Karmin, M. *et al.* A recent bottleneck of Y chromosome diversity coincides with a global change in culture. *Genome Res.* 1–9 (2015).
43. Mittnik, A. *et al.* Kinship-based social inequality in Bronze Age Europe. *Science* **366**, 731–734 (2019).
44. Rivollat, M. *et al.* Extensive pedigrees reveal the social organization of a Neolithic community. *Nature* **620**, 600–606 (2023).
45. Patterson, N. *et al.* Ancient admixture in human history. *Genetics* **192**, 1065–1093 (2012).
46. Holsinger, K. E. & Weir, B. S. Genetics in geographically structured populations: defining, estimating and interpreting F ST. *Nat. Rev. Genet.* **10**, 639–650 (2009).
47. Parzinger, H. & Stegmann-Rajtár, S. Smolenice-Molpír und der Beginn skythischer Sachkultur in der Südwestslowakei. *Praehist. Z.* **63**, (1988).
48. Patterson, N. *et al.* Large-scale migration into Britain during the Middle to Late Bronze Age. *Nature* **601**, 588–594 (2021).
49. Chintalapati, M., Patterson, N. & Moorjani, P. The spatiotemporal patterns of major human admixture events during the European Holocene. *Elife* **11**, (2022).
50. Rivollat, M. *et al.* Ancient genome-wide DNA from France highlights the complexity of interactions between Mesolithic hunter-gatherers and Neolithic farmers. *Science Advances* **6**, eaaz5344 (2020).
51. Keller, A. *et al.* New insights into the Tyrolean Iceman's origin and phenotype as inferred by whole-genome sequencing. *Nat. Commun.* **3**, 1–9 (2012).
52. Sikora, M. *et al.* Population Genomic Analysis of Ancient and Modern Genomes Yields New Insights into the Genetic Ancestry of the Tyrolean Iceman and the Genetic Structure of Europe.

PLoS Genet. **10**, e1004353 (2014).

53. Marcus, J. H. *et al.* Genetic history from the Middle Neolithic to present on the Mediterranean island of Sardinia. *Nat. Commun.* **11**, 1–14 (2020).
54. Lipson, M. *et al.* Parallel palaeogenomic transects reveal complex genetic history of early European farmers. *Nature* **551**, 368–372 (2017).
55. Mathieson, I. *et al.* Genome-wide patterns of selection in 230 ancient Eurasians. *Nature* **528**, 499–503 (2015).
56. Veeramah, K. R. *et al.* Population genomic analysis of elongated skulls reveals extensive female-biased immigration in Early Medieval Bavaria. *Proc. Natl. Acad. Sci. U. S. A.* **115**, 3494–3499 (2018).
57. O'Sullivan, N. *et al.* Ancient genome-wide analyses infer kinship structure in an Early Medieval Alemannic graveyard. *Sci Adv* **4**, eaao1262 (2018).
58. Kayser, M. *et al.* Significant genetic differentiation between Poland and Germany follows present-day political borders, as revealed by Y-chromosome analysis. *Hum. Genet.* **117**, 428–443 (2005).
59. Roewer, L. *et al.* Signature of recent historical events in the European Y-chromosomal STR haplotype distribution. *Hum. Genet.* **116**, 279–291 (2005).
60. Rębała, K. *et al.* Contemporary paternal genetic landscape of Polish and German populations: from early medieval Slavic expansion to post-World War II resettlements. *Eur. J. Hum. Genet.* **21**, 415–422 (2012).
