# Supplementary material for: Evidence for dynastic succession among early Celtic elites in Central Europe
Source: Nat Hum Behav. 2024 Jun 3;8(8):1467–80. doi: 10.1038/s41562-024-01888-7 (PMC11343710; doi:10.1038/s41562-024-01888-7)
Supplement: Supplementary file 2 — Reporting Summary [file 41562_2024_1888_MOESM2_ESM.pdf]

Corresponding author(s): Dirk Krausse, Johannes Krause, Stephan Schiffels

Last updated by author(s): Aug 31, 2023

Reporting Summary

Nature Portfolio wishes to improve the reproducibility of the work that we publish. This form provides structure for consistency and transparency in reporting. For further information on Nature Portfolio policies, see our Editorial Policies and the Editorial Policy Checklist.

Statistics

For all statistical analyses, confirm that the following items are present in the figure legend, table legend, main text, or Methods section.

n/a	Confirmed	
☐	☒	The exact sample size (*n*) for each experimental group/condition, given as a discrete number and unit of measurement
☒	☐	A statement on whether measurements were taken from distinct samples or whether the same sample was measured repeatedly
☐	☒	The statistical test(s) used AND whether they are one- or two-sided<br>*Only common tests should be described solely by name; describe more complex techniques in the Methods section.*
☒	☐	A description of all covariates tested
☐	☒	A description of any assumptions or corrections, such as tests of normality and adjustment for multiple comparisons
☐	☒	A full description of the statistical parameters including central tendency (e.g. means) or other basic estimates (e.g. regression coefficient) AND variation (e.g. standard deviation) or associated estimates of uncertainty (e.g. confidence intervals)
☐	☒	For null hypothesis testing, the test statistic (e.g. *F*, *t*, *r*) with confidence intervals, effect sizes, degrees of freedom and *P* value noted<br>*Give P values as exact values whenever suitable.*
☐	☒	For Bayesian analysis, information on the choice of priors and Markov chain Monte Carlo settings
☒	☐	For hierarchical and complex designs, identification of the appropriate level for tests and full reporting of outcomes
☐	☒	Estimates of effect sizes (e.g. Cohen's *d*, Pearson's *r*), indicating how they were calculated

Our web collection on statistics for biologists contains articles on many of the points above.

Software and code

Policy information about availability of computer code

Data collection	No specific software was used for sample collection. Genotype data were generated from sequencing reads. All software used in this study is listed below.
Data analysis	All software used in this work is publicly available. Custom code developed for the Bayesian pedigree modelling (Supplementary Text 3) is described and available at https://github.com/stschiff/celtic_relationship_analysis. An archived version is available on zenodo (10.5281/zenodo.10427675). Corresponding publications are cited in the main text and supplementary material. List of software and respective versions: AdapterRemoval (v2.3.1), Burrows-Wheeler Aligner (v0.7.12), DeDup (v0.12.2), mapDamage (v2.0.6), BamUtil (v1.0.14), EAGER (v1), Sex.DetERRmine (v1.1.2) (https://github.com/TCLamnidis/Sex.DetERRmine), ANGSD (v0.923), Schmutzi (v1.5.4), PMDtools (v0.50), pileupCaller (v1.4.0.2), samtools (v1.3.1), Geneious R9.8.1, HaploGrep 2 (v2.4.0), READ (https://bitbucket.org/tguenther/read) (vf541d55), lcMLkin (https://github.com/COMBINE-lab/maximum-likelihood-relatedness-estimation) (v0.5.0), PLINK (v1.90b3.29), Picard tools (v2.27.3), smartpca (v16000; EIGENSOFT v6.0.1), qp3Pop (v.435; ADMIXTOOLS v3.0), qpDstat (v.755; ADMIXTOOLS v3.0), qpWave (v410), qpAdm (v.810), hapROH (v0.6), DATES (v4010), ADMIXTURE (v1.3), KIN (v3.1.3), ancIBD (https://pypi.org/project/ancIBD) (v0.4), GLIMPSE (https://github.com/odelaneau/GLIMPSE) (v2.0.0), BREADR (https://github.com/jonotuke/BREADR) (746316f), MOBEST (https://github.com/nevrome/mobest.analysis.2022) (v26f929e), MAFFT (v6.864), MEGA (v7). Data visualisation and descriptive statistical tests were performed in R (v4.1.1). The following R packages were used: Rsamtools (v2.12.0), binom (v1.1-1.1), ape (v.5.6-2), phytools (v1.0-3), psych (v2.2.5), vegan (v2.6-2), factoextra (v1.0.7), ggplot2 (v3.3.6), ggExtra (v0.10.0), ggforce (v0.3.3), rnaturalearth (v0.1.0), sf (v1.0.-8), raster (v3.5-21), elevatr (v0.4.2), rgdal (v1.5-32), spatstat (v2.3-4), maptools (v1.1-4), gstat (v2.0-9), sp (v1.5-0), labdsv (v2.0-1), igraph (v1.3.4), magrittr (v2.0.3), dplyr (v1.0.9), reshape 2 (v1.4.4), and tidyverse (v.1.3.2). Y-chromosome and mtDNA haplogroups were determined using the ISOGG SNP index (v15.73) and PhyloTree (v17-FU1) reference databases, respectively.

For manuscripts utilizing custom algorithms or software that are central to the research but not yet described in published literature, software must be made available to editors and reviewers. We strongly encourage code deposition in a community repository (e.g. GitHub). See the Nature Portfolio guidelines for submitting code & software for further information.

Data

Policy information about availability of data

All manuscripts must include a data availability statement. This statement should provide the following information, where applicable:

- Accession codes, unique identifiers, or web links for publicly available datasets
- A description of any restrictions on data availability
- For clinical datasets or third party data, please ensure that the statement adheres to our policy

Raw sequence data (fastq files) from the 31 newly reported ancient individuals will be available prior publication from the European Nucleotide Archive under accession number PRJEB73566. Published genotype data for the present-day British sample are available from the WTCCC via the European Genotype Archive (https://www.ebi.ac.uk/ega/) under accession number EGAD00010000634. Published genotype data for the present-day Irish sample are available from the WTCCC via the European Genotype Archive under accession number EGAD00010000124. Published genotype data for the rest of the present-day European samples are available from the WTCCC via the European Genotype Archive under accession number EGAD00000000120. Published genotype data for the Dutch samples are available by the GoNL request process from The Genome of the Netherlands Data Access Committee (DAC) (https://www.nlgenome.nl). The Genome Reference Consortium Human Build 37 (GRCh37) is available via the National Center for Biotechnology Information under accession number PRJNA31257. The revised Cambridge reference sequence is available via the National Center for Biotechnology Information under NCBI Reference Sequence NC_012920.1. Previous published genotype data for ancient individuals was reported by the Reich Lab in the Allen Ancient DNA Resource v.54.1(https://reich.hms.harvard.edu/allen-ancient-dna-resource-aadr-downloadable-genotypes-present-day-and-ancient-dna-data).

Research involving human participants, their data, or biological material

Policy information about studies with human participants or human data. See also policy information about sex, gender (identity/presentation), and sexual orientation and race, ethnicity and racism.

Reporting on sex and gender	Does not apply. This study does not include novel data from present-day humans or any present-day human participants.
Reporting on race, ethnicity, or other socially relevant groupings	Does not apply. This study does not include novel data from present-day humans or any present-day human participants.
Population characteristics	Does not apply. This study does not include novel data from present-day humans or any present-day human participants.
Recruitment	Does not apply. This study does not include novel data from present-day humans or any present-day human participants.
Ethics oversight	Does not apply. This study does not include novel data from present-day humans or any present-day human participants.

Note that full information on the approval of the study protocol must also be provided in the manuscript.

Field-specific reporting

Please select the one below that is the best fit for your research. If you are not sure, read the appropriate sections before making your selection.

☒ Life sciences ☐ Behavioural & social sciences ☐ Ecological, evolutionary & environmental sciences

For a reference copy of the document with all sections, see nature.com/documents/nr-reporting-summary-flat.pdf

Life sciences study design

All studies must disclose on these points even when the disclosure is negative.

Sample size	We did not rely on statistical methods to predetermine sample sizes. Sample sizes for ancient populations depended solely on the availability of archaeological material and on ancient DNA preservation. For present-day populations, sample sizes are predefined by the availability of published data. In our study, most present-day populations are represented by more than 100 genomes. The selection of samples and sample size calculation for these data is described in the source publications and follows the established guidelines in medical genetics. For both ancient and present-day populations, we aim to maximizes sample sizes by including all genomes that fulfil our quality criteria mentioned in the Methods section and the quality criteria described in their respective source publications. The reported standard errors used to describe uncertainty ranges of our statistical analyses often reflect both sample size and data quality per sample.
Data exclusions	One sample (SCN001) was excluded from genome-wide analyses since the authenticity of the autosomal ancient DNA data could not be ensured. The quality criteria forming the basis of this decision are mentioned in the Methods section are pre-established by various previous publications.
Replication	We studied unique entities (past and present populations) and did not perform experiments or study various treatments, so replication is not applicable. But we note that samples from the same population carry similar genetic signatures. For the four samples HOC001, APG001, APG003 and MBG009, we produced complementary to the partial UDG-treated, double-stranded DNA libraries also non UDG-treated single-stranded libraries to increase the genome-wide coverage. While the proportion of authentic ancient DNA obtained from the single-stranded libraries is generally comparable to the proportion measured in the double-stranded libraries, the combination of genotypes from both sources substantially increases the coverage of the respective genomes. Testing of pairwise mismatches in 1,24 Million SNP sites between two

libraries of the same individual confirm that DNA sequences from both double- and single-stranded libraries are indeed identical. Also, genome-wide data allows for the analysis of multiple realisations of the sample history, by studying hundreds of thousands of SNP sites.

Randomization	We studied unique entities (past and present populations) and did not perform experiments or study various treatments, so randomization is not applicable. However, many of our analyses based on f-statistics involve a block jackknife to obtain uncertainty ranges through analysis of uncorrelated segments of the genome.
Blinding	We studied unique entities (past and present populations) and did not perform experiments or study various treatments, so blinding is not applicable.

Reporting for specific materials, systems and methods

We require information from authors about some types of materials, experimental systems and methods used in many studies. Here, indicate whether each material, system or method listed is relevant to your study. If you are not sure if a list item applies to your research, read the appropriate section before selecting a response.

Materials & experimental systems

n/a	Involved in the study	
☒	☐	Antibodies
☒	☐	Eukaryotic cell lines
☐	☒	Palaeontology and archaeology
☒	☐	Animals and other organisms
☒	☐	Clinical data
☒	☐	Dual use research of concern
☒	☐	Plants

Methods

n/a	Involved in the study	
☒	☐	ChIP-seq
☒	☐	Flow cytometry
☒	☐	MRI-based neuroimaging

Palaeontology and Archaeology

Specimen provenance	All sampled bone material belongs to the Landesamt für Denkmalpflege Baden-Württemberg, an institution of the Federal Republic of Germany. This donating partner institution is represented in the author list. Permits for destructive analyses were given explicitly
Specimen deposition	Specimens were returned to the owning institutions after laboratory analyses.
Dating methods	No new dates are provided.

☐ Tick this box to confirm that the raw and calibrated dates are available in the paper or in Supplementary Information.

Ethics oversight	No ethical oversight was required strictly. However, we confirm that all analyses followed established ethical guidelines for archaeogenetic research, as detailed in Wagner et al., AJHG, 2020 and Alpaslan-Roodenberg, Nature, 2021.

Note that full information on the approval of the study protocol must also be provided in the manuscript.
